# Univariate Community Assembly Analysis (UniCAA): Combining hierarchical models with null models to test the influence of spatially restricted dispersal, environmental filtering, and stochasticity on community assembly

**DOI:** 10.1002/ece3.4868

**Published:** 2019-01-13

**Authors:** Markus A. K. Sydenham, Stein R. Moe, Mari Steinert, Katrine Eldegard

**Affiliations:** ^1^ Faculty of Environmental Sciences and Natural Resource Management Norwegian University of Life Sciences Ås Norway

**Keywords:** community assembly, dispersal, environmental filtering, stochasticity, traits

## Abstract

Identifying the influence of stochastic processes and of deterministic processes, such as dispersal of individuals of different species and trait‐based environmental filtering, has long been a challenge in studies of community assembly. Here, we present the Univariate Community Assembly Analysis (UniCAA) and test its ability to address three hypotheses: species occurrences within communities are (a) limited by spatially restricted dispersal; (b) environmentally filtered; or (c) the outcome of stochasticity—so that as community size decreases—species that are common outside a local community have a disproportionately higher probability of occurrence than rare species. The comparison with a null model allows assessing if the influence of each of the three processes differs from what one would expect under a purely stochastic distribution of species. We tested the framework by simulating “empirical” metacommunities under 15 scenarios that differed with respect to the strengths of spatially restricted dispersal (restricted vs. not restricted); habitat isolation (low, intermediate, and high immigration rates); and environmental filtering (strong, intermediate, and no filtering). Through these tests, we found that UniCAA rarely produced false positives for the influence of the three processes, yielding a type‐I error rate ≤5%. The type‐II error rate, that is, production of false negatives, was also acceptable and within the typical cutoff (20%). We demonstrate that the UniCAA provides a flexible framework for retrieving the processes behind community assembly and propose avenues for future developments of the framework.

## 1 INTRODUCTION

Understanding how and why the number and identities of species vary among habitats is a central goal in ecology. Species distributions are the product of assembly processes whereby species disperse across the landscape and establish populations in habitats—within reach—where the environment provides suitable conditions (Keddy, 1992). Once established, biotic interactions, such as competition, determine how successful the newly arrived species will be, and thus influence abundances (Boulangeat, Gravel, & Thullier, 2012) and—in the long‐term—species richness (Olsen & Klanderud, 2014). However, even if species have similar habitat requirements and are competitively equivalent, species richness will not remain stable since random fluctuations in population growth‐rates eventually lead to monodominance by the initially most abundant species (Hubbell, 2001; Rosindell, Hubbell, & Etienne, 2011). If species can disperse between communities at high rates, the influence of such stochastic processes can be synchronized at the metacommunity level, and result in purely stochastic species distributions. Mechanisms of community assembly thus can be classified as belonging to: (a) dispersal limitation resulting from (i) habitat isolation, leading to low immigration rates so that local community dynamics are partly independent of metacommunity dynamics and (ii) spatially restricted dispersal of species, leading to low spatial immigration rates because potential immigrants mainly arrive from proximate source populations; (b) ecological filtering based on how the species’ fitness varies according to biotic and abiotic environmental conditions; and (c) stochastic processes, such as ecological drift (Vellend, 2016). These assembly processes can interact and reinforce each other. High immigration rates may reduce the influence of environmental filtering, and thus lead to mass‐effect metacommunities. In contrast, if environmental filtering is the dominant process, this leads to species‐sorting metacommunities (Leibold et al., 2004). The influence of stochastic processes is also influenced by dispersal limitation and are expected to decrease as immigration increase (Vellend, 2016) since high immigration rates replenish the populations of rare species, thereby allowing them to persist over time (Hanski, 1991). Community size, that is, the number of individuals of all species, is a proxy for the carrying capacity of the local habitat. Because ecological drift is a probabilistic process, its influence increases as community size decreases (Gilbert & Levine, 2017; Vellend, 2016). In neutral metacommunities with high immigration rates, the effect of ecological drift will be synchronized at the metacommunity level, so that the relative abundance of species within local communities mirrors that of the metacommunity as a whole (Shipley, 2014). In lieu of environmental filtering and dispersal limitation, species distributions will therefore be purely stochastic. Due to their complexities, identifying the processes behind patterns of species distributions remains a central challenge in ecology (Cadotte & Tucker, 2017).

Dispersal limitation restricts the flow of species across the landscape, and therefore results in spatially aggregated species distributions and increased species compositional dissimilarity (i.e., β‐diversity) between communities with increasing geographical distance (Anderson et al., 2011; Chave & Leigh, 2002). Ecological filtering may also cause species to aggregate into classifiable communities. A distinction is made between biotic ecological filtering, such as competition, and abiotic ecological filtering (hereafter “environmental filtering”). Environmental filtering operates by excluding species whose functional response traits do not allow them to persist within a habitat, and result in species distributions being predictable along environmental gradients (Keddy, 1992; Kraft et al., 2015; McGill, Enquist, Weiher, & Westoby, 2006). Here, we focus on environmental filters, because they determine the potential combination of species within communities, upon which biotic interactions in turn operate (Boulangeat et al., 2012; Lawton, 1999). The influence of environmental filtering versus ecological drift (and other stochastic processes) can be estimated by comparing observed β‐diversity values between communities with those obtained from null models in which community assembly is neutral with regards to species identities (Chase & Myers, 2011; Tucker, Shoemaker, Davies, Nemergut, & Melbourne, 2016). However, because the environmental filtering and stochasticity often act in concert with dispersal limitation, the influence of all three processes should ideally be captured in the same analysis.

Current methods for disentangling the effects of the three community assembly processes (reviewed in Vellend et al., 2014) include: partitioning the variation in species composition along gradients of spatial and environmental dissimilarity (Peres‐Neto, Legendre, Dray, & Borcard, 2006); comparing changes in the functional and species turnover along environmental and spatial gradients (Pavoine & Bonsall, 2011); and parallel analyses of, for example, phylogenetic, functional and species diversity indices (Münkemüller et al., 2012). An alternative approach is to focus on species occurrences (or abundances) rather than species composition. By combining matrices that contain information on species distributions, environmental conditions, and species traits, ecologists can test for trait–environment relationships (Dray et al., 2014; Dray & Legendre, 2008). Model‐based approaches that allow explicit testing of how community assembly processes influence species occurrences or abundances have recently been developed (Ovaskainen et al., 2017; Warton et al., 2015). These model‐based approaches focus on the distribution of species (or individuals) as a function of their traits, rather than modeling changes in trait values as a function of species distributions along environmental gradients. Using the presence (or absence) of species as response variables in statistical models, and including interaction terms between functional traits and site‐specific environmental variables as explanatory variables, makes it possible to test the influence of environmental filtering (Jamil, Ozinga, Kleyer, & Braak, 2013). Existing methods allow estimating the relative importance of spatially restricted dispersal, environmental filtering, and biotic interactions for species occurrences and abundances within local communities (Boulangeat et al., 2012; Ovaskainen et al., 2017). An important limitation of current approaches is that they either test the influence of spatially restricted dispersal versus environmental filtering, or stochasticity versus environmental filtering, but not all three processes simultaneously (but see Munoz et al., 2018 for estimating the influence of immigration rates together with stochasticity and environmental filtering).

Here, we present a framework for simultaneously testing the influence of spatially restricted dispersal, environmental filtering, and stochasticity on species occurrences in terrestrial ecosystems, hereafter UniCAA (Univariate Community Assembly Analysis). The approach builds on the framework developed by Sydenham et al. (2017), who modeled the occurrence of wild bee species in southeast Norway. In the present study, we used simulated data generated under 15 distinct parameter state combinations with varying degrees of spatially restricted dispersal; immigration rates; and environmental filtering to assess the applicability of UniCAA, based on its ability to identify:

1. Spatially restricted dispersal in metacommunities, in cases where species migrations are most likely between proximate habitat patches. The influence of spatially restricted dispersal on metacommunity structure can take three primary forms (Leibold & Chase, 2018): Dispersal limitation whereby species fail to occupy all potential habitats within the metacommunity; Dispersal sufficiency where dispersal rates are intermediate and species occur in the majority of suitable habitats; and Dispersal surplus whereby dispersal and immigration rates are sufficiently high to mask the influence of species‐sorting mechanisms (e.g., environmental filtering). Under dispersal limitation, spatially restricted dispersal results in spatially aggregated species distributions and should be most pronounced in metacommunities with a high temporal species turnover (i.e., high immigration rates) and with ecologically equivalent species, because environmental filtering otherwise prevents dispersing species from establishing within communities. UniCAA should not confound spatially restricted dispersal with environmental filtering and produce false positives (type‐I errors) in metacommunities, if species are free to disperse but environmental conditions are spatially correlated.
2. Environmental filtering in metacommunities where species have narrow niche widths, and thereby lower probability of remaining in habitats with environmental conditions outside their fundamental niche. In such cases, the probability of occurrence should differ systematically between species, depending on their functional traits and local environmental conditions. The role of environmental filtering can be obscured if immigration rates are sufficiently high, that is, under mass‐effect metacommunities (Leibold et al., 2004). In such cases, habitats may be occupied by species that are not adapted to local environmental conditions. However, such habitats should act as “sink‐habitats” and—on average—have a lower probability of containing species whose traits do not match local conditions than species whose traits do match the local conditions.
3. Identify stochasticity in metacommunities in cases where species are ecologically equivalent and not dispersal limited. Stochastic dynamics should be synchronized at the metacommunity level when immigration rates are high and when species are ecologically equivalent. Deviations from the patterns expected under stochastic species distributions suggest that communities are dispersal limited or environmentally filtered, so that local community dynamics are at least partly independent of the dynamics in distant or environmentally different communities.

## 2 METHODS

### 2.1 The UniCAA framework

UniCAA uses Generalized Linear Mixed Models (GLMMs) with the probability of species occurring within communities as a response variable. UniCAA differs from other model‐based approaches (Hui, 2016; Ovaskainen et al., 2017; Warton et al., 2015) in two important aspects; (a) Spatially restricted dispersal is modeled as a fixed effect and as function of the species‐specific geographic distance to the nearest source population. This adds flexibility in that users can specify species‐specific distance matrices based on prior information on barriers to dispersal, and that the geographic distance can be transformed to improve model fit. (b) UniCAA compares the influence of the three community assembly processes to that expected from a null model, thus allowing an assessment of whether the observed influence of each process differs from what would be expected under stochastic community assembly. Species and site identities are included as random intercepts in the model, to account for multiple observations from the same sites and species. Thus, the modeled response is the probability of occurrence of an average species in an average site given the constraints imposed by spatially restricted dispersal, environmental filtering, and stochastic processes (Table 1).

1. The influence of spatially restricted dispersal is tested by including the fixed effect term Distance to source habitat, which for all species‐by‐site combinations specifies the geographic distance to the nearest site where the species is found (Sydenham et al., 2017). A decrease in the mean probability of occurrence with Distance to source habitat would suggest that species are spatially aggregated, so that the mean probability of occurrence decreases with the geographic distance to the nearest community from which the species could immigrate (MacArthur & Wilson, 1967).
2. The influence of environmental filters is tested by including Traits × Environmental conditions terms, that is, interactions between the environmental conditions and functional traits (e.g., body size) of species (Jamil et al., 2013). If community assembly is environmentally filtered, the probability of species occurring within communities depends on the environmental conditions and differs systematically between species depending on their functional traits (Keddy, 1992).
3. The influence of stochasticity is tested by including the interaction term Community size × Commonness, that is, between the total number of individuals sampled within a given site (Community size) and the proportionate contribution of a species to the total number of individuals found outside a given community (Commonness). The influence of stochastic community assembly is here understood as leading to patterns of species occurrence that are solely probabilistic. If species are ecologically equivalent and not subjected to spatially restricted dispersal, then—on average—the relative abundance of species within communities should mirror that of the regional species pool (Shipley, 2014; Vellend, 2016). In UniCAA, the regional species pool is defined from the species composition of the set of sampled communities. We therefore expect regionally rare species to have a lower probability of occurrence within small communities than common species. As community size increases, regionally common species should always be present, whereas rare species will have an increased, but not definite, probability of occurrence.

**Table 1:** Illustration of the four data frames used in UniCAA. (a) The site‐by‐species (Sp.) data frame contains the abundance of the *i*th species at the *j*th site. (b) The species‐by‐trait data frame contains the *i*th species’ functional trait value for each *n*th trait. (c) The site‐by‐environment (Env.) data frame contains the environmental variable values (ecological filter) for the *j*th site. (d) The site‐by‐spatial geographic positions data frame contains the geographical coordinates for each *j*th site. (e) The UniCAA.df dataframe is used for the analyses in step 1. Prior to analyses, species only occurring within a single site are removed because the *Distance to source habitat *variable will return a missing value. See Supporting information Appendix S1 for a fully worked example

a						b		c			d		
	Sp. A	Sp. B	Sp. C			Trait			Env.			Lat.	Lon.
Site 1	10_A1_	5 _B1_	0 _C1_		Sp. A	0		Site 1	0		Site 1	1	1
Site 2	5 _A2_	10 _B2_	5 _C2_		Sp. B	0.5		Site 2	0		Site 2	2	1
Site 3	0 _A3_	5 _B3_	10 _C3_		Sp. C	1		Site 3	1		Site 3	3	1
Site 4	0 _A4_	0 _B4_	5 _C4_					Site 4	1		Site 3	4	1

e							
Presence	Dist. to source habitat	Trait	Env.	ComSize	Commonness	SpeciesID	SiteID
1	1	0	0	15	0.125	Sp. A	Site 1
1	1	0	0	20	0.286	Sp. A	Site 2
…	…	…	…	…	…	…	…
1	1	1	1	15	0.250	Sp. C	Site 3
1	1	1	1	5	0.300	Sp. C	Site 4

Note

Dist. to source habitat is the distance from the *j*th community to the nearest community where the *i*th species is found. Inter‐site distances are calculated using the spatial coordinates information in data frame **d**. For the combination Sp. A and Site 1 the Dist. to source habitat is 1 because Sp. A is found in Site 2, which is only one step away from Site 1. Community size (ComSize) is the total number of individuals within a community, calculated from data frame **a**. The ComSize of site 1 is: 10_A1 _+ 5_B1 _+ 0_C1_ = 15. Commonness is calculated using the information in data frame **a** as the proportionate contribution of the focal species to all individuals sampled outside the focal site, for example, the Commonness of species A outside site 1 is: 5_A2_/(5_A2_ + 10_B2_ + 5_C2_ + 5_B3_ + 10_C3_ + 5_C4_) = 0.125.

### 2.2 Step 1 model specifications

The influence of spatially restricted dispersal, environmental filtering, and stochasticity on species occurrences is first tested by fitting separate GLMMs and using likelihood ratio tests to assess the statistical significance (*α* = 0.05) of Distance to source habitat, Traits × Environmental conditions and Community size × Regional commonness, respectively. Subsequently, a full model containing only the significant terms from the three separate GLMMs is built. The full model is then reduced to a final model through backward elimination of variables, retaining only those with significant contributions to model fit. In the case where species occurrences are dispersal limited, subjected to environmental filtering and stochasticity, the final model formula becomes:

$$Y_{\mathrm{ij}}=\text{Bin}\left(1,p_{\mathrm{ij}}\right)$$

$$\begin{array}{c} \text{logit}\left(p_{\mathrm{ij}}\right)=\alpha +\beta_{1}\times Distance\;to\;source\;habitat_{\mathrm{ij}}+\beta_{{}_{2}}\times Environmental\;filter_{j}\\ +\beta_{3}\times Species\;trait_{i}+\beta_{4}\times Species\;trait_{i}\\ \times Environmental\;conditions_{j}+\beta_{5}\times Community\;size_{j}\\ +\beta_{6}\times Commonness_{i}+\beta_{7}\times Community\;size_{j}\\ \times Commonness_{i}+Species\;identity_{i}+Site\;identity_{{}_{j}} \end{array}$$

$$Species\;identity_{i}∼N\left(0,\sigma_{\alpha }^{2}\right)$$

$$Site\;identity_{{}_{i}}∼N\left(0,\sigma_{\alpha }^{2}\right)$$

where *Y_ij_* is the probability of the *i*th species being present in the *j*th site. Distance to source habitat, Environmental conditions, Species traits, Community size, and Commonness are fixed effect terms, whereas Species identity and Site identity are random intercept terms (Zuur, Ieno, Walker, Saveliev, & Smith, 2009). Although individual species may show unimodal responses to the environmental gradient(s), the Species traits × Environmental conditions term models the average occurrence of species, with a given trait value as a function of the environment. Depending on the combined niche width of species belonging to a trait group, the mean occurrence of species within that trait group can be expected to be linear or unimodal. Misspecified models should result in non‐normally distributed residuals around the predicted estimates for species occurrences. The residual distribution of binomial GLMMs can be assessed using the DHARMa package in R (Hartig, 2018). As in the model selection, the statistical significance of the main effect terms in the model can be tested using likelihood ratio tests.

### 2.3 Step 2 model specifications

To assess whether the observed relationship between species occurrences and Distance to source habitat, Species traits × Environmental conditions, and Community size × Commonness differ from that expected under stochastic community assembly, the regression coefficients from the fixed effect terms in the final model (step 1) are compared to those obtained from a null model. In the null model, species are ecologically equivalent, immigration rates are high and species are free to disperse across the entire landscape—that is, species distributions are purely stochastic—resulting in a neutral metacommunity (sensu Leibold et al., 2004). In step 2, the final model from step 1 is refitted:

$$\begin{array}{c} \text{logit}\left(p_{\mathrm{ij}}\right)=\alpha +(\beta_{1}\times Distance\;to\;source\;habitat_{\mathrm{ij}}\\ +\beta_{{}_{2}}\times Environmental\;filter_{j}+\beta_{3}\times Species\;trait_{i}\\ +\beta_{4}\times Species\;trait_{i}\times Environmental\;conditions_{j}\\ +\beta_{5}\times Community\;size_{j}+\beta_{6}\times Commonness_{i}\\ +\beta_{7}\times Community\;size_{j}\times Commonness_{i})\\ \times \;Data\;source\\ +Species\;identity_{i}/DatasetID\\ +Site\;identity_{{}_{j}}/DatasetID \end{array}$$

where Data source is a categorical variable with two levels: empirical data or data from the null model (simulated data), ensuring that the null model does not affect parameter estimates for the empirical data. Dataset ID is a categorical variable specifying the identity of the data in the model. Dataset #1 is the empirical data, whereas each of the simulated metacommunities making up the null model is assigned a unique identifier. The random effects thereby become crossed that is: Species identity given Dataset ID; and Site identity given Dataset ID so that the number of groups for which the random effects are estimated, increase with the size of the null model*.*

The null model is constructed by reshuffling the original species‐by‐site data frame while keeping the row and column sums constant. This null model retains the species’ relative abundances in the whole metacommunity as well as community sizes. Multiple randomizations are required because metacommunities will differ between different randomizations and because we are interested in obtaining a null model with parameter estimates reflecting the “average” randomized metacommunity. The computation time for fitting the model in step 2 will increase with the number of randomizations used when specifying the null model (i.e., Dataset IDs). Our fn.*UniCAA.sim.eval* function (Supporting information Appendix S1) makes it possible to evaluate how many (e.g., 19, 49, 99, or 199) randomizations are required by plotting the β‐diversity between each randomized metacommunity and the empirical metacommunity against the randomization number (Dataset ID). The null model has saturated when there is no detectable relationship between β‐diversity and randomization number.

Each of the randomized site‐by‐species matrices is combined with the original site‐by‐environment, site‐by‐coordinates, and species‐by‐traits matrices into UniCAA.df data frames by using the *fn.UniCAA.df* function (Supporting information Appendix S1). The UniCAA.df data frames are then merged with the empirical UniCAA.df data frame and two columns are added: the Dataset ID column contains a unique identifier for each of the, for example, 100 datasets (99 simulated + 1 empirical), and the Data source column contains a categorical variable with two levels (empirical or simulated). Statistically significant deviations from the null model suggest that the observed (empirical) community compositions differ from that expected if species were ecologically equivalent and free to disperse across the entire region.

### 2.4 Testing the UniCAA framework

**Figure 1:**
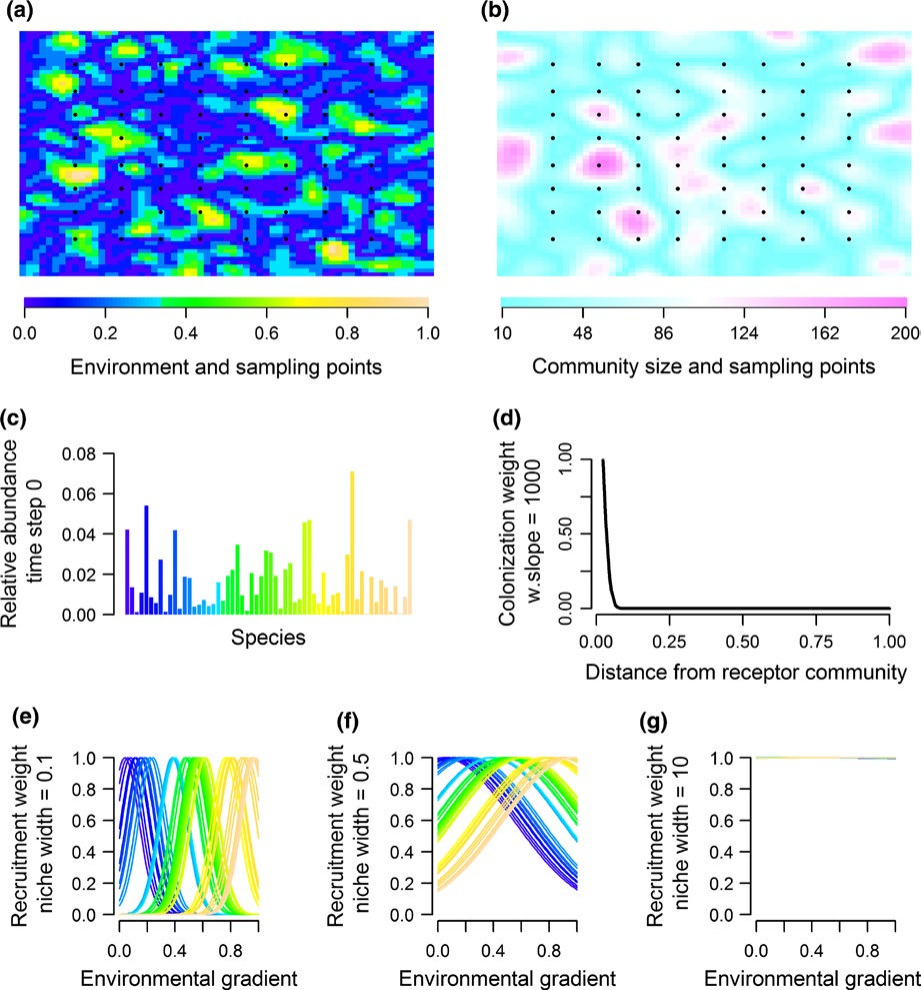
Landscape and parameter settings used in the metacommunity simulations. All simulations were conducted on a landscape consisting of (a) an environmental gradient and (b) varying community sizes. Each raster pixel in (a) and (b) contained a local community. Black dots in (a) and (b) show the location of the local communities that were used in the subsequent analyses. The relative abundance of species in the regional species pool differed at the onset of the metacommunity simulations (time step 0), emulating a typical species pool with few common and many rare species. (d) In scenarios where species were dispersal limited, the colonization weight of species decreased with distance to the receptor community. (e) In scenarios where community assembly was subjected to strong environmental filtering, the niche width of species prevented them from being recruited into local communities with unsuitable environmental conditions. (f) Under intermediate environmental filtering, species were allowed to establish within habitats with suboptimal environmental conditions, but had lower recruitment probabilities in these habitats. (g) When species were neutral, recruitment probabilities were arbitrary with respect to environmental conditions. Figure layout inspired by Sokol et al. (2017)

We generated “empirical” metacommunities through simulations, where community assembly followed 15 different scenarios differing in terms of the influence of spatially restricted dispersal, immigration rates, strength of environmental filtering, and subsequently stochasticity (Figure 1). Taken together, the 15 scenarios represented a wide range of metacommunity dynamics. The simulated scenarios without spatially restricted dispersal, and with three different levels of immigration rates (low, intermediate, and high), allowed us to test whether UniCAA was able to distinguish between environmentally filtered and stochastically assembled metacommunities. Whereas the scenarios with spatially restricted dispersal and three levels of environmental filtering (strong, intermediate, and not restricted), and three levels of immigration rates (low, intermediate, and high) allowed us to test whether UniCAA was able to identify the role of spatially restricted dispersal in community assembly. To evaluate whether UniCAA consistently identified the processes that had shaped the distribution of species within the metacommunity, we simulated 25 replicates of each of the 15 scenarios.

### 2.5 Metacommunity simulation

Metacommunity simulations were performed on a data‐generated landscape consisting of 3,969 communities where both environmental conditions (Figure 1a) and community sizes (Figure 1b) were spatially correlated (Bivand, Pebesma, & Gomez‐Rubio, 2013; Hijmans et al., 2016; Pebesma & Bivand, 2005). We implemented the spatial correlation by applying a Gaussian filter with sigma values 0.2 for environmental conditions, and 0.4 for community sizes to a raster map with uniformly distributed values. This ensured that environmental gradients were steeper than community size gradients (Figure 1a,b) so that regions with a certain community size could harbor different habitat types (Supporting information Appendix S2). The environmental gradient consisted of two‐digit values between zero and one (Figure 1a). Community sizes varied from 10 to 200, by increments of 10 (Figure 1b). Within this landscape, we placed a grid of 64 evenly spaced sampling locations, thus removing the spatial autocorrelation in environmental conditions (*r *= −0.066) and community sizes (*r *= −0.065) between sampling points.

We adopted the approach of Sokol, Brown, and Barrett (2017) to build a metacommunity simulation program in R (Supporting information Appendix S2) that allowed us to simulate metacommunities that consisted of several local communities assembled with or without spatially restricted dispersal, with varying degrees of habitat connectivity (i.e., immigration rates) and with strong, intermediate, and no environmental filtering. Other spatially implicit simulation approaches allow simulating metacommunity dynamics under environmental filtering, stochastic dynamics, and immigration rates (Munoz et al., 2018). However, a strength of the simulation approach of Sokol et al. (2017) is that it is spatially explicit so that the pool of potential immigrants that can reach a community changes as the metacommunity evolves, that is, the simulated metacommunities never reach a stable equilibrium. We therefore deemed the approach by Sokol et al. (2017) to result in more realistic metacommunities. During the simulation process, the species composition within each community evolved over *n* time steps from its initial state (time step_0_). The evolution of each community was determined by: the local environmental conditions; the size of the community; the geographic position of the community; the immigration rate; whether or not dispersal was spatially restricted; the habitat requirement of each species, that is, its fundamental niche; and the environmental tolerance of each species, that is, its niche width.

The species composition within a community at time step_0_ was determined by calculating the environmentally weighted recruitment probability of each sp species (RP_sp_) following Equations (1) and (2).

(1) $$\text{UW}{\text{.RP}}_{\text{sp}}={\text{RA}}_{\text{sp}}\times {\text{exp}}^{\frac{-{\left(E-\mu_{\text{sp}}\right)}^{2}}{2\times \sigma_{\text{sp}}^{2}}}$$

(2) $${\text{RP}}_{\text{sp}}=\frac{\text{UW}{\text{.RP}}_{\text{sp}}}{\sum \text{UW}{\text{.RP}}_{\text{sp}}}$$

where UW.RP_sp_ was the unweighted recruitment probability of species_sp_. RA_sp_ was the predetermined regional relative abundance for a species. RA_sp_ was defined by randomly selecting 60 numbers (one for each species) of a *β*‐distribution with *α* = 1, and *β* = 10, thus ensuring a typical species abundance distribution with many rare and few common species in the metacommunity (Figure 1c). *E *was the environmental conditions, μ the species‐specific environmental optima, and σ the niche width. ΣUW.RP_sp _was UW.RP_sp_ summed across all species in the community. The species composition within each community was then determined by sampling individuals of each species, with their probability of being sampled weighted according to their community‐specific RP_sp_, until the community was saturated.

During each subsequent time step, the species composition within each community was determined through three steps. First, the relative abundance of each species within an immigration pool was calculated (RAIP_sp_) following Equations (3) to (5):

(3) $${\text{DBW}}_{source\;community}={\text{exp}}^{\left(-w\times r^{2}\right)}$$

(4) $$\text{UW}{\text{.RAIP}}_{\mathrm{sp}}=\sum {\text{RA}}_{\mathrm{spinsourcecommunity}}\times {\text{DBW}}_{\mathrm{sourcecommunity}}$$

(5) $${\text{RAIP}}_{\text{sp}}=\frac{\text{UW}{\text{.RAIP}}_{\text{sp}}}{\sum \text{UW}{\text{.RAIP}}_{\text{sp}}}$$

where DBW_source community_ was the predetermined distance based weight (*w.slope*) with which to weight potential immigrants from source communities according to their geographic distance (*r*) to the receptor community. Site distances were scaled between zero and one prior to calculating the DBW_source_ _community_. UW.RAIP_sp_ was the unweighted relative abundance of species_sp_ in the immigration pool, RA_sp in source community _was the proportionate abundance of species_sp_ within a potential source community. RAIP_sp_ was the weighted relative abundance of species_sp_ in the immigration pool, and ΣUW.RAIP_sp _was the sum of UW.RAIP_sp_ for all species that might immigrate into the focal community. The relative contribution of the relative abundance of each species within the focal community (RA_focal community_) at the previous time step, and that of the species in the immigration pool was weighted according to the immigration rate.

(6) $${\text{RI}}_{\text{sp}}={\text{RAIP}}_{\text{sp}}\times \text{immigration rate}+RA_{\text{focal community}}\times (1-\text{immigration rate)}$$

Where immigration rate was the predetermined weight assigned to the immigration pool, relative to the relative abundance of species within the focal community during the previous time step. Lastly, the species composition within the community at time *t* was determined following the same random selection procedure as when determining the species composition at time step_0_ (Equations 7–8). We reiterated the entire process from Equations ((1), (2), (3), (4), (5), (6), (7), (8))(1), (2), (3), (4), (5), (6), (7), (8)((1), (2), (3), (4), (5), (6), (7), (8)) through *t* time steps.

(7) $$\text{UW}{\text{.RP}}_{\text{sp}}={\text{RI}}_{\text{sp}}\times {\text{exp}}^{\frac{-{\left(E-\mu_{\text{sp}}\right)}^{2}}{2\times \sigma_{\text{sp}}^{2}}}$$

(8) $${\text{RP}}_{\text{sp}}=\frac{\text{UW}{\text{.RP}}_{\text{sp}}}{\sum \text{UW}{\text{.RP}}_{\text{sp}}}$$

### 2.6 Data simulations

We simulated spatially restricted dispersal by weighing the recruitment probabilities of species into the immigration pool, based on the distance between the receptor and source community (Figure 1d). Under spatially unrestricted dispersal, species received the same weight regardless of the distance they would have to travel to enter a community (Sokol et al., 2017). The fundamental niche optima of species was defined by first allocating 20 species to each of three groups: those with optima close to the lower (0.12), medium (0.5), or higher (0.88) end of the environmental gradient. We allowed the niche optima of species within each group to evolve following a Brownian motion under 1,000 simulations, but always bound within the initial niche optima ±0.125, to emulate a scenario where traits evolve within functional guilds.

We ran 25 independent metacommunity simulations for each of the 15 metacommunity scenarios resulting in a total of 375 datasets. Each metacommunity scenario was defined by species: having a narrow (0.1), intermediate (0.5), or a wide niche width (10); having spatially restricted dispersal so that the distance based immigration weight (Equation 3) decreased with the distance between sites (w.slope = 1,000) or being allowed to disperse across the entire landscape (w.slope = 0). The immigration rate (Equation 6) was set to three different levels: low (0.25), intermediate (0.5), or high (0.75). In each of the metacommunity simulations, the metacommunity evolved through 50 time steps, enough for the dissimilarity between the resulting metacommunity and the metacommunity at time step_0_ to stabilize.

### 2.7 Data preparations

We sampled 64 evenly spread communities within each of the 375 simulated metacommunities, emulating a scenario where ecologists sample local communities within a wider regional metacommunity (Figure 1a,b). We split the species optima that had been used in the data simulations into a two‐level categorical variable (low, high) to exemplify the typical scenario where functional response traits serve as proxies for species optima. The categorical trait variable was used in the subsequent analyses (hereafter referred to as Trait).

### 2.8 Applying UniCAA to the simulated datasets

#### 2.8.1 Step 1: Identifying the drivers of community assembly

We applied the UniCAA framework to each of the 375 datasets. Because of the large number of models, we did not perform the manual variable selection described above. Instead, we developed an R function that automatically constructed two versions (log‐transforming vs. untransformed Distance to source habitat) of a full model (i.e., including all interaction terms and their main effects) and selected the version with the lowest Bayesian information criterion (BIC) value. We then used the automated model selection function *dredge()* in MuMIn (Barton, 2013) to select the final model with the lowest BIC value. We applied this model selection procedure on each of the 375 datasets, extracted the *z*‐score (effect size) for each parameter estimate from the 25 models per scenario, and calculated the average *z*‐score, its standard deviation, the minimum and maximum *z*‐scores, as well as the number of simulations in which a fixed effect term was included. We used the DHARMa package in R (Hartig, 2018) to validate the final model formulations by visualy inspecing the residual distributions for each of the 375 models. We did not detect any systematic relationships between the standardized residuals and the predicted values of the models, and only in few cases within each of the 15 scenarios did the residual distribution deviate from normality. These cases were mainly restricted to scenarios with either spatially restricted dispersal and/or environmental filtering and low immigration rates. This indicates that, overall, the models were correctly specified (Supporting information Appendix S3).

#### 2.8.2 Step 2: Final model versus null model

We tested if the relationships between species occurrences and spatially restricted dispersal, environmental filtering, and ecological drift that we had observed in step 1, differed from null models in which species distributions were stochastic. We applied step 2 of the UniCAA framework to all 15 scenarios with null models consisting of 99 randomizations as the fn.*UniCAA.sim.eval *function showed the null models saturated at this point.

To test whether the relationship between patterns of species occurrence and community assembly processes differed between the empirical data (in our case, simulated data) and the simulated data (null models with 99 randomizations), we calculated the effect sizes (*z*‐scores) from the interaction terms with Data source. Effect sizes (*z*‐scores) larger than an absolute value of two (1.96) indicated a statistically significant difference. All analyses and data simulations were conducted in R v. 3.5.0 (R core team, 2018), and GLMMs were fitted using the R package lme4 (Bates et al., 2015).

## 3 RESULTS

**Figure 2:**
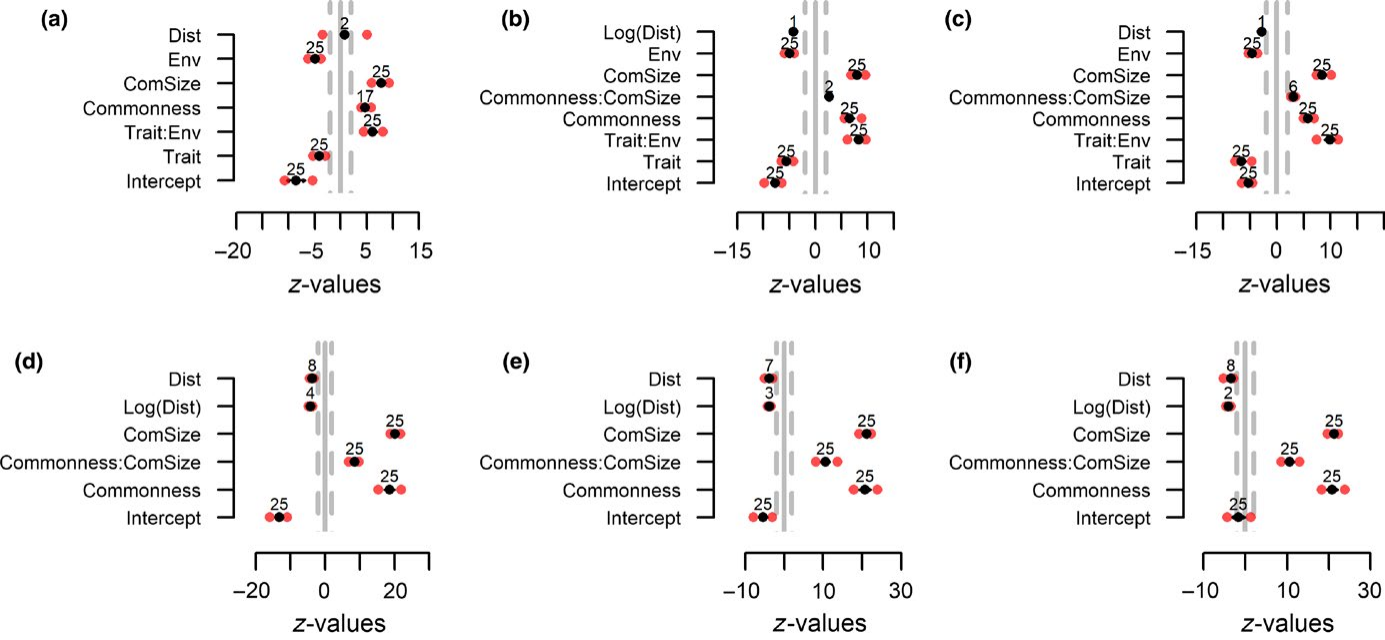
Step 1—without spatially restricted dispersal. Metacommunities were simulated with (a–c) or without (d–f) environmental filtering and with low (left panels), intermediate (middle panels) or high (right panels) immigration rates. Black points show the mean and red points show the minimum and maximum effect size for each explanatory term. Gray dashed lines mark the cutoff value for statistical significance (i.e., an absolute value of two). Numbers above the mean *z*‐values show the number of models (out of 25) in which a term was included. Positive and negative effect sizes indicate if community assembly processes led to an increase or decrease in species occurrence, respectively. For the Community size × Commonness interaction, a positive effect size indicates that the rate of increase in occurrence with community size depends on the commonness of species. For the environmental conditions × functional traits interaction, a positive effect size indicates that species occurrences along the environmental gradient (filter) depend on the functional traits of species

**Figure 3:**
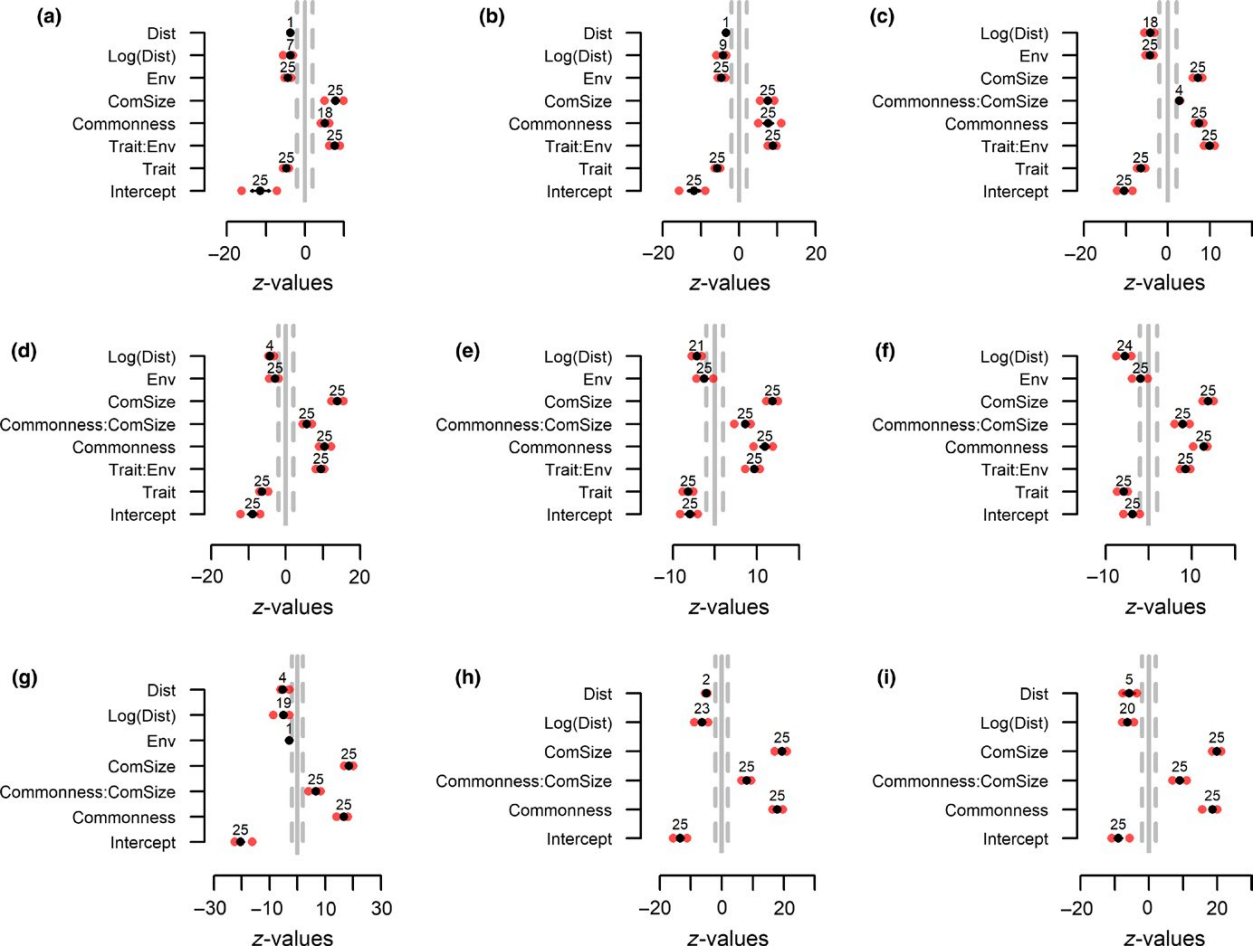
Step 1—with spatially restricted dispersal. Metacommunities were simulated with strong (a–c), intermediate (d–f) or without environmental filtering (g–i) and with low (left panels), intermediate (middle panels) or high (right panels) immigration rates. Black points show the mean and red points show the minimum and maximum effect size for each term. Gray dashed lines mark the cutoff value for statistical significance (i.e., an absolute value of two). Numbers above the mean *z*‐values show the number of models (out of 25) in which a parameter was included. Positive and negative effect sizes indicate if community assembly processes led to an increase or decrease in species occurrence, respectively. For the community size × commonness interaction, a positive effect size indicates that the rate of increase in occurrence with community size depends on the commonness of species. For the environmental conditions × functional traits interaction, a positive effect size indicates that species occurrences along the environmental gradient (filter) depend on the functional traits of species

**Figure 4:**
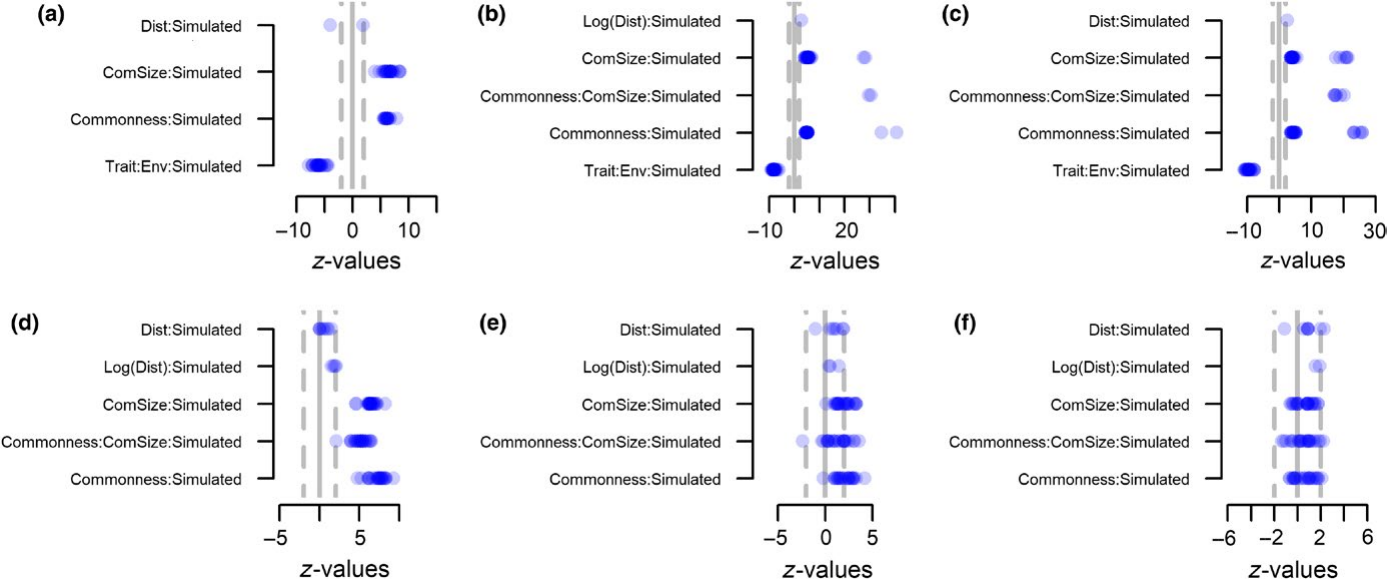
Step 2—without spatially restricted dispersal. Effect sizes (*z*‐values) from step 2 of the differences in regression slopes for community assembly processes between the “empirical” data (i.e., simulated “empirical” datasets) and null models for simulated metacommunities without dispersal limitation. Metacommunities were simulated with (a–c) or without (d–f) environmental filtering and with low (left panels), intermediate (middle panels) or high (right panels) immigration rates. Blue points show the *z*‐value for the interaction terms between the drivers of community assembly, identified in step 1, and the data source (“empirical” vs. null model). Points are shaded as to reflect the density distribution of *z*‐values so that dark blue indicates a high density of models with the corresponding *z*‐value. Positive effect sizes show that the rate of change in species occurrence brought on by a community assembly process was weaker than expected given the null model. Negative effect sizes show the opposite, whereas effect sizes in the interval −2:2 show that the rate of change in species occurrence matches that expected from the null model (i.e., it is neutral)

**Figure 5:**
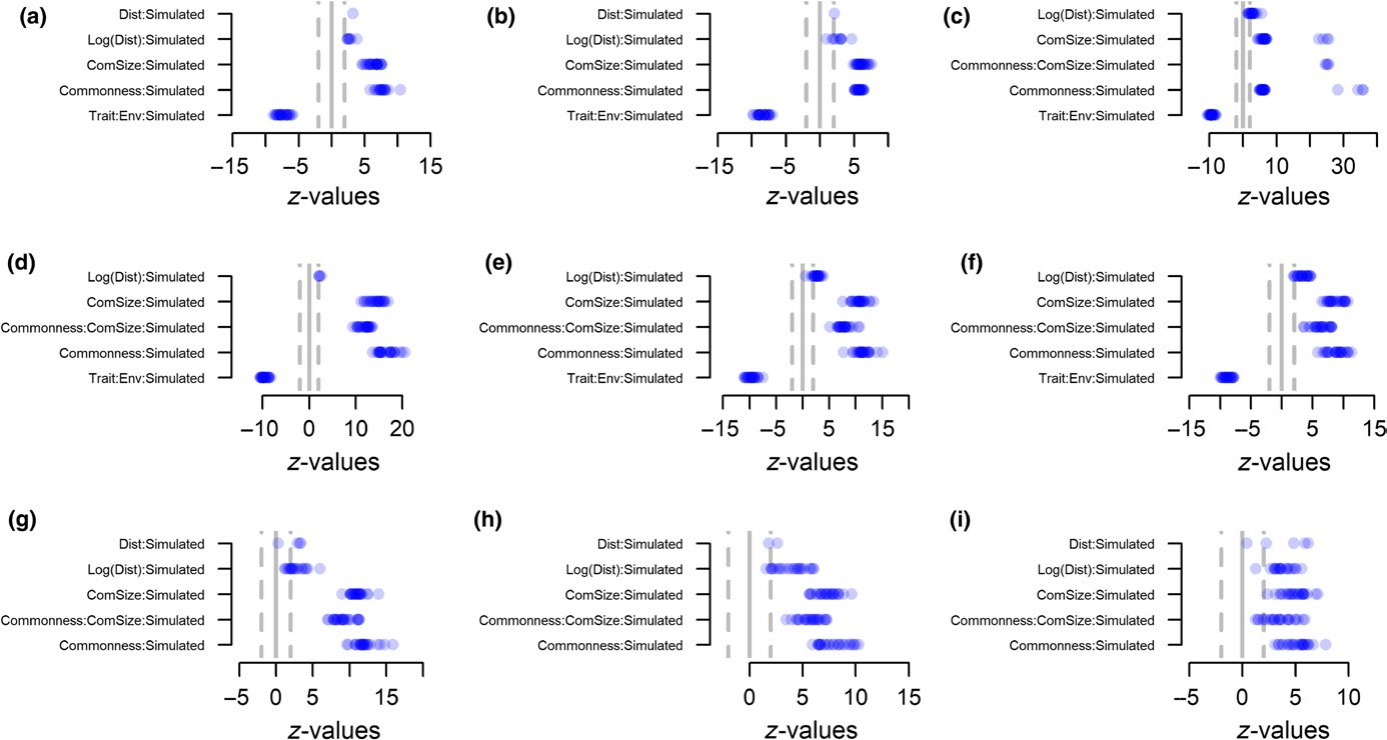
Step 2—with spatially restricted dispersal. Effect sizes (*z*‐values) of the differences in regression slopes for community assembly processes between the “empirical” data (i.e., simulated “empirical” datasets) and null models for simulated metacommunities with dispersal limitation. Metacommunities were simulated with strong (a–c), intermediate (d–f) or without environmental filtering (g–i) and with low (left panels), intermediate (middle panels) or high (right panels) immigration rates. Blue points show the *z*‐value for the interaction terms between the drivers of community assembly, identified in step 1, and the data source (“empirical” vs. null model). Points are shaded as to reflect the density distribution of *z*‐values so that dark blue indicates a high density of models with the corresponding *z*‐value. Positive effect sizes show that the rate of change in species occurrence brought on by a community assembly process was weaker than expected given the null model. Negative effect sizes show the opposite, whereas effect sizes in the interval −2:2 show that the rate of change in species occurrence matches that expected from the null model (i.e., it is neutral).

Using simulated data allowed us to test whether the UniCAA consistently retrieved the processes behind community assembly in metacommunities simulated with varying strengths of spatially restricted dispersal, environmental filtering, and stochasticity (Figure 1). The variables to be included in the second step of the analyses were selected in step 1 (Figures 2 and 3). The parameter estimates for these variables were compared with those obtained through null models in step 2 of the analyses. UniCAA had low type‐I error rates (i.e., <5% false positives, Table 2, Figures 4 and 5) and the Distance to source habitat variable was never significant in more than one replication for a given scenario that was simulated without spatially restricted dispersal (Figures 2 and 4). The Trait × Environmental conditions term was never included in models that were simulated without environmental filtering (Figures 2d–f, 3g–I, 4d–f and 5g–i). Moreover, the Community size × Commonness interaction only differed from the null model in one case when metacommunities were simulated to be stochastic (i.e., without spatially restricted dispersal, high immigration rates, and no environmental filtering). Type‐II error rates were also acceptable [i.e., <20% false negatives, (Johnson, Baary, Ferguson, & Müller, 2015)] as UniCAA only failed to identify the Distance to source habitat variable as significant in two models simulated with high immigration rates and no environmental filtering (Table 2). Moreover, UniCAA did not confound stochastic with deterministic metacommunities as the Community size × Commonness interaction term consistently differed between the null model data and the empirical data when included in scenarios with environmental filtering (Figures 4a–c and 5a–f).

**Table 2:** The number of models (out of 25) in which the relationships between species occurrences and the drivers of community assembly differed between the null model and the empirical models in step 2 of the UniCAA analyses. Results from each of the 15 scenarios are ordered according to whether or not metacommunities were structured by: spatially restricted dispersal; environmental filtering; and low, intermediate, and high immigration rates. The explanatory variables (fixed effect terms) were: the interaction term between the relative abundance of a species outside the focal community (Commonness) and the size of the focal community (Community size); the species‐specific geographic distance from a focal community to the nearest community where the species was found (Distance to source habitat); and the interaction term between the species‐specific functional traits (Trait) and the community‐specific environmental conditions (Environmental conditions). For each fixed effect term, the number of models in which the null model showed a more positive (*z* ≥ 2) or negative (*z* **≤ −**2) relationship with species occurrences than that found in the empirical data is given. Fixed effect terms that were not included in the final models in step 1, and therefore neither in step 2 are marked with n.a. The number of models per scenario that contained each term is shown in Figures 2 and 3

Scenario ‐ step 2	Community size × Commonness		Commonness		Community size		log(Distance to source habitat)		Distance to source habitat		Trait × Environmental conditions	
	*z* > 2	*z* < 2	*z* > 2	*z* < 2	*z* > 2	*z* < 2	*z* > 2	*z* < 2	*z* > 2	*z* < 2	*z* > 2	*z* < 2
No dispersal limitation												
No env. filtering												
Low	25	0	25	0	25	0	1	0	0	0	n.a.	n.a.
Intermediate	9	1	13	0	12	0	0	0	0	0	n.a.	n.a.
High	1	0	1	0	0	0	0	0	1	0	n.a.	n.a.
Strong env. filtering												
Low	n.a.	n.a.	17	0	25	0	n.a.	n.a.	0	1	0	25
Intermediate	2	0	25	0	25	0	1	0	n.a.	n.a.	0	25
High	6	0	25	0	25	0	n.a.	n.a.	1	0	0	25
Dispersal limited												
No env. filtering												
Low	25	0	25	0	25	0	14	0	3	0	n.a.	n.a.
Intermediate	25	0	25	0	25	0	22	0	1	0	n.a.	n.a.
High	21	0	25	0	25	0	19	0	4	0	n.a.	n.a.
Intermediate Env. filtering												
Low	25	0	25	0	25	0	4	0	n.a.	n.a.	0	25
Intermediate	25	0	25	0	25	0	18	0	n.a.	n.a.	0	25
High	25	0	25	0	25	0	23	0	n.a.	*n*.a.	0	25
Strong env. filtering												
Low	n.a.	n.a.	18	0	25	0	7	0	1	0	0	25
Intermediate	n.a.	n.a.	25	0	25	0	7	0	1	0	0	25
High	4	0	25	0	25	0	14	0	n.a.	n.a.	0	25

UniCAA correctly identified the influence of environmental filtering in all scenarios. When metacommunities were simulated with environmental filtering, the interaction term between Trait and Environmental conditions was included in all models (Figures 2a–c and 3a–f) and its influence differed between the empirical and the null models (Figures 4a–c and 5a–f), irrespective of spatially restricted dispersal and immigration rates (Table 2). The Trait × Environmental conditions term was never included in the final models when metacommunities were simulated without niche‐based differences between species.

Spatially restricted dispersal led to decreasing probabilities of species occurrence as the distance to the nearest site containing conspecifics increased (i.e., negative *z*‐values for “Dist” in Figure 3a–i) and its influence depended on both the immigration rate and the presence of environmental filtering. When immigration rates were reduced (low, intermediate), fewer models contained the Distance to source habitat variable with a parameter estimate that differed from the null model (Figures 3g–h and g–h) compared to when immigration rates were high (Figures 3i and 5i). Similarly, under high immigration rates, the number of models containing significant terms for the Distance to source habitat variable were reduced under strong environmental filtering (Figures 3c and 5c) compared to under intermediate (Figures 3f and 5f) or in lieu of environmental filtering (Figures 3i and 5i).

Species occurrences deviated more from purely stochastic distributions when metacommunities had been simulated with reduced immigration rates, spatially restricted dispersal and (or) environmental filtering. When immigration rates were low, the Community size × Commonness term, if included in the model, always differed between the empirical data and the null model. This was also the case for scenarios simulated with either or both of spatially restricted dispersal and environmental filtering except for one scenario (spatially restricted dispersal, high immigration rates, no environmental filtering) where the estimates for Community size × Commonness did not deviate from the null model in four cases. Thus, increasing immigration rates will—in rare instances—generate the same patterns of species occurrences as expected from stochastic community assembly, even if dispersal is spatially restricted, as long as species are neutral with regards to their niches.

## 4 DISCUSSION

Simulating metacommunities with varying strengths of environmental filtering and dispersal limitation allowed us to assess whether UniCAA was able to identify the processes behind community assembly. UniCAA had acceptable type‐I and type‐II error rates when testing for the influence of spatially restricted dispersal, stochasticity, and environmental filtering. UniCAA also captured the interdependencies of the three processes through, for example, reduced influence of spatially restricted dispersal under strong environmental filtering. A major innovation of the UniCAA framework is the use of flexible mixed effect models to test the influence of all three processes simultaneously, by comparing parameter estimates obtained from the empirical data to those obtained from a null model (Table 2, Figures 4 and 5).

Our results show that the UniCAA framework correctly identified stochastic species distributions. Such distributions scale up to neutral metacommunities in which species are neither environmentally filtered nor dispersal limited (Leibold et al., 2004). However, the influence of the Community size × Commonness interaction differed between the “empirical data” and the null models when immigration rates were intermediate and low, because communities became more isolated, and random extinctions thus became less spatially synchronous. This is in line with the concept of homogenizing dispersal whereby high dispersal rates decrease spatial species turnover and lead to the species composition predicted under pure drift (Stegen et al., 2013). The null model approach in UniCAA bears resemblance to the neutral prior implemented in CATS [“Community Assembly through Trait Selection” (Shipley, Vile, & Garnier, 2006)] where the goodness‐of‐fit statistic (*R* ^2^) of an empirical model is compared to that of a prior distribution. Similarly to the null model approach in UniCAA, the CATS approach can be used to assess how much the relationship between local species abundances and their relative abundances in the metacommunity diverges from what would be expected under stochastic community assembly (Shipley, Paine, & Baraloto, 2012). However, the comparisons between the empirical and null models differ. CATS bases this comparison on the variance explained by the empirical versus the prior distribution, whereas UniCAA tests if the relationships (regression slopes) differ. Another important distinction between CATS and UniCAA is that by including information on the spatial location of communities, UniCAA allows estimating the influence of spatially restricted dispersal on local occurrences.

Distinguishing between the influence of environmental filtering and dispersal limitation is often problematic because environmental conditions tend to be spatially correlated between sampled habitats (Gilbert & Lechowicz, 2004; Peres‐Neto & Legendre, 2010). Although our sampling scheme reduced the spatial correlation in environmental conditions between our samples, thus allowing for meaningful parameter estimates, the underlying environmental conditions and community sizes that generated patterns of species distributions were still spatially correlated. While UniCAA—under our simulation settings—did not confound spatially restricted dispersal with environmental filtering, the degree of environmental filtering did influence the influence of spatially restricted dispersal (Table 2). This was expected, since the spatial component of community assembly disappears under strong environmental filtering, because species are unable to disperse across the landscape (Sokol et al., 2017). Using the UniCAA approach, we were able to show how the influence of spatially restricted dispersal becomes more important as the influence of environmental filters decrease and immigration rates increase (Table 2, Figure 5c). Testing the influence of spatially restricted dispersal and environmental filtering is possible using other frameworks (e.g., Ovaskainen et al., 2017). However, existing frameworks do not make it possible to conclude that community assembly is stochastic if neither parameter estimates for spatially restricted dispersal or environmental filtering are significant, since one may not have included all relevant traits or environmental gradients (Vellend et al., 2014). The null model approach in UniCAA allows testing if patterns of species occurrences differ from what would be expected under purely stochastic community assembly. Environmental filtering will for instance lead to species aggregating within sites with suitable environmental conditions (Kraft et al., 2015). Species may therefore have locally large populations, and a relatively high relative commonness, despite having restricted distributions. Under such scenarios, the mean probability of occurrence will be lower than expected from the regional Community size × Commonness under the null model setting (i.e., positive *z*‐values in Table 2).

In our data simulations and analyses, all species had similar dispersal capabilities. When testing the role of spatially restricted dispersal, that is, Distance to source habitat, it is possible to test whether dispersal abilities differ among trait groups by adding an interaction term between the Distance to source habitat variable and that trait (Sydenham et al., 2017). If the slopes for Distance to source habitat differ significantly between trait groups, this would indicate that the trait modifies dispersal limitation. This may be important, as dispersal capabilities are unlikely to be neutral with regard to species identities and functional traits (Lowe & McPeek, 2014). Another approach is to use β‐diversity indices—calculated for each trait group—to test for differences in dispersal limitation between trait groups (Anderson et al., 2011). Alternatively, one could compare differences in the relative importance of geographic distance versus environmental drivers on species turnover (König, Weigelt, & Kreft, 2017). However, for continuous traits—such as body size—this introduces some subjectivity as to how to classify each trait group. In contrast, it is possible to include continuous traits directly in the UniCAA framework. The flexibility of the UniCAA approach also allows accounting for environmentally defined dispersal distances between sites. Since there were no large barriers to dispersal, species in our simulations were assumed to be able to disperse through all habitat types. Users of the UniCAA should consider, whether the shortest geographic distance between sites accurately reflects the shortest migratory path between communities (Graf, Schadt, Fernández, & Grimm, 2007). If for instance large water bodies separate terrestrial communities, then the shortest migratory path may follow the coastline. In such cases, the species‐specific inter‐site distances used when producing the UniCAA data frame should account for this by, for example, using the gridDistance function in the Raster package in R (Hijmans et al., 2016). Moreover, ecological surveys/datasets are unlikely to include all potential source habitats from which species can immigrate. The distance to source habitat may therefore produce slightly biased estimates. However, because UniCAA estimates the average decrease in occurrence with distance to source habitat, the influence of such outliers is likely to be reduced as more species and sites are included in the analyses. Additionally, the spatial configuration of study sites should be designed so that the spatial correlation in environmental conditions between sites is reduced (Gilbert & Lechowicz, 2004).

UniCAA incorporates the approach of Jamil et al. (2013), that is, tests the influence of environmental filtering through Trait × Environmental conditions terms. When formulating the models, Jamil et al. (2013) included species‐specific random slopes for the environmental gradients. We did not include these in our analyses because including the unimodal responses of species along the environmental gradients as random slopes (i.e., second‐order polynomials for the environmental conditions) led to highly biased parameter estimates of the Trait × Environmental conditions term. However, if species are expected to show linear responses to an environmental gradient, and to differ in these responses, a model that includes random slopes should be compared to one that does not. Despite leaving out random slopes, our simulation study shows that the Trait × Environmental conditions term had acceptable Type‐I, and Type‐II errors; UniCAA always identified scenarios with environmental filtering (Table 2). A strength of incorporating the approach of Jamil et al. (2013) is that it allows testing multiple Trait × Environmental conditions terms simultaneously, thereby allowing for comparisons of their conditional effect sizes. This is particularly important since multiple environmental filters often influence community assembly (de Bello et al., 2013).

Applying UniCAA requires specific hypotheses about trait–environment relationships in order to test the influence of environmental filtering, and therefore a set of functional traits for the species being studied. Whereas traits for some taxa have been compiled in databases (e.g., Homburg, Homburg, Scäfer, Schuldt, & Assman, 2014)—or can be extracted from natural history books—identifying relevant traits requires a careful consideration of traits and environmental gradients (Petchey & Gaston, 2006). If trait data are not available—or if the aim is not to test how environmental filters select for species based on specific traits—then other approaches than UniCAA, such as variation partitioning (Borcard, Legendre, & Drapeau, 1992; Peres‐Neto et al., 2006) will be more appropriate. Variation partitioning identifies the fractions of variation in species composition among communities that is attributable to environmental conditions, geographic distances, the combination of the two, and the unexplained variation. Yet, whereas variation partitioning can be a more flexible approach than UniCAA, an important assumption is that all relevant environmental gradients (i.e., filters) have been measured. If not, one cannot conclude that the variation in species composition associated with spatial distances is not due to environmental filtering (Vellend et al., 2014).

We believe that UniCAA has the potential to become a widely applicable framework, but we also recognize potential limitations and avenues for further development of the framework. Proxies for Distance to source habitat, Community size, and Commonness can be difficult to obtain for un‐surveyed areas. UniCAA does therefore not replace the need for models that provide quantitative predictions of biodiversity in un‐surveyed areas and how this biodiversity may change according to environmental perturbations (D'Amen, Rahbek, Zimmermann, & Guisan, 2017). In its current form, UniCAA should therefore be viewed as a framework for testing hypotheses related to how dispersal limitation, abiotic ecological filtering, and ecological drift influence species occurrences within surveyed communities. Since environmental filtering can influence species abundances (Shipley et al., 2006), future studies should aim to expand the framework to model species abundances and test the applicability of the framework when using such models. When extending the UniCAA to model abundances, one should consider the spatial grain of the sampling units, particularly if biotic interactions are likely to be a central driver of differences in species abundances between sampling units. Not accounting for biotic interactions may be problematic if communities are sampled at spatial grains where competition or facilitation is important, such as at the habitat resource scale. Incorporating formal tests of the presence and strength of biotic interactions would be a significant contribution to the UniCAA framework and is currently a central theme in community and macro‐ecology (D'Amen, Mod, Gotelli, & Guisan, 2018; Morales‐Castilla, Matias, Gravel, & Araújo, 2015; Staniczenko, Sivasubramaniam, Suttle, & Pearson, 2017). A potentially promising avenue is to use the residual correlation matrix between species (random effects) to identify species‐pairs that co‐occur less or more frequently than expected by chance (D'Amen et al., 2018; Warton et al., 2015), after having controlled for the influence of spatially restricted dispersal, environmental filtering, and stochasticity. Unfortunately, our metacommunity simulator did not allow us to incorporate the influence of, for example, competition on community assembly. If competition reduces the number of ecologically similar species that occur within communities, the Trait × Environment interaction terms should still allow identifying if community assembly is environmentally filtered. In such cases, the influence of competition should simply reduce the mean probability of occurrence and abundance of species within trait groups. However, the probability of occurrence should still be greater in habitats with suitable environmental conditions than in habitats with non‐suitable environmental conditions.

## 5 CONCLUSIONS

The UniCAA framework can be used to answer fundamental questions in ecology and enables exploration of novel questions. For instance, since the influence of ecological drift is estimated through the influence of Commonness—which may be determined by speciation and large‐scale dispersal (Cornell & Harrison, 2014)—UniCAA enables us to study how processes that shape the regional species pool in turn influence community assembly. The framework can also be used to identify at which spatial scale (grain size) stochastic species distributions emerges as a consequence of non‐deterministic community assembly processes. Future developments of the framework should focus on implementing the influence of biotic interactions, and also on developing null models for stratifying randomizations within functional groups, as this may allow testing if species with similar traits show stochastic species distributions. Moreover, future studies should aim to compare the outputs of UniCAA to those of other approaches aimed at disentangling the influence of community assembly processes. To accommodate the use of UniCAA, as well as future improvements, we have included two R scripts to allow readers to directly apply and further develop the framework (Supporting information Appendixes [Link], [Link]).

## AUTHOR CONTRIBUTIONS

MAKS conceived the ideas, prepared and analyzed the data, and wrote the first draft of the manuscript; All authors contributed critically with interpretations of the results and revisions of the manuscript.

## Data Availability

This study was based on simulated data. R codes for generating the simulated data and reproducing the results are included in the Supporting information accompanying the paper (Supporting information Appendix S2).
