# Supplementary material for: Univariate Community Assembly Analysis (UniCAA): Combining hierarchical models with null models to test the influence of spatially restricted dispersal, environmental filtering, and stochasticity on community assembly
Source: Ecol Evol. 2019 Jan 13;9(3):1473–88. doi: 10.1002/ece3.4868 (PMC6374725; doi:10.1002/ece3.4868)
Supplement: Supplementary file 3 [file ECE3-9-1473-s003.docx]

Appendix S3 – Residual plots from the 375 final models in step 1

Univariate Community Assembly Analysis (UniCAA): Combining hierarchical models with null models to test the influence of spatially restricted dispersal, environmental filtering and stochasticity on community assembly

Sydenham, M.A.K., Moe, S.R., Steinert, M. and Eldegard, K.

*Faculty of Environmental Sciences and Natural Resource Management, Norwegian University of Life Sciences, NO-1432-Ås, Norway.*

The following pages show residual plots for the 375 models that were fit during step 1 of the UniCAA analyses. The UniCAA framework was tested using simulated metacommunities under 15 different metacommunity dynamic scenarios defined by the relative strengths of spatially restricted dispersal, environmental filtering and immigration rates. To allow evaluating the stability of the UniCAA, 25 replicates were generated for each scenario and analysed separately, resulting in 375 models. We used the *simulateResiduals* function in the DHARMa (Hartig 2018) R package to produce the residual plots. The *simulateResiduals* takes a model object and simulates the distribution of fitted values, for each data point, one would expect if the model was correctly specified. For each data point, the scaled residual corresponds to the proportion of simulated values that are equal to or lower than the observed (predicted) value. A scaled residual of 0.25 thereby corresponds to 75% of the simulated values being larger than the predicted value. Because the accuracy of the residual estimation depends on the number of simulations, we simulated all residuals with 10,000 simulations.

Contents

[Without dispersal limitation, without environmental filtering, low immigration rates 3](#_Toc526506515)

[Without dispersal limitation, without environmental filtering, intermediate immigration rates 29](#_Toc526506516)

[Without dispersal limitation, without environmental filtering, high immigration rates 55](#_Toc526506517)

[Without dispersal limitation, with strong environmental filtering, low immigration rates 81](#_Toc526506518)

[Without dispersal limitation, with strong environmental filtering, intermediate immigration rates 107](#_Toc526506519)

[Without dispersal limitation, with strong environmental filtering, high immigration rates 133](#_Toc526506520)

[With dispersal limitation, without environmental filtering, low immigration rates 159](#_Toc526506521)

[With dispersal limitation, without environmental filtering, intermediate immigration rates 185](#_Toc526506522)

[With dispersal limitation, without environmental filtering, high immigration rates 211](#_Toc526506523)

[With dispersal limitation, with intermediate environmental filtering, low immigration rates 237](#_Toc526506524)

[With dispersal limitation, with intermediate environmental filtering, intermediate immigration rates 263](#_Toc526506525)

[With dispersal limitation, with intermediate environmental filtering, high immigration rates 289](#_Toc526506526)

[With dispersal limitation, with strong environmental filtering, low immigration rates 315](#_Toc526506527)

[With dispersal limitation, with strong environmental filtering, intermediate immigration rates 341](#_Toc526506528)

[With dispersal limitation, with strong environmental filtering, high immigration rates 367](#_Toc526506529)

Hartig, F. 2018. DHARMa: Residual Diagnostics for Hierarchical (Multi-Level / Mixed) Regression Models. R package version 0.2.0. http://florianhartig.github.io/DHARMa/

# Without dispersal limitation, without environmental filtering, low immigration rates


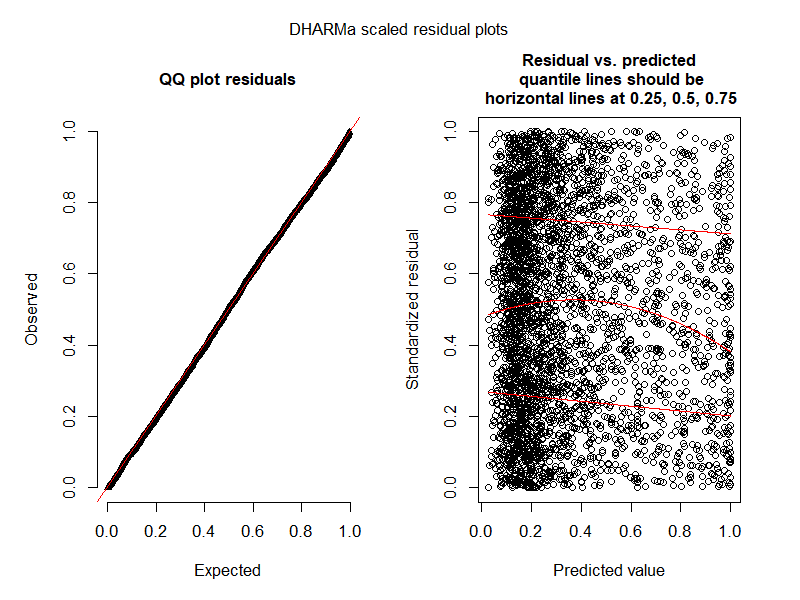

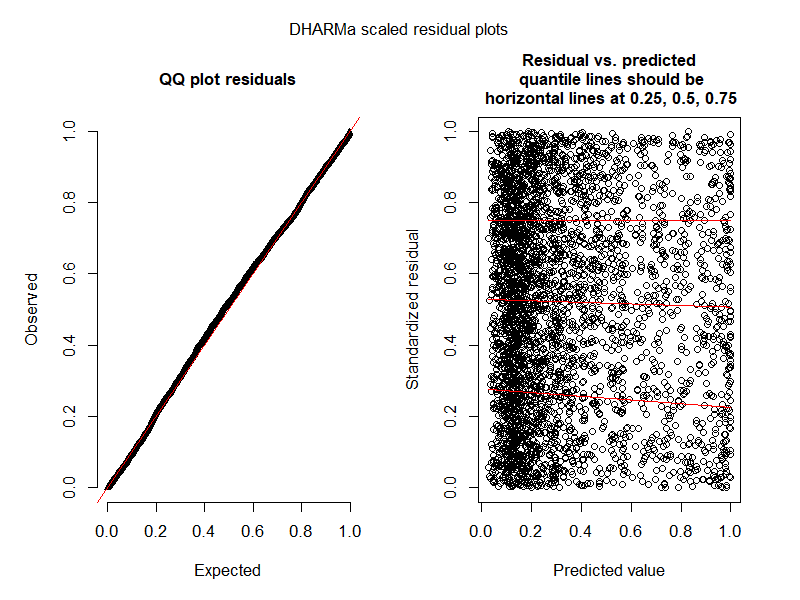

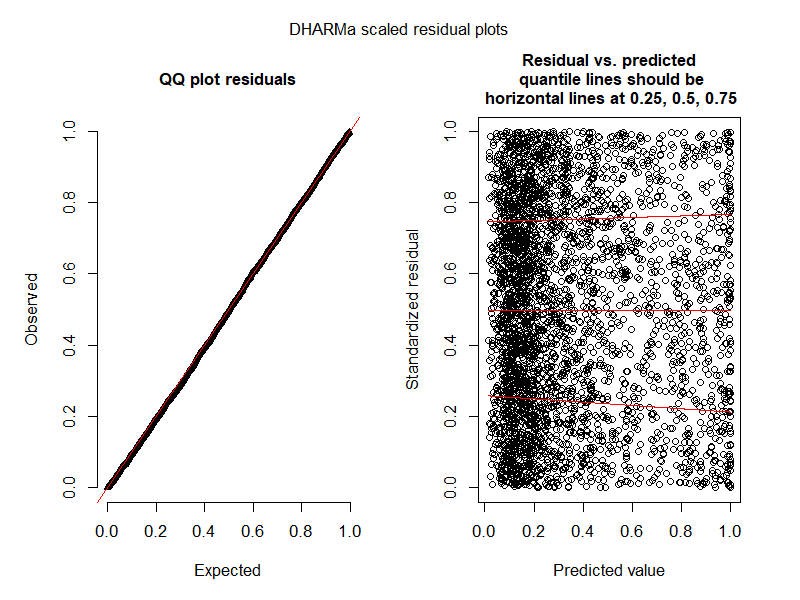

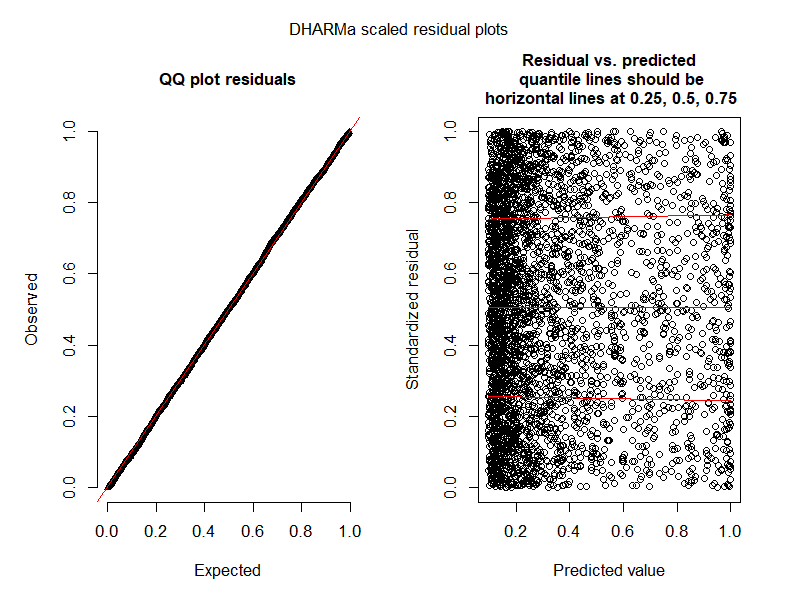

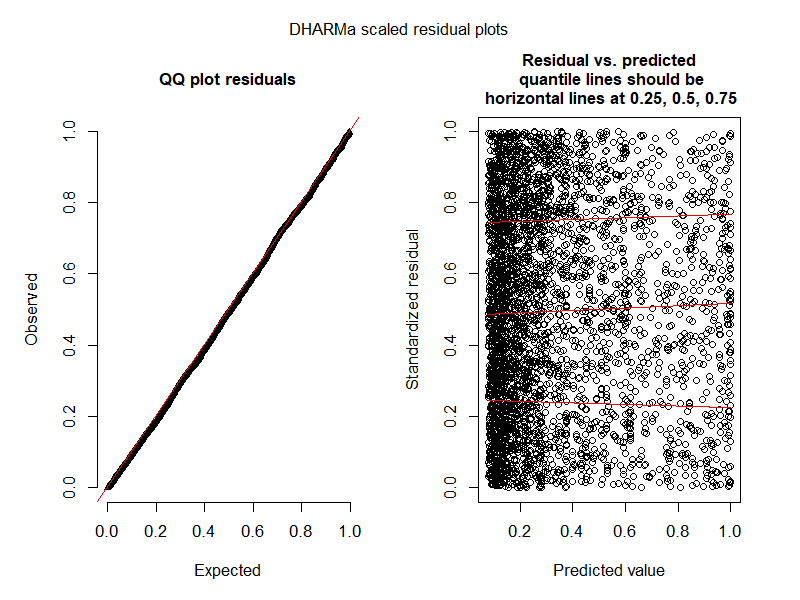

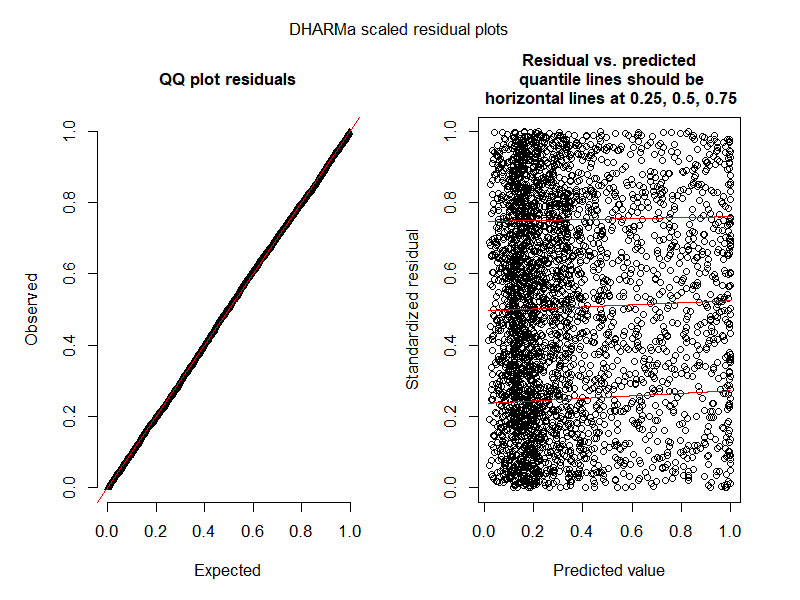

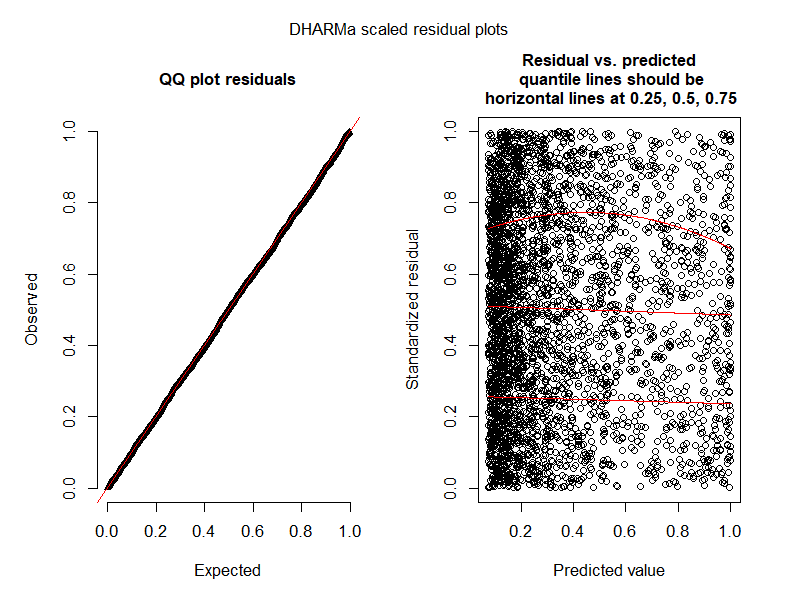

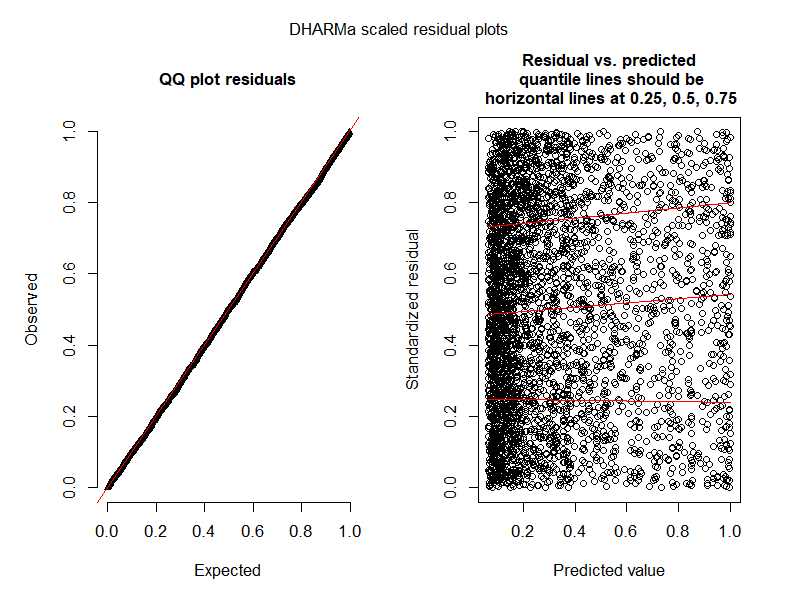

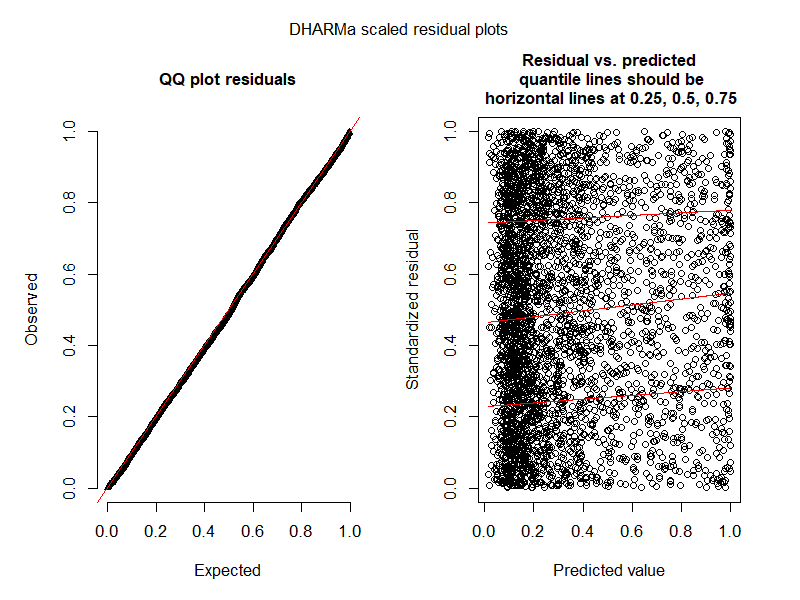

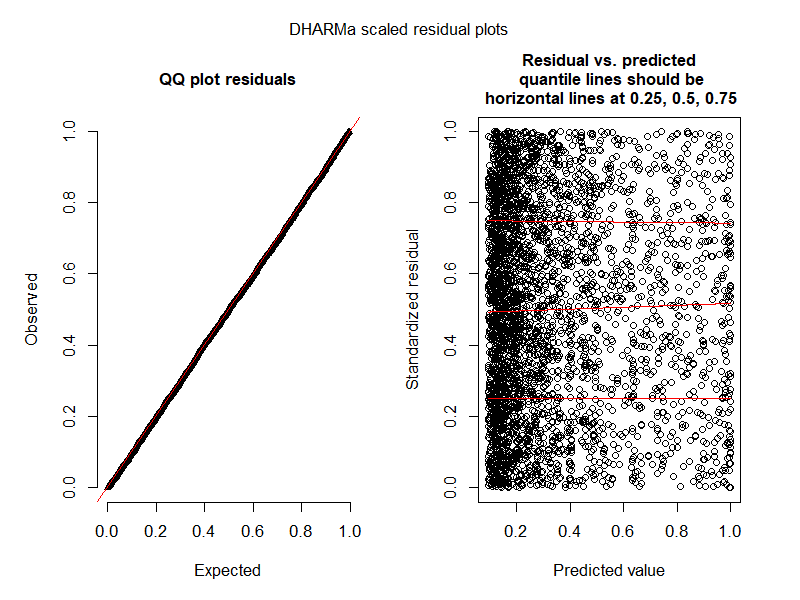

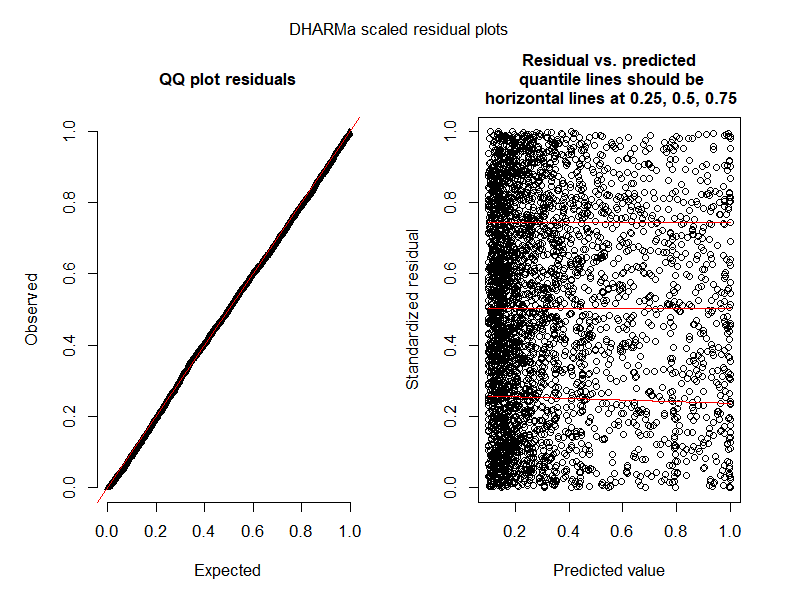

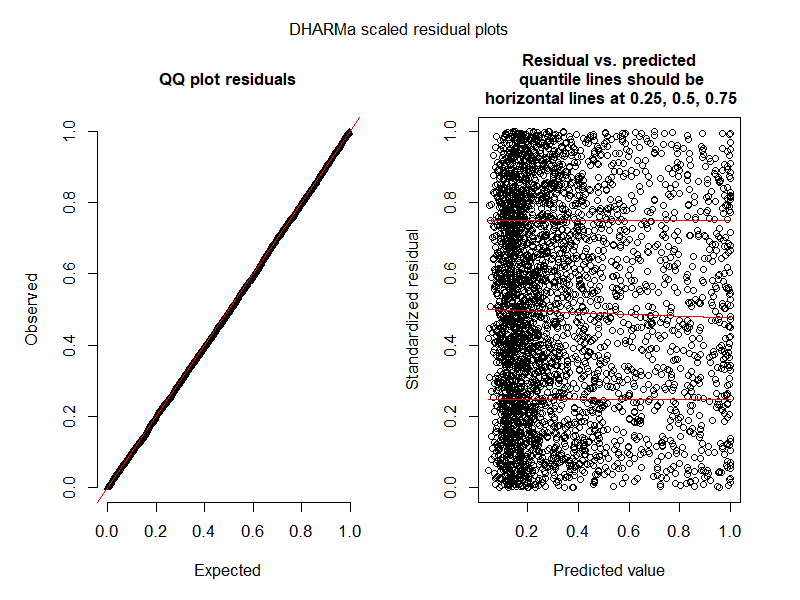

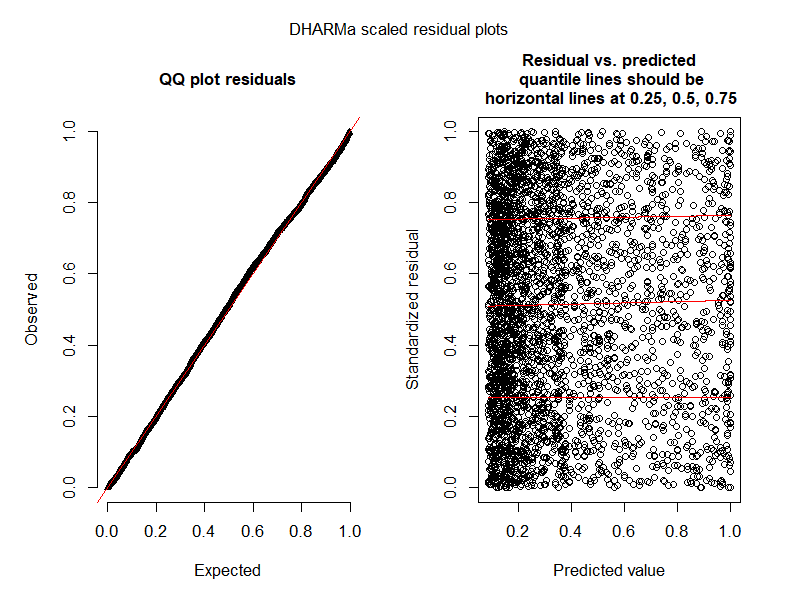

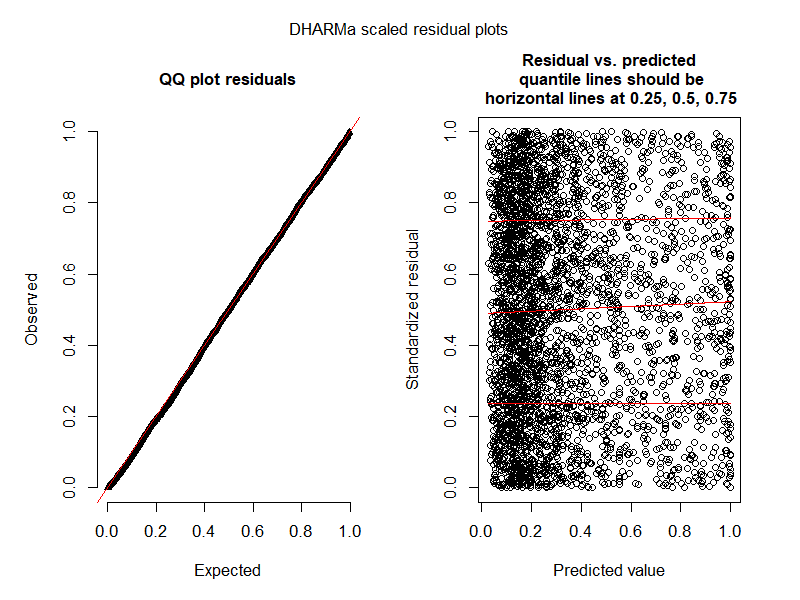

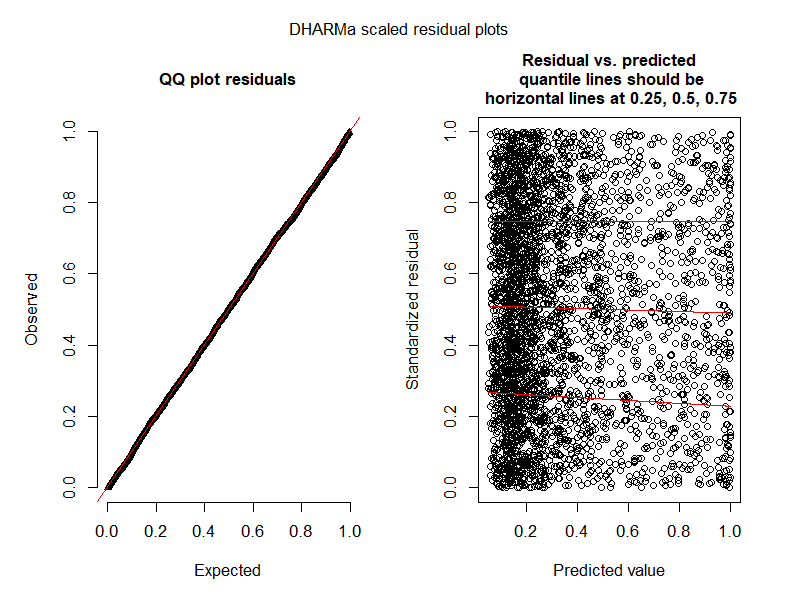

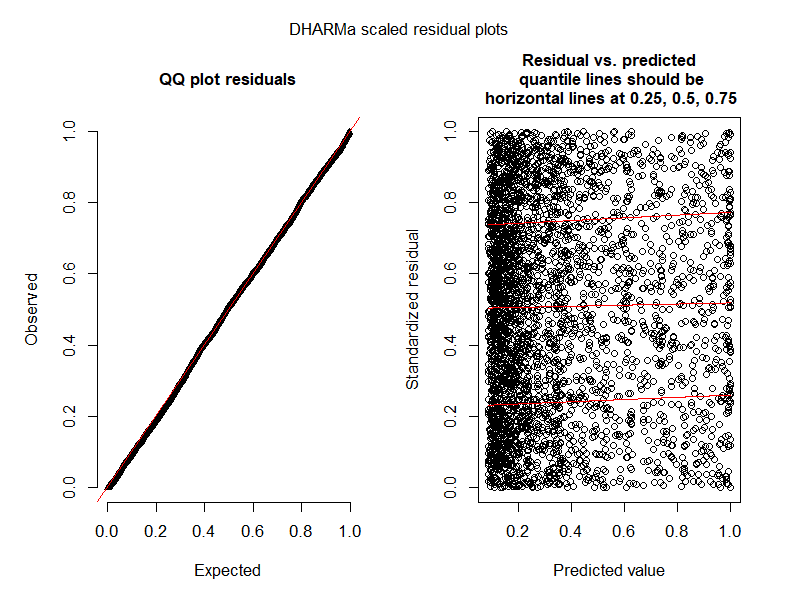

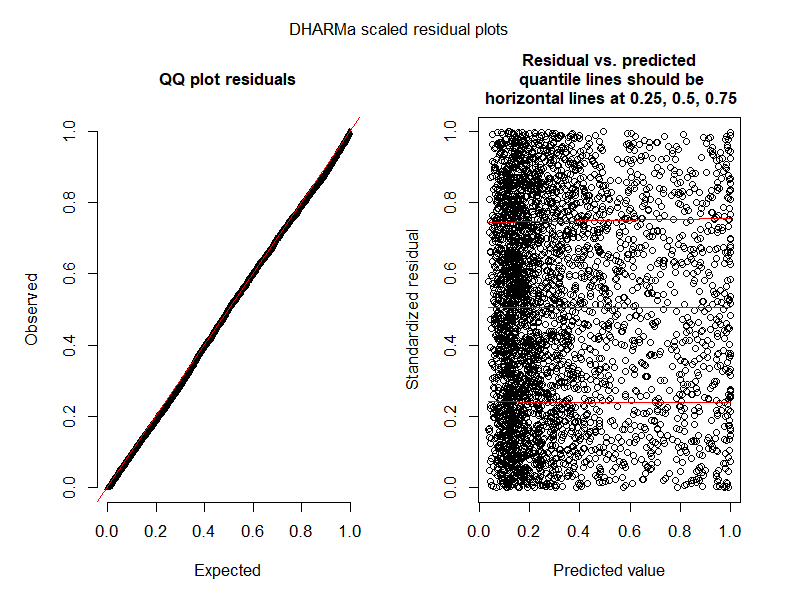

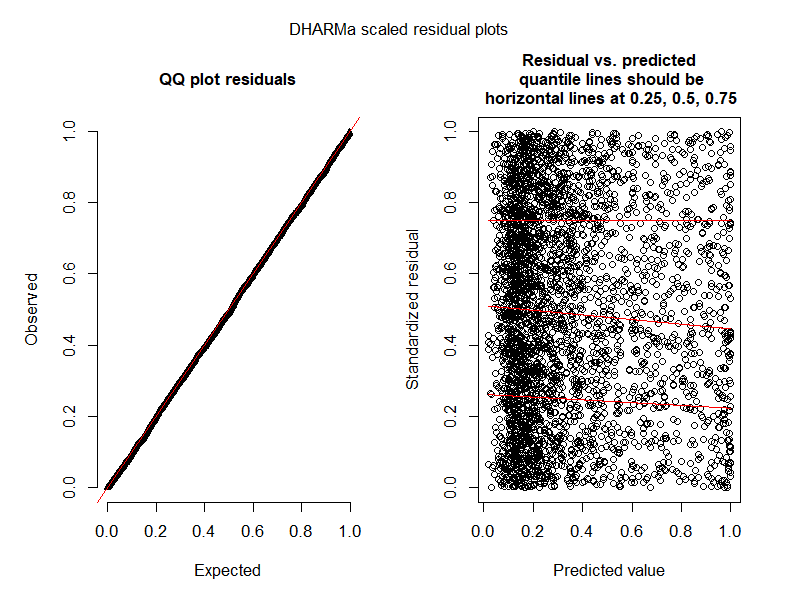

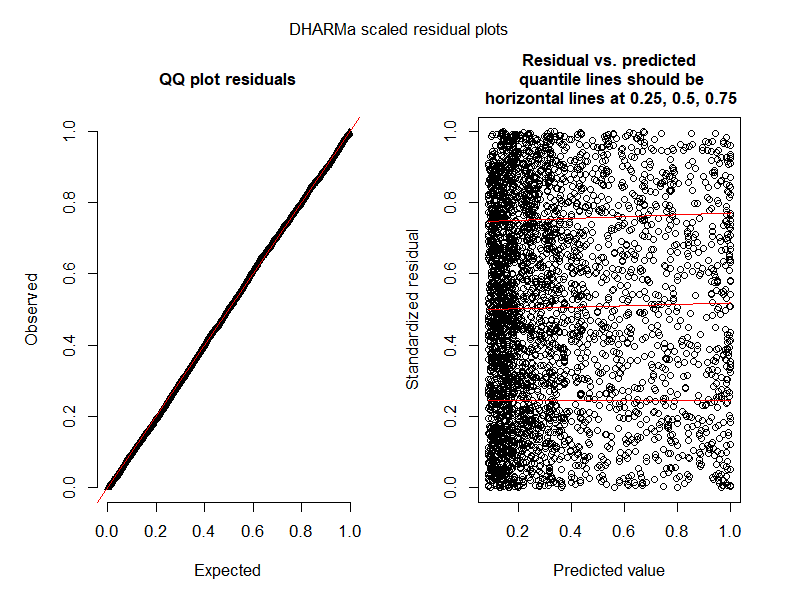

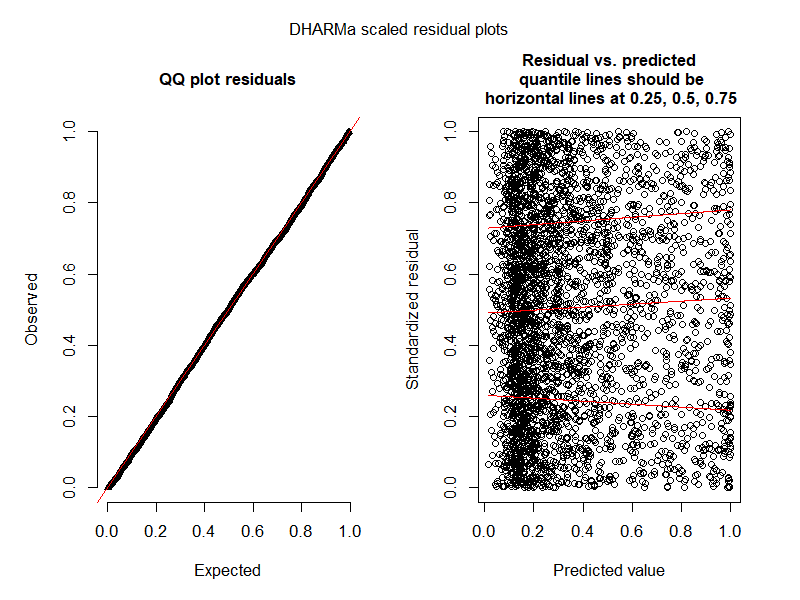

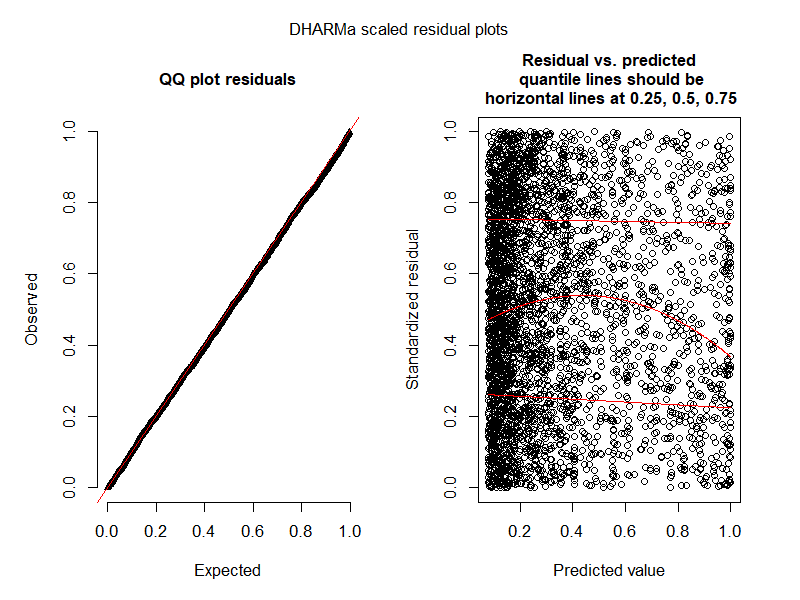

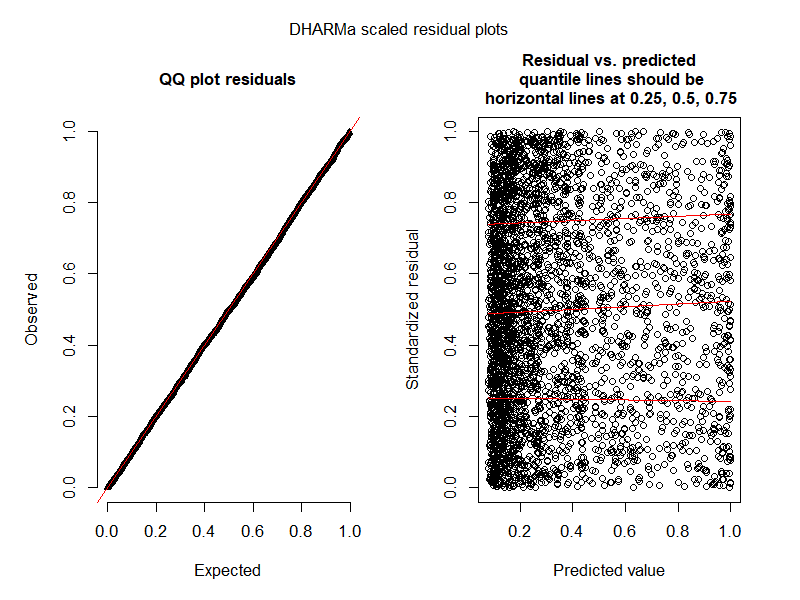

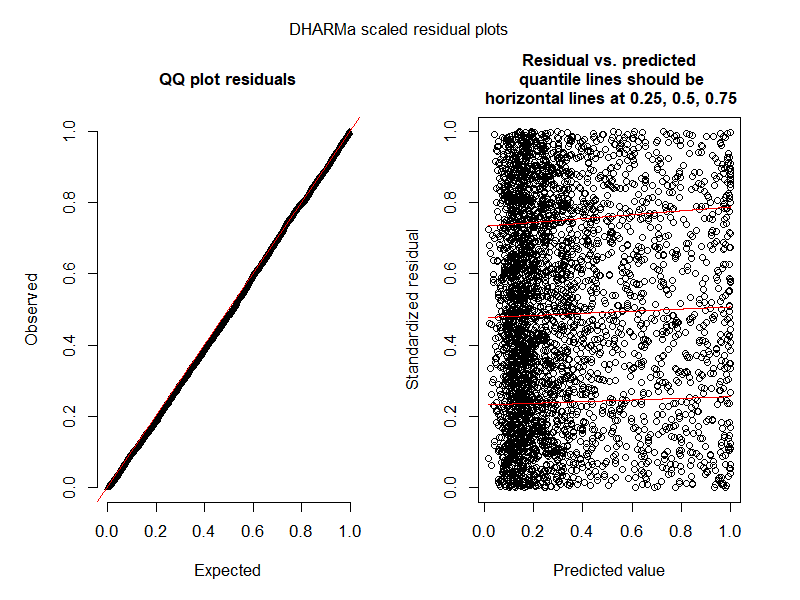

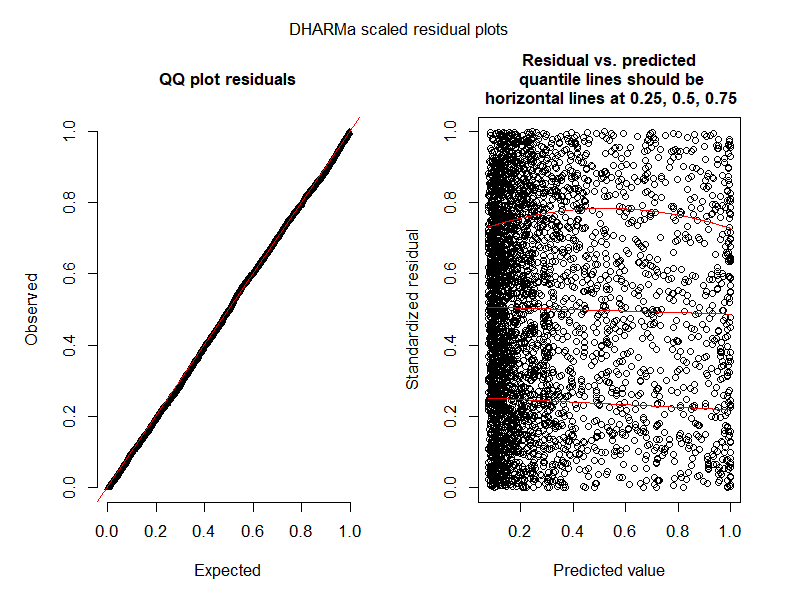

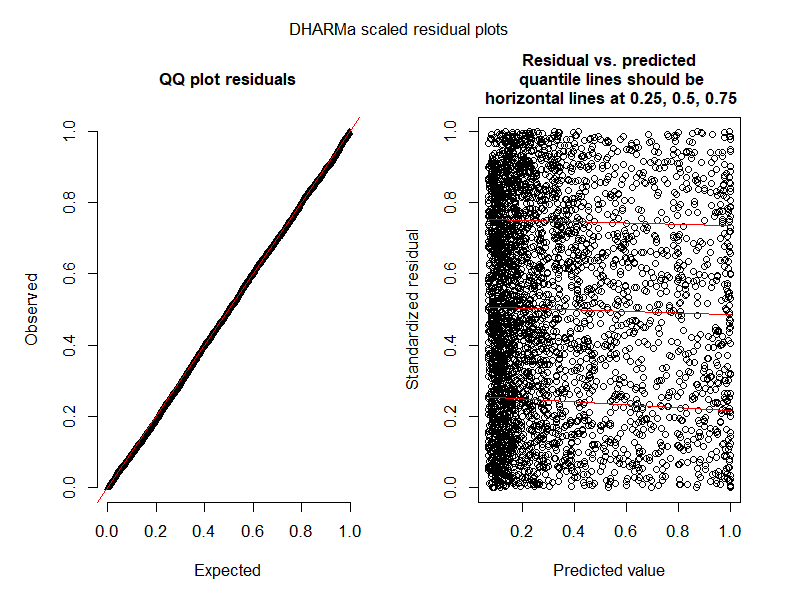


# Without dispersal limitation, without environmental filtering, intermediate immigration rates


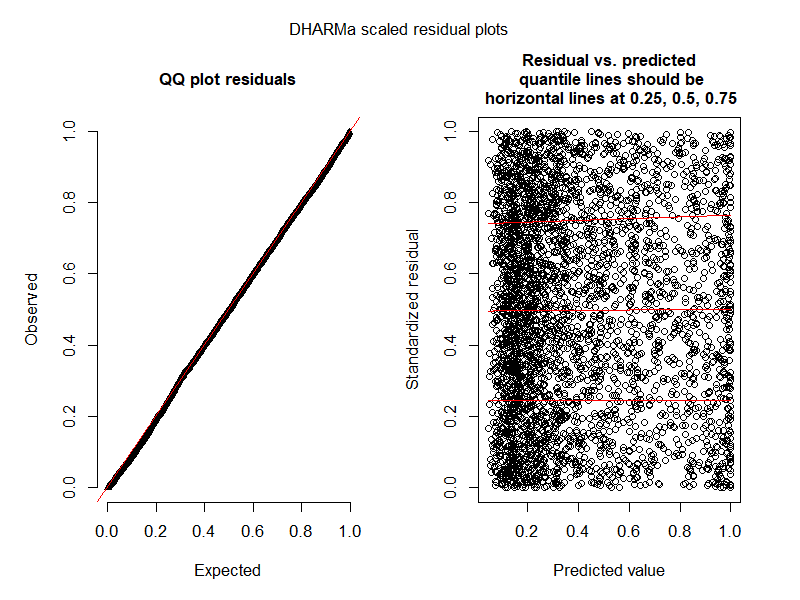

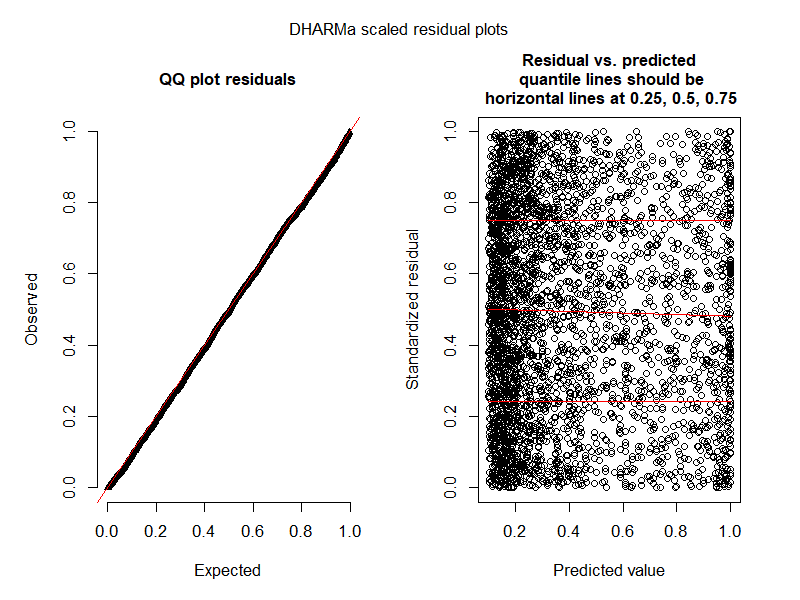

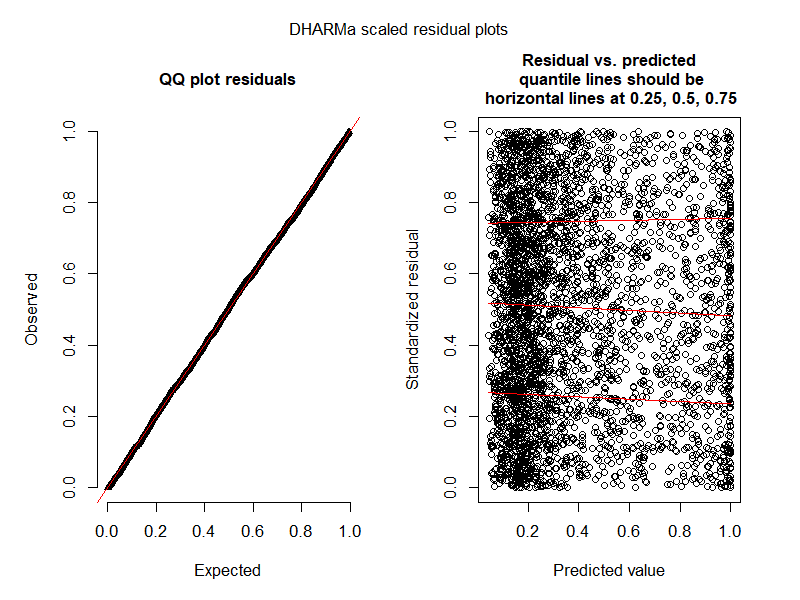

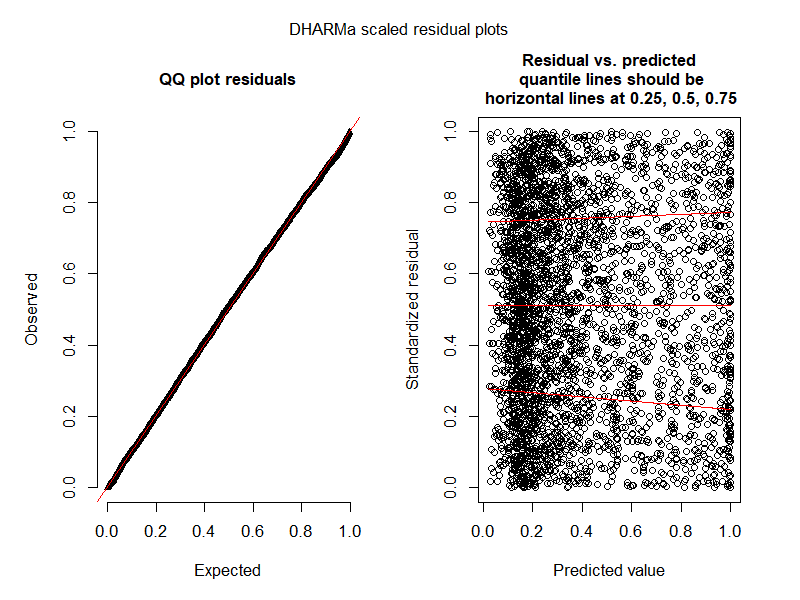

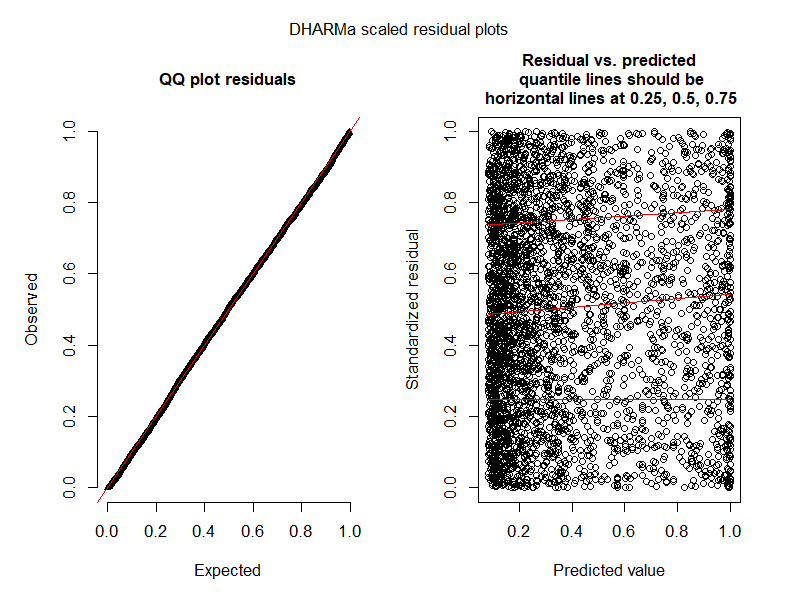

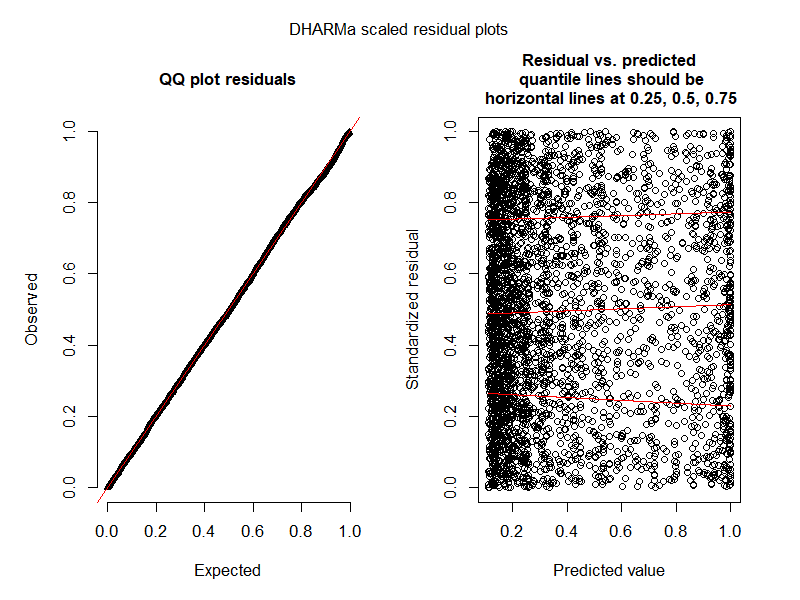

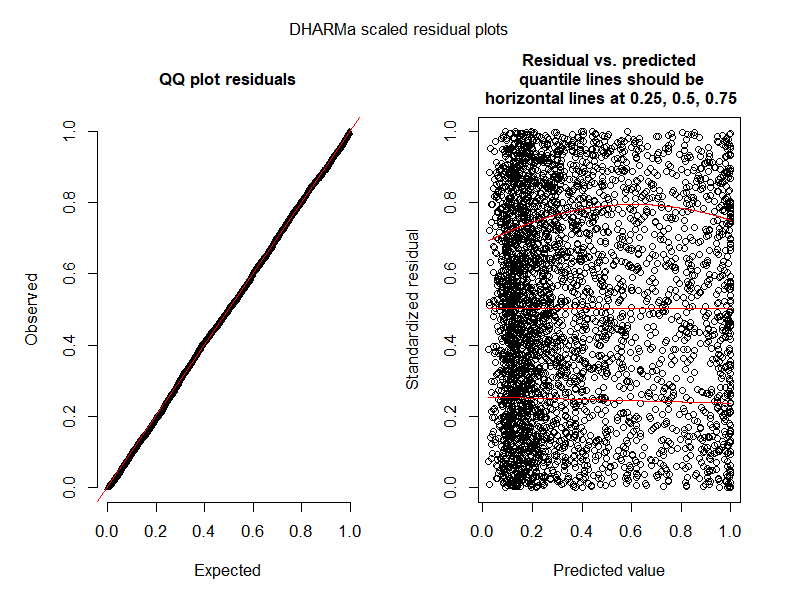

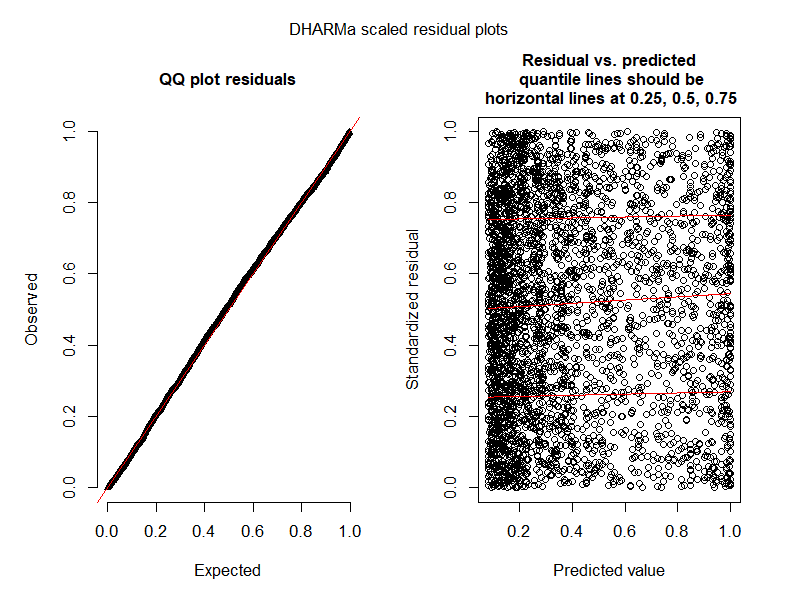

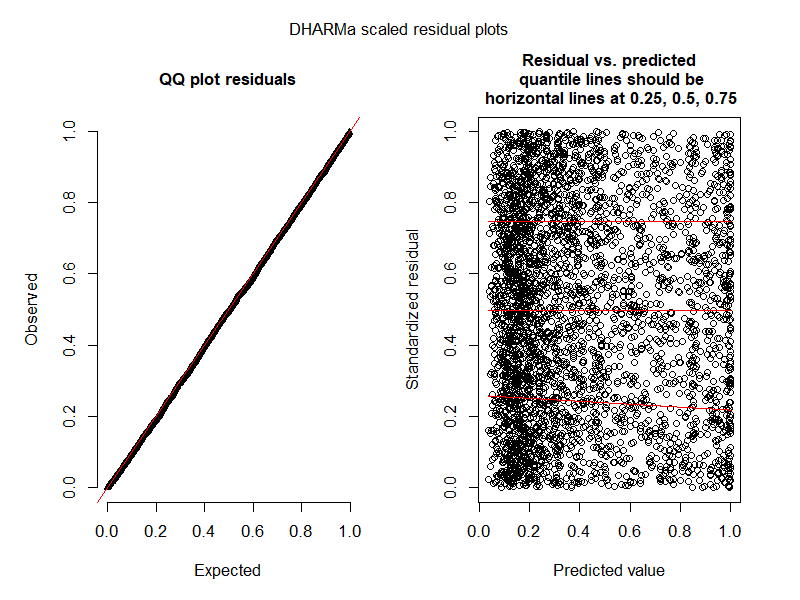

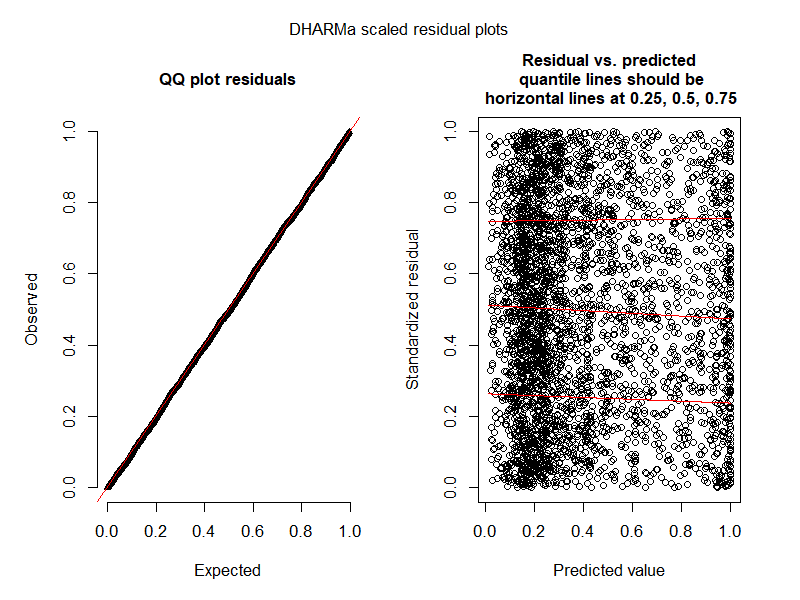

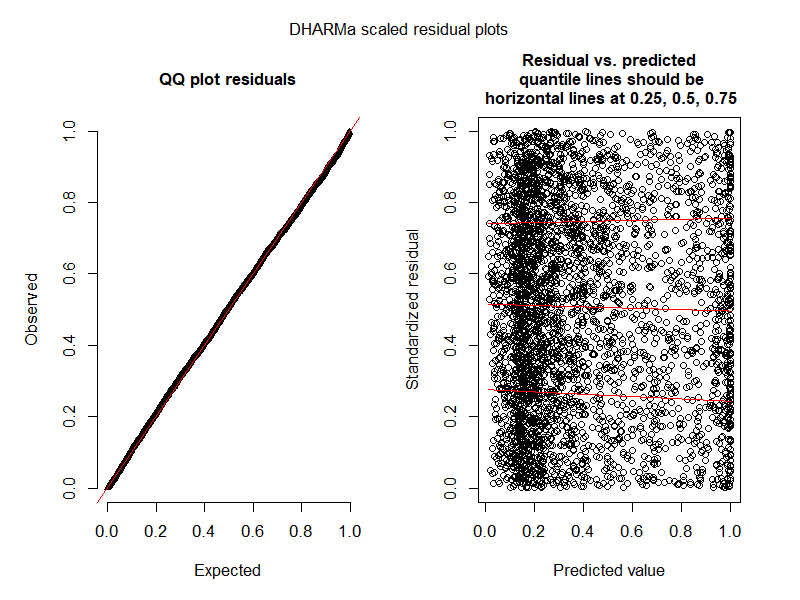

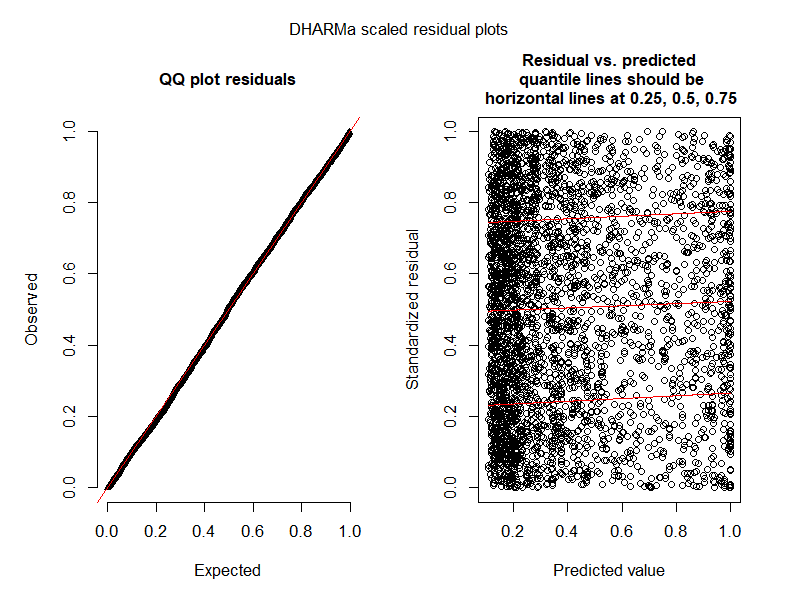

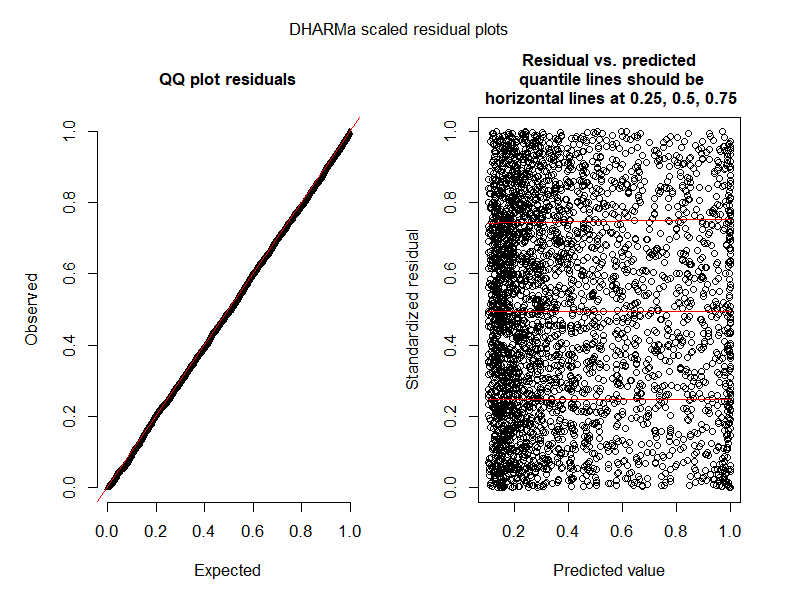

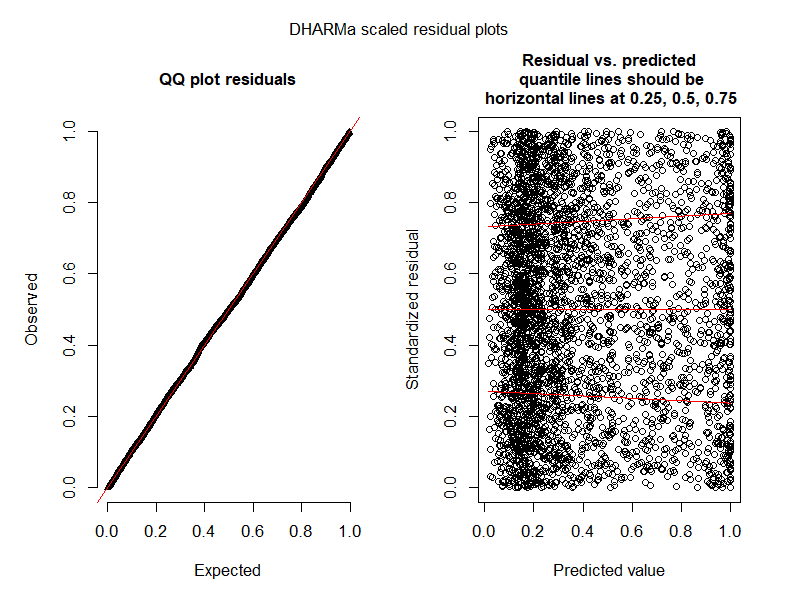

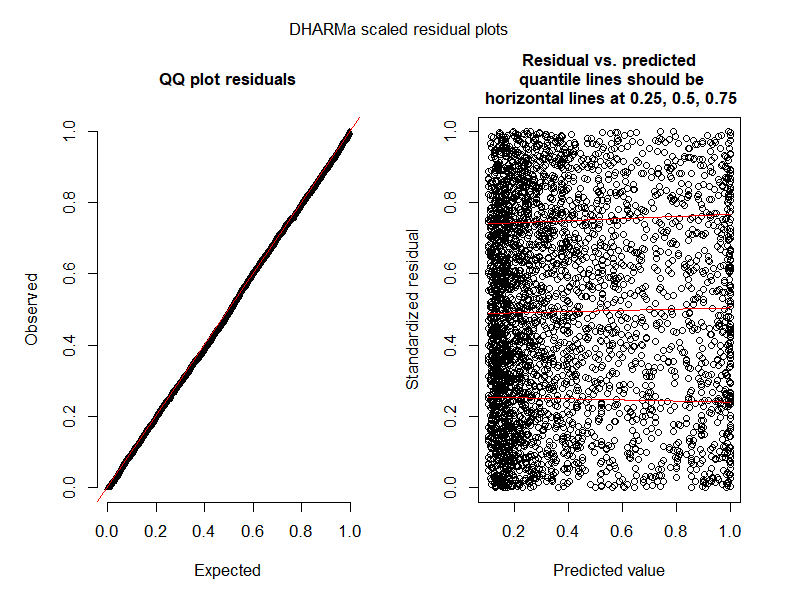

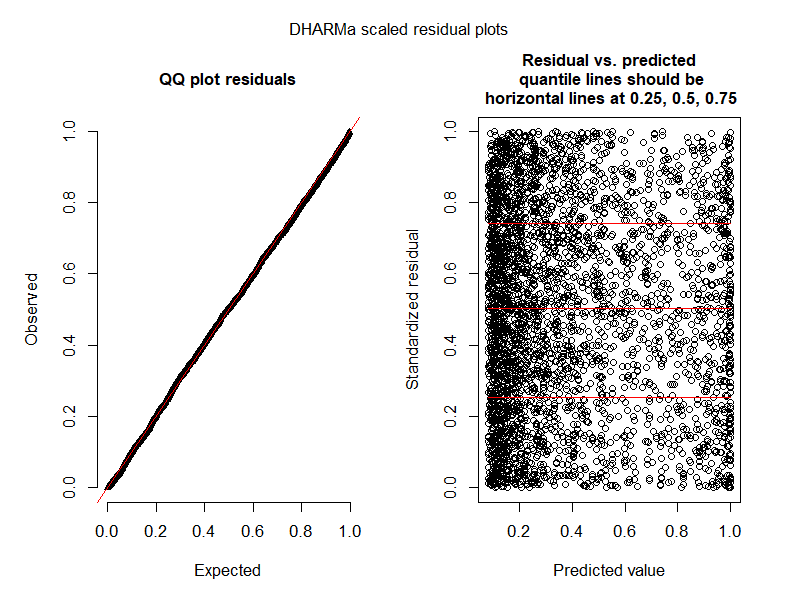

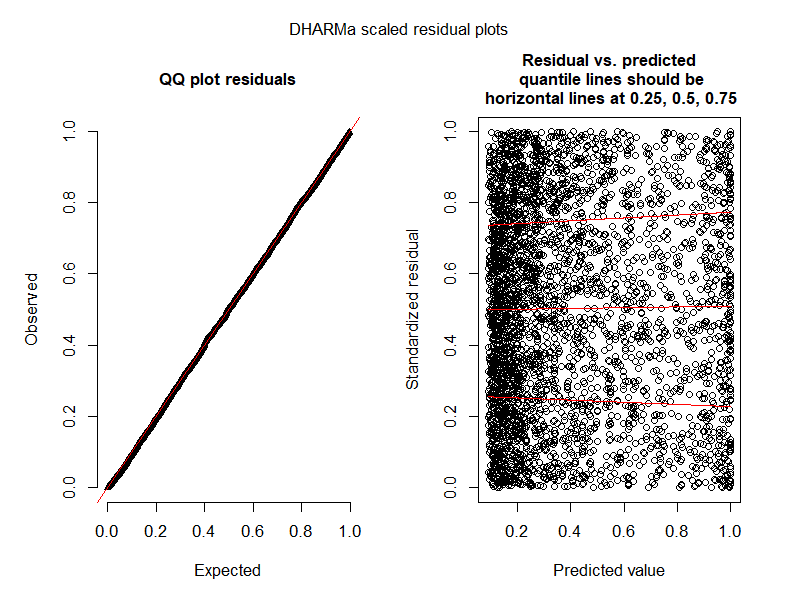

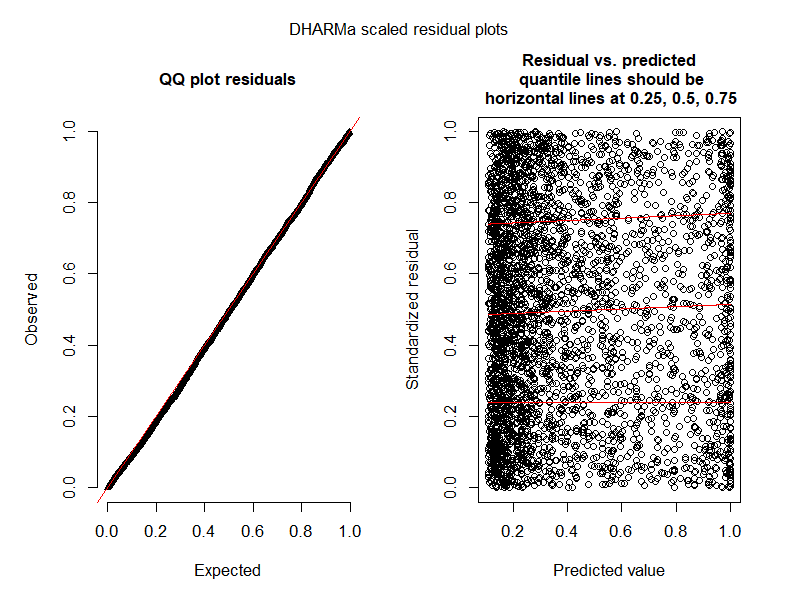

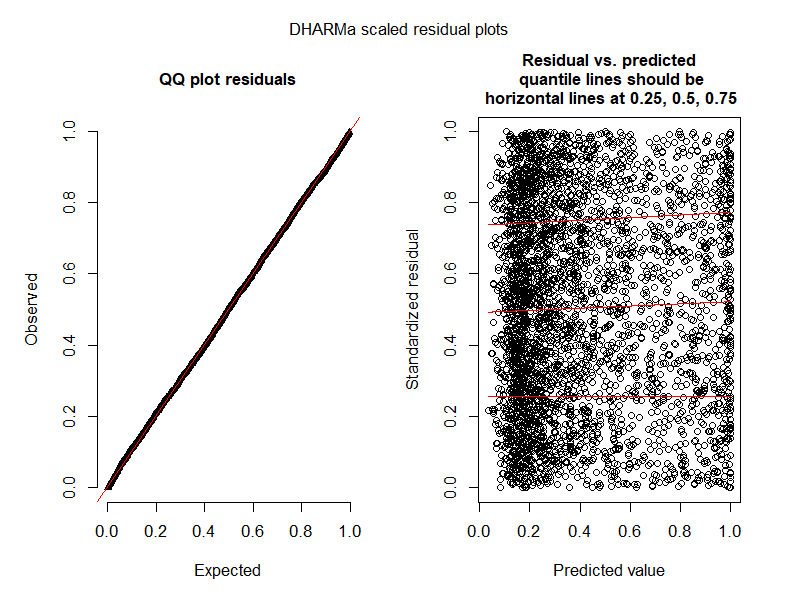

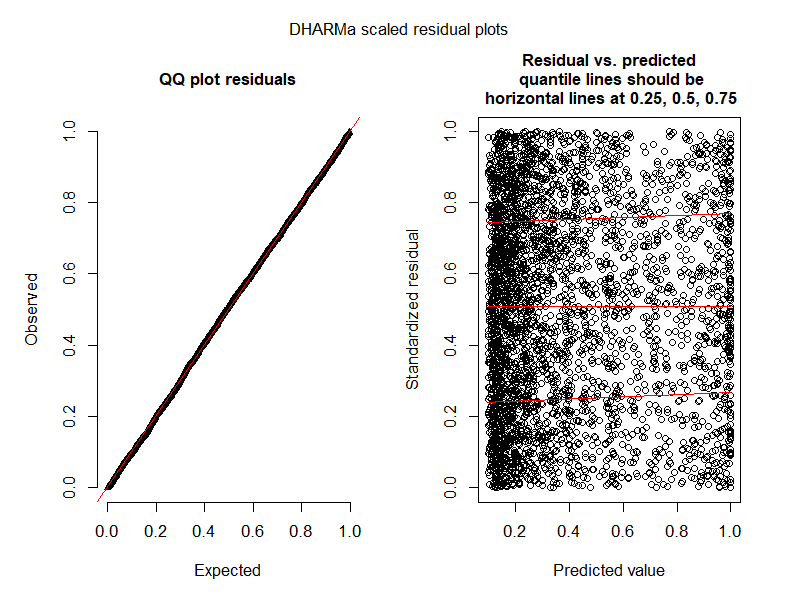

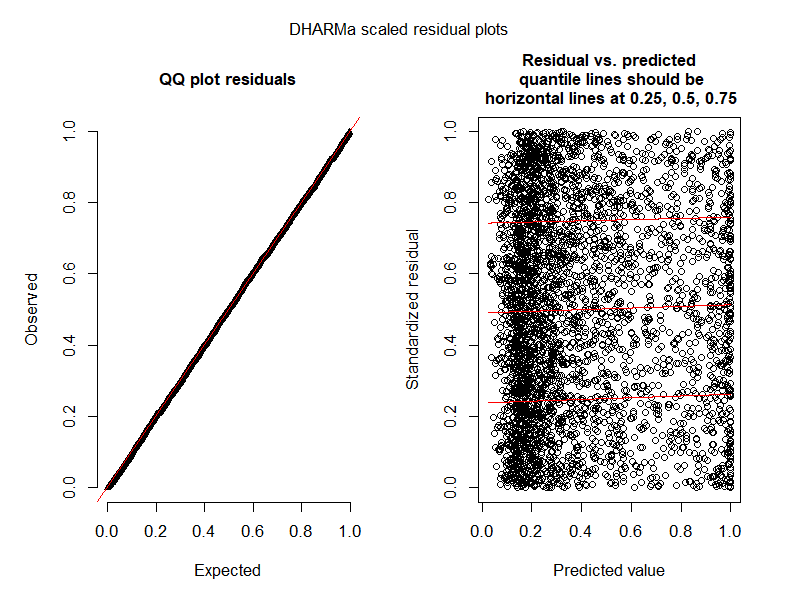

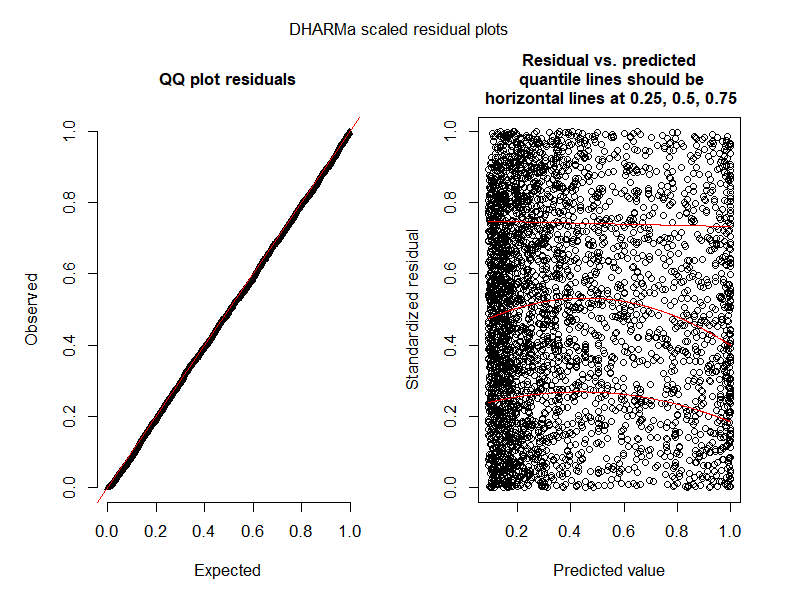

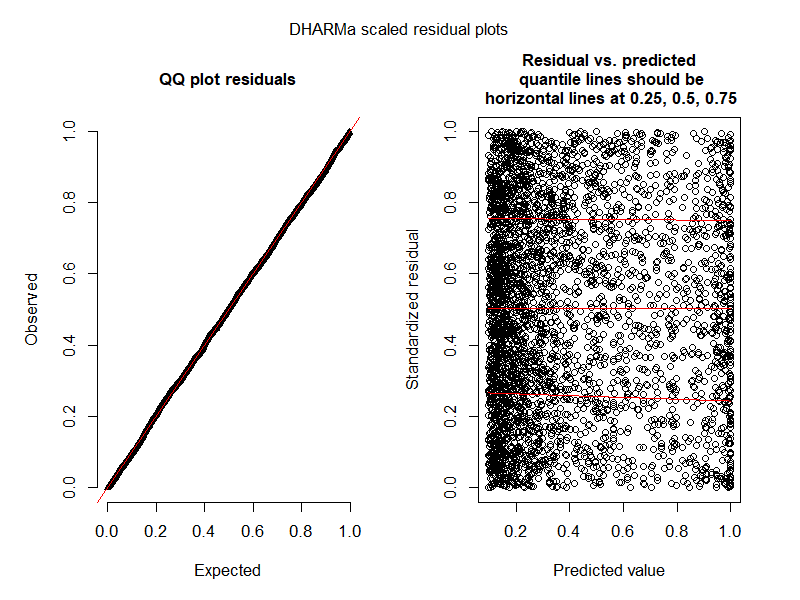

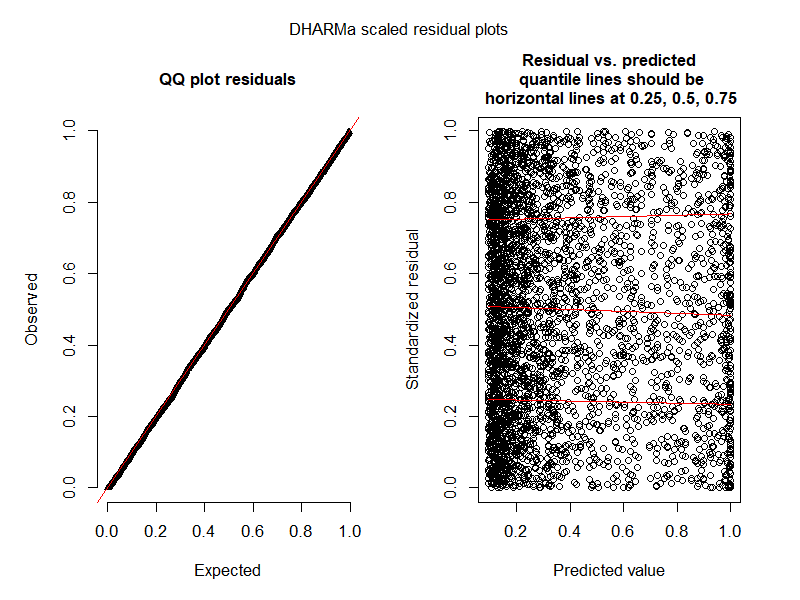

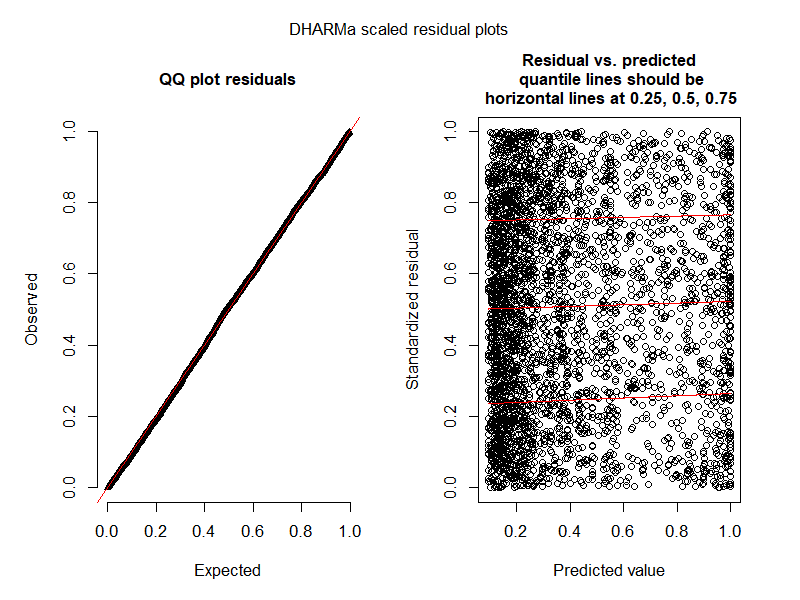


# Without dispersal limitation, without environmental filtering, high immigration rates


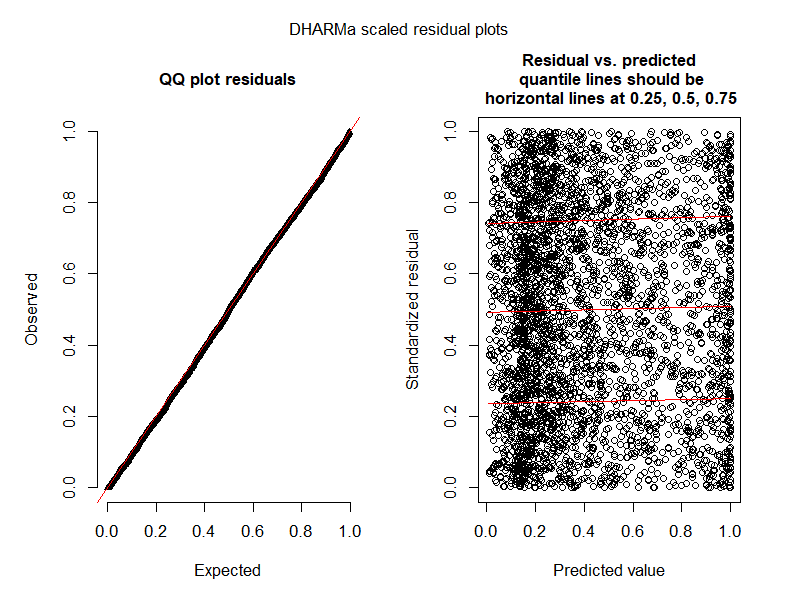

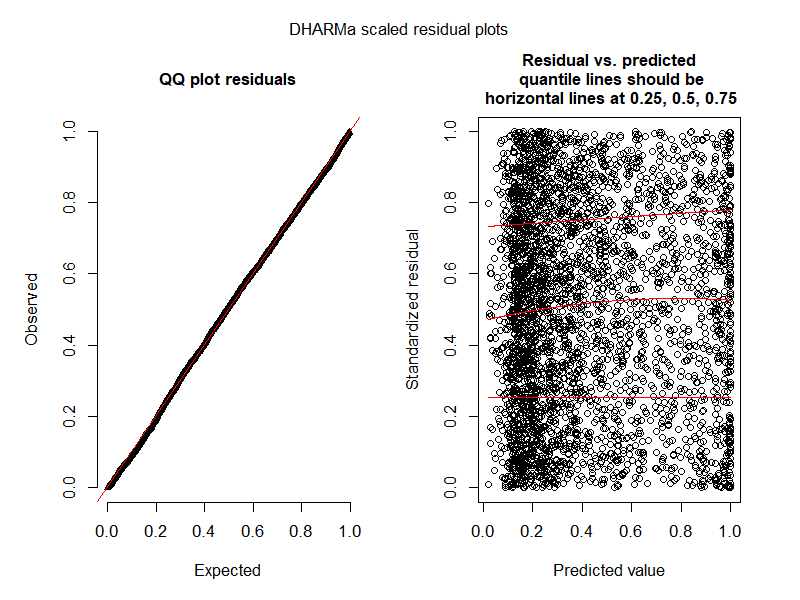

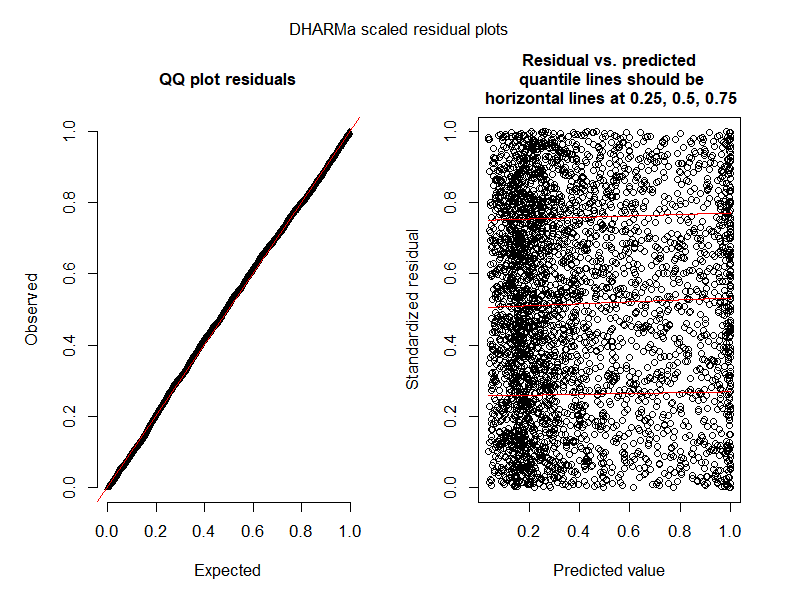

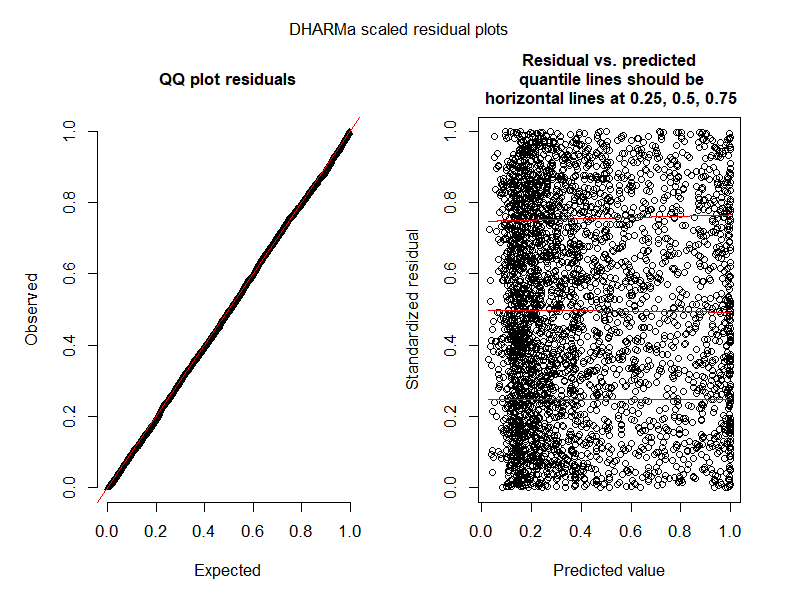

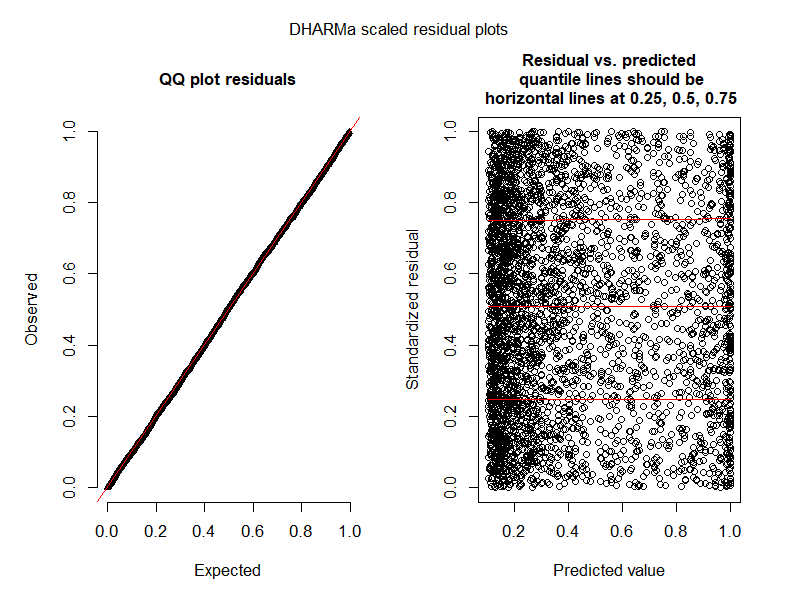

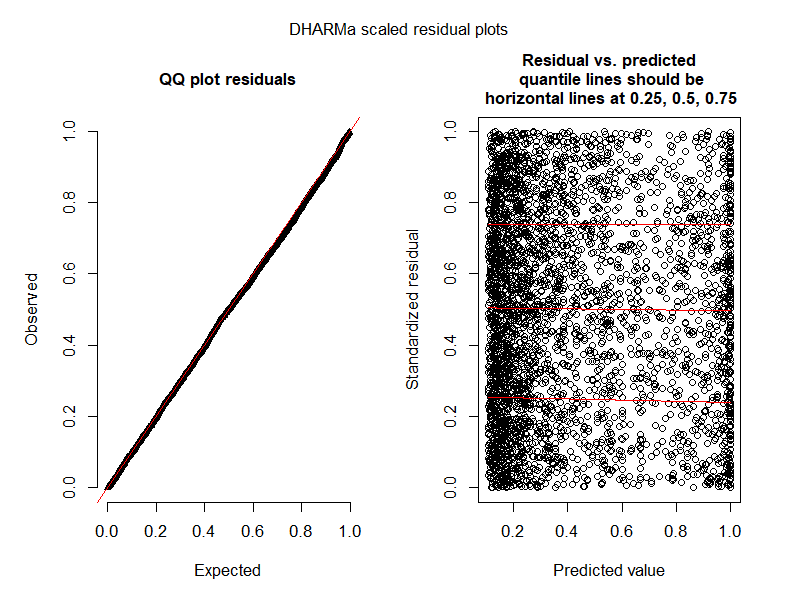

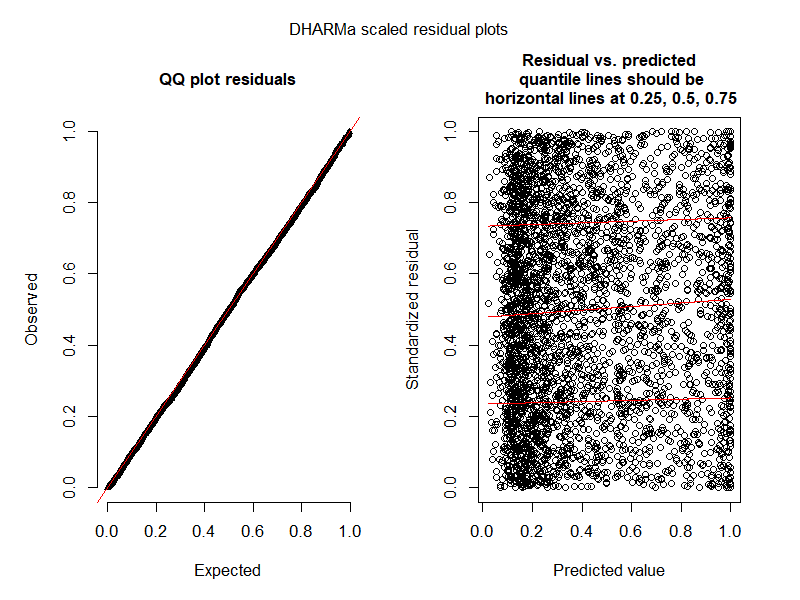

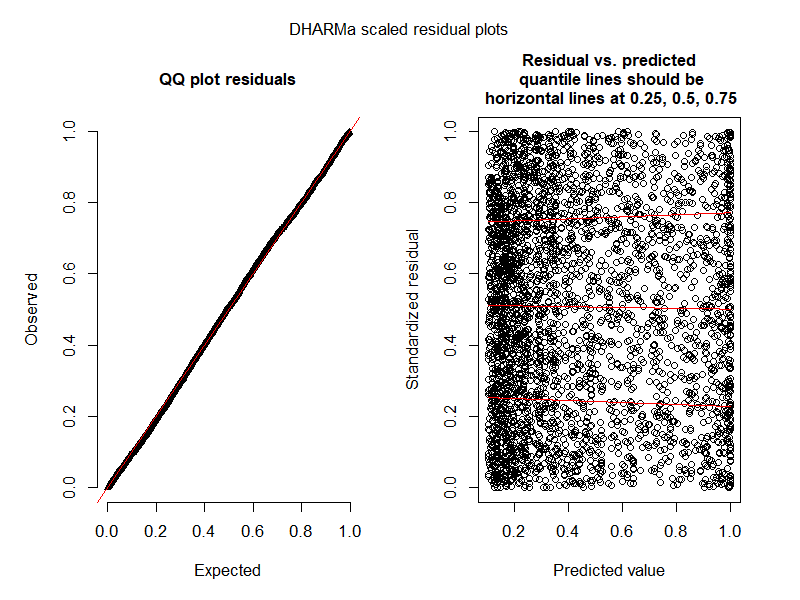

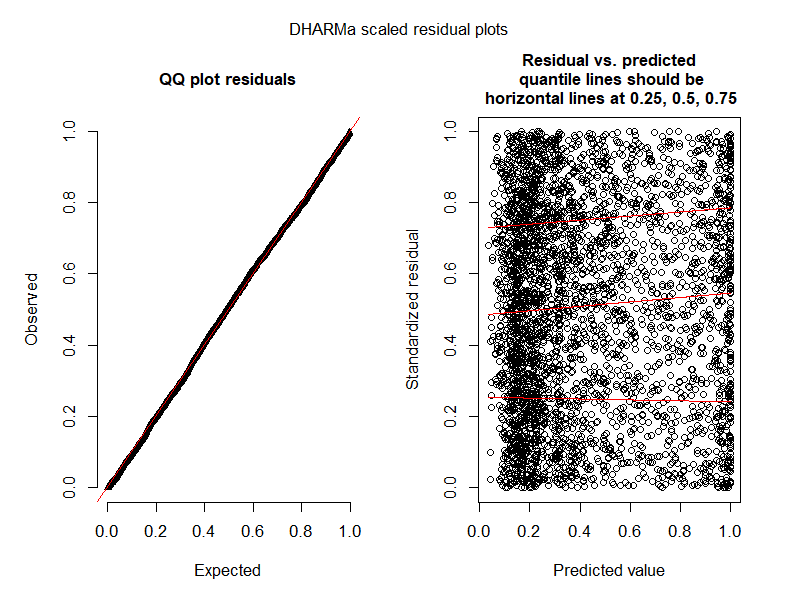

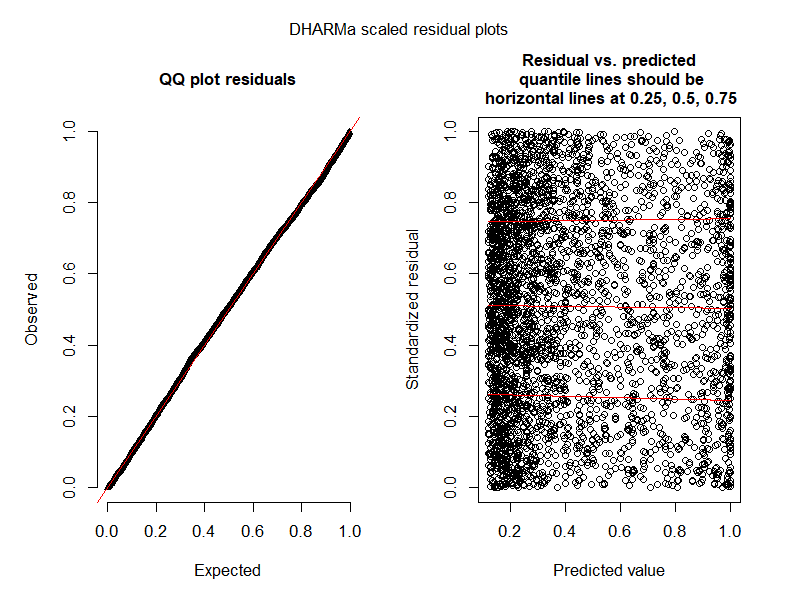

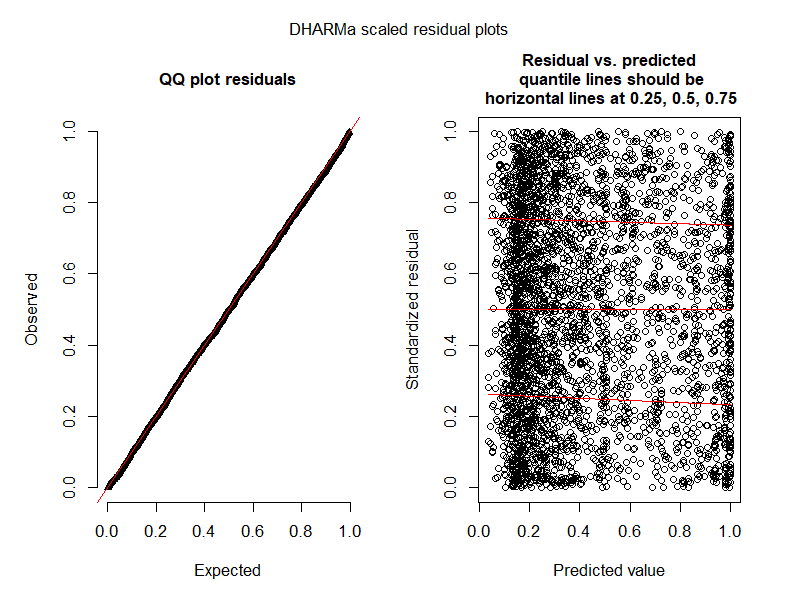

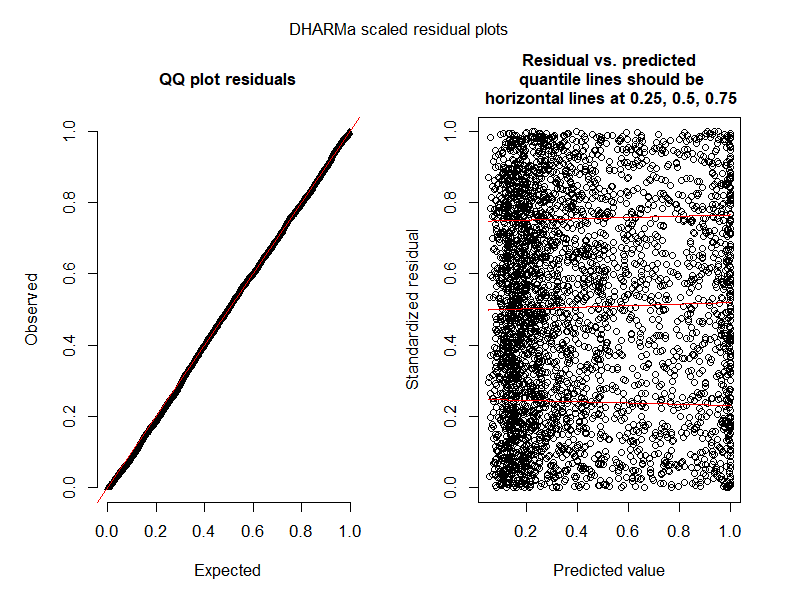

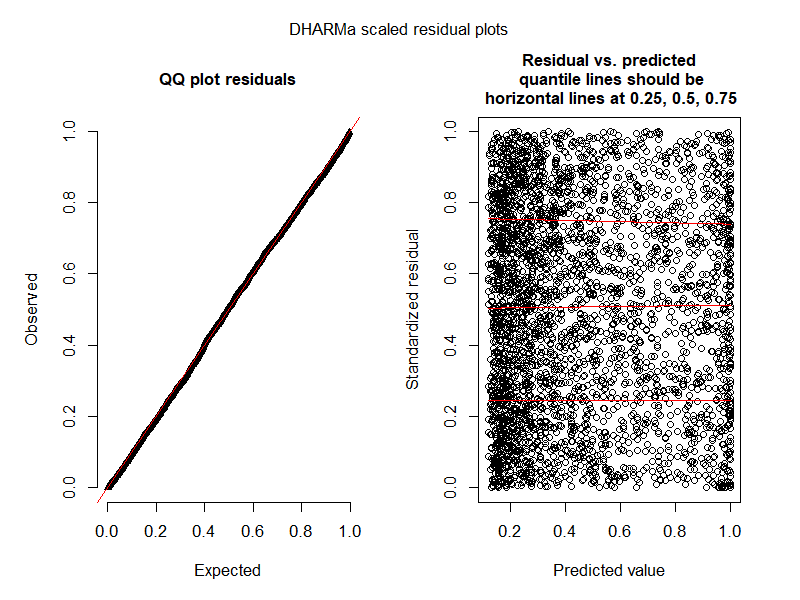

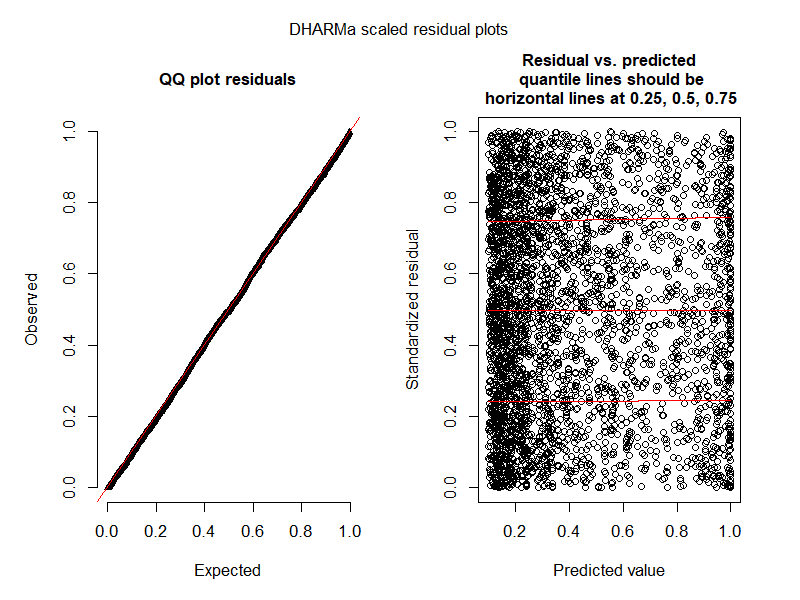

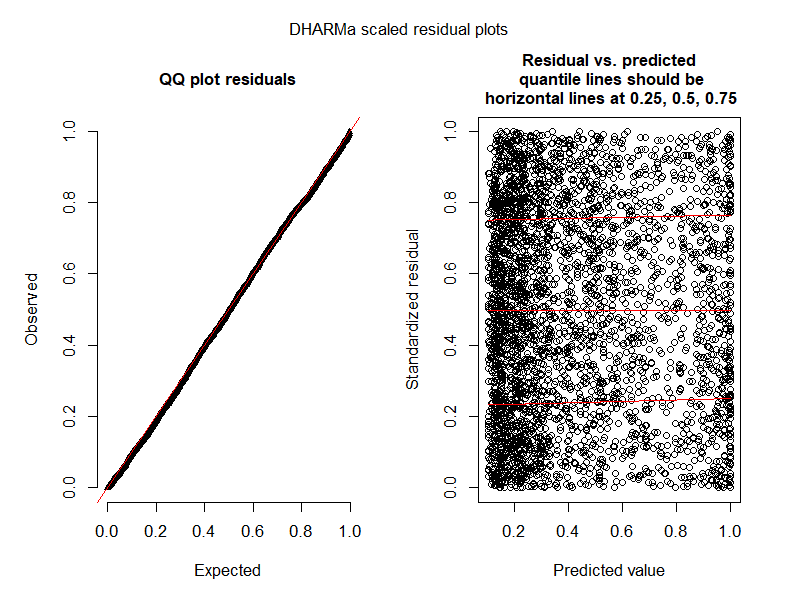

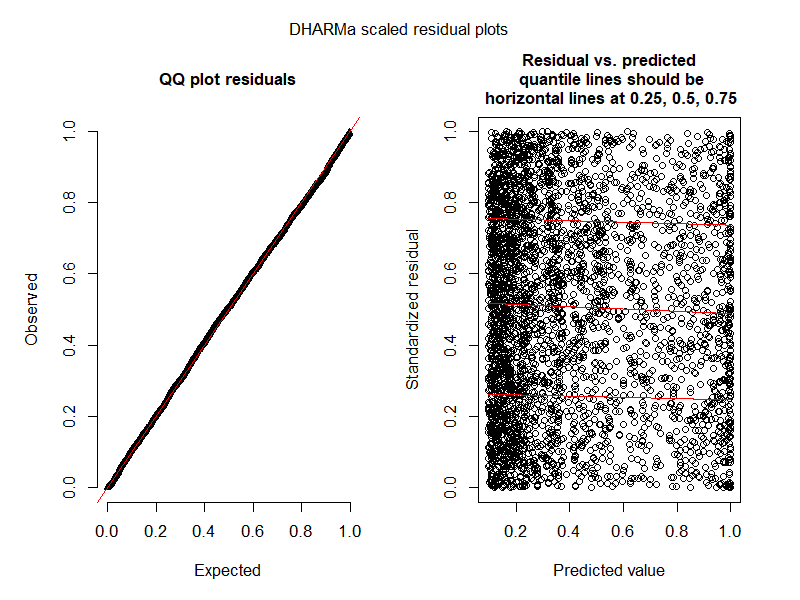

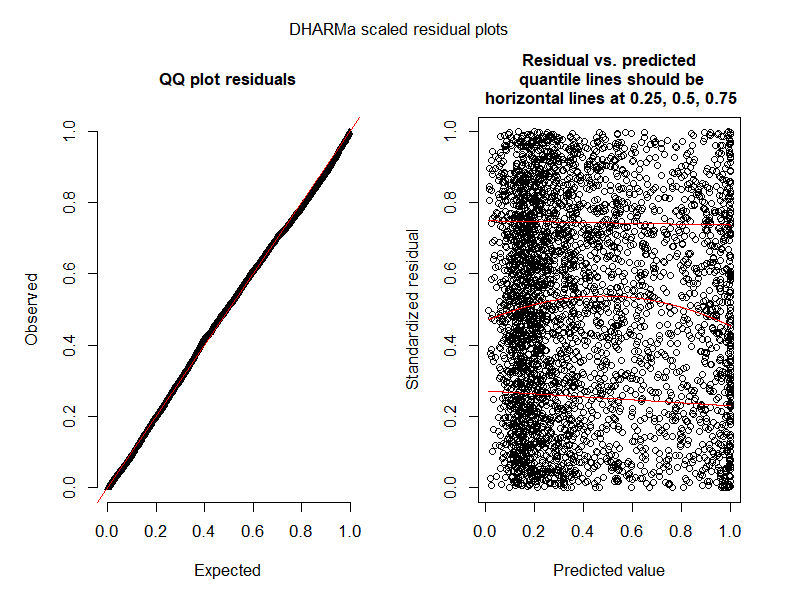

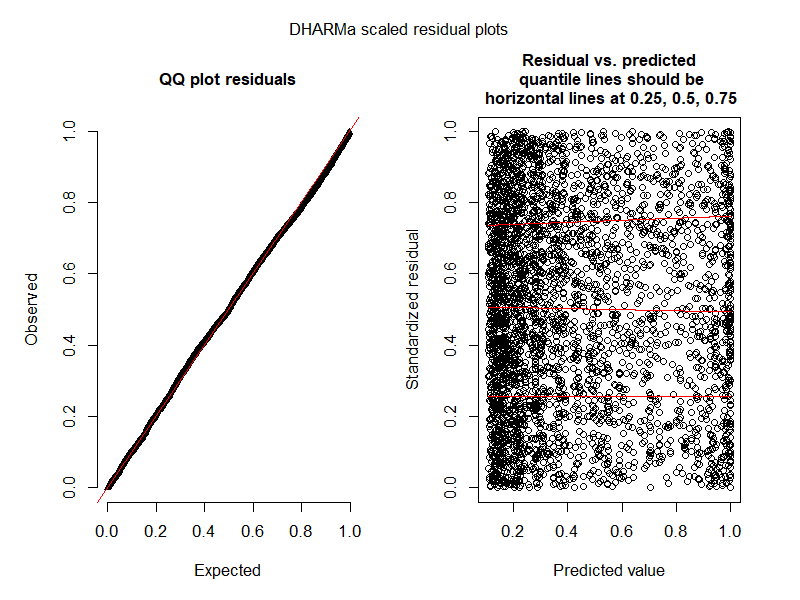

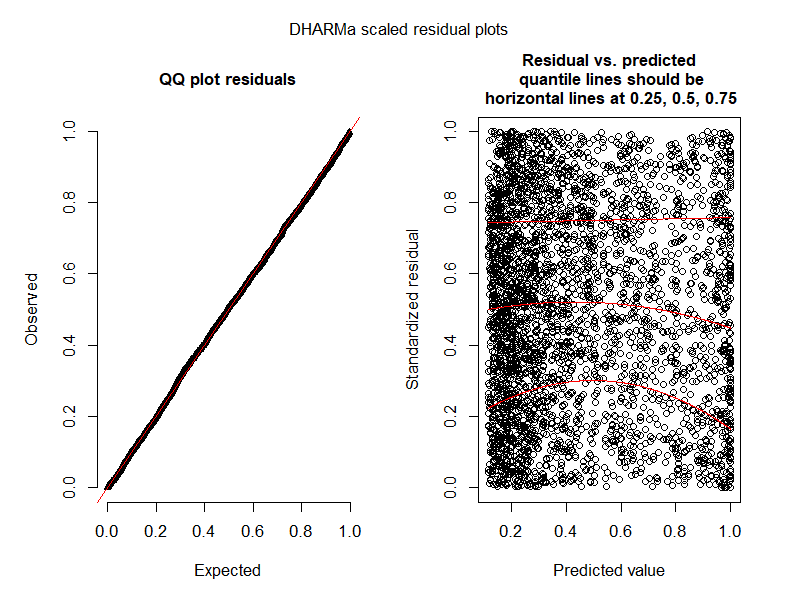

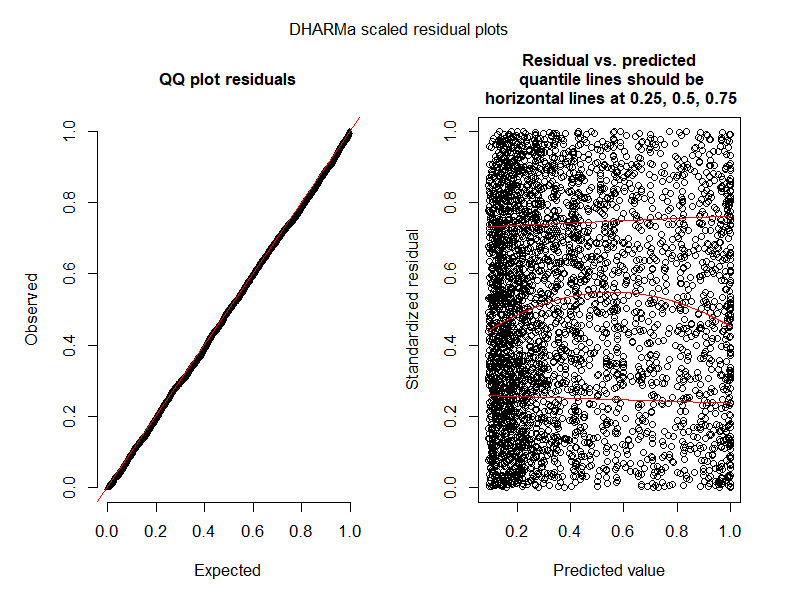

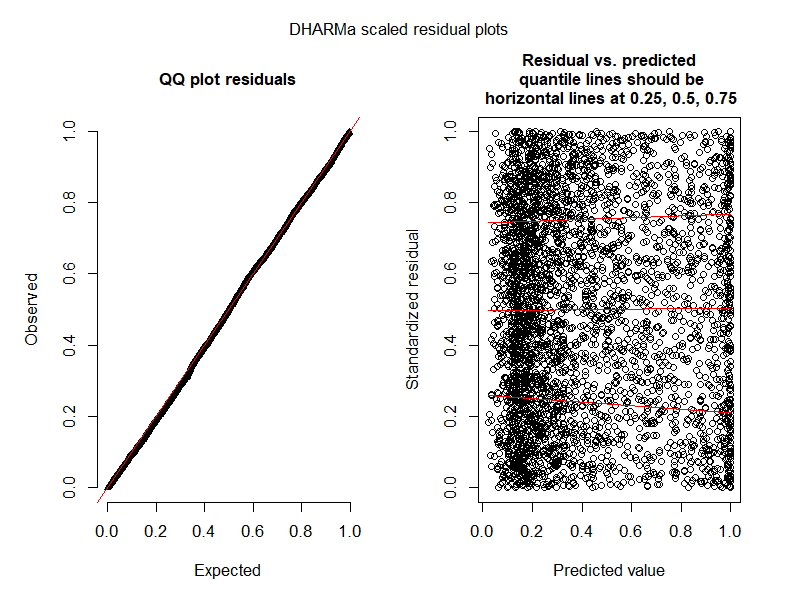

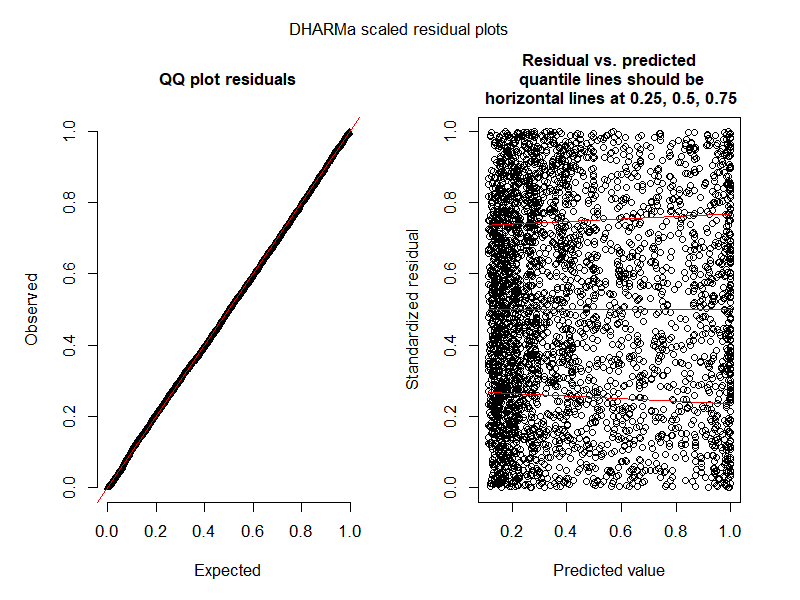

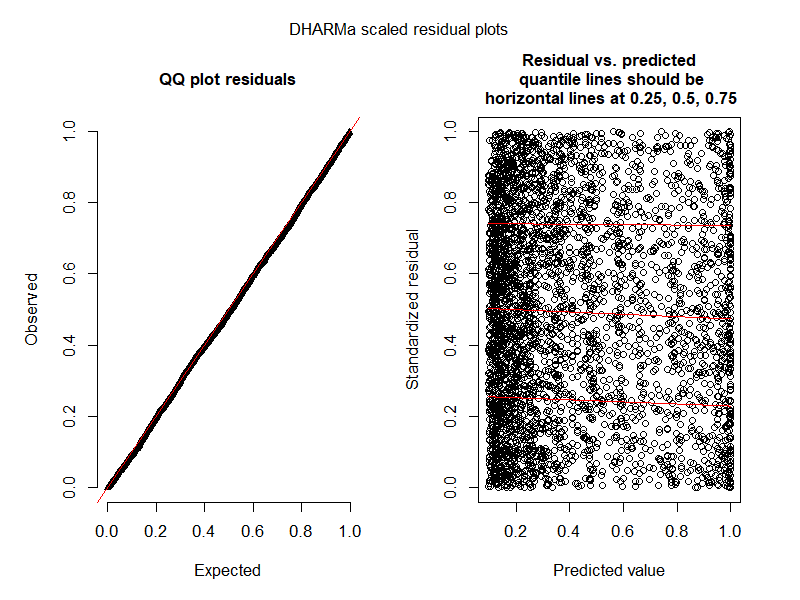

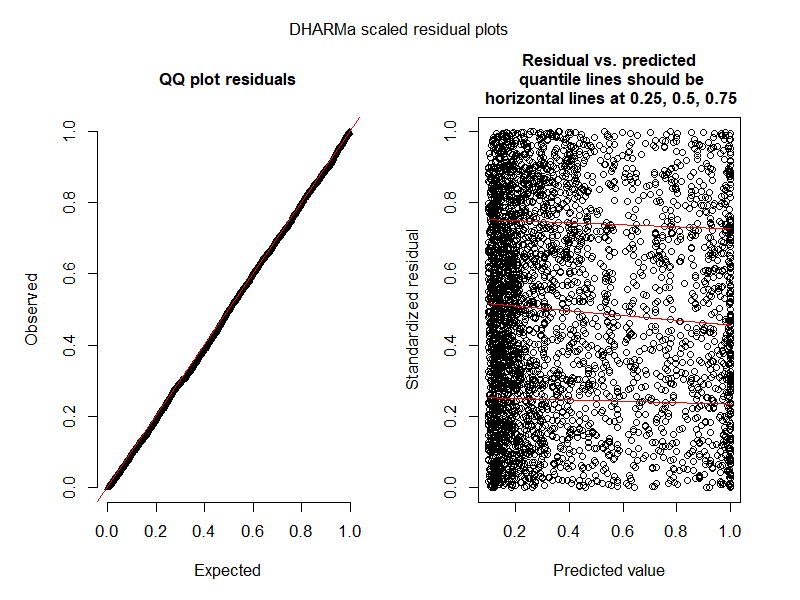

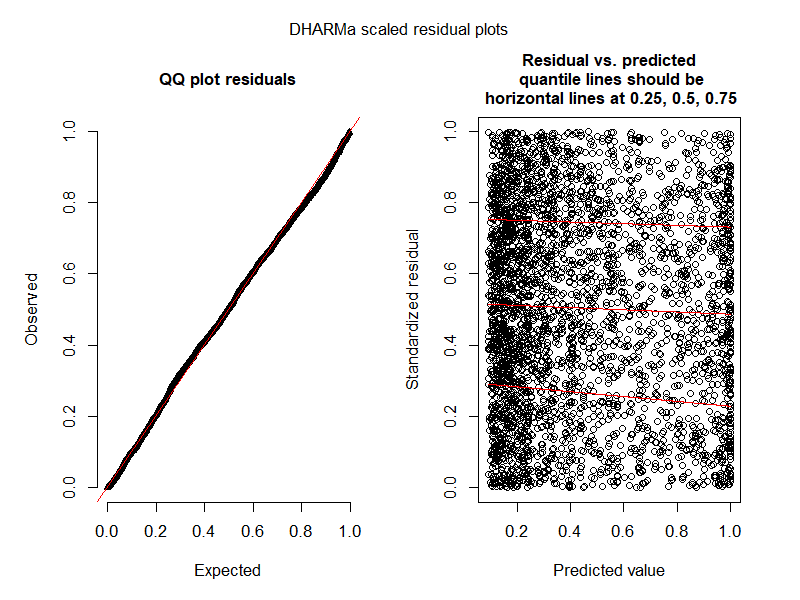


# Without dispersal limitation, with strong environmental filtering, low immigration rates


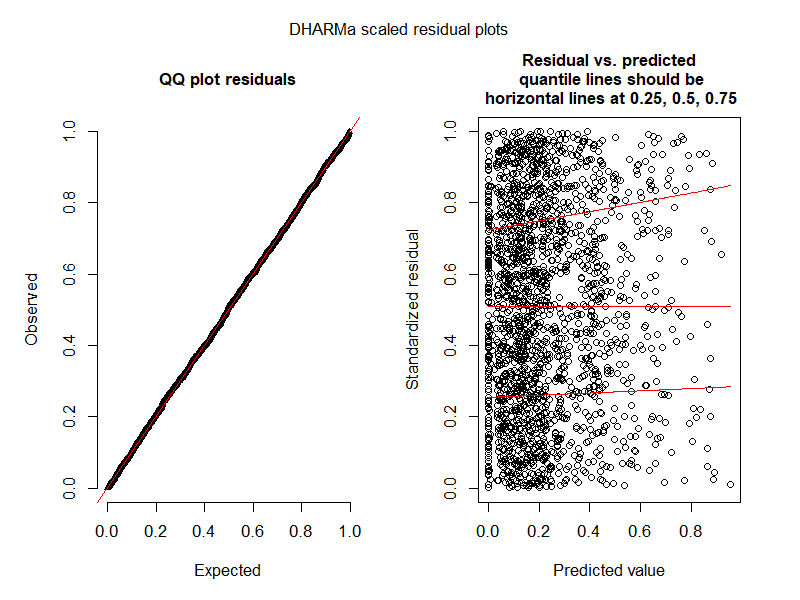

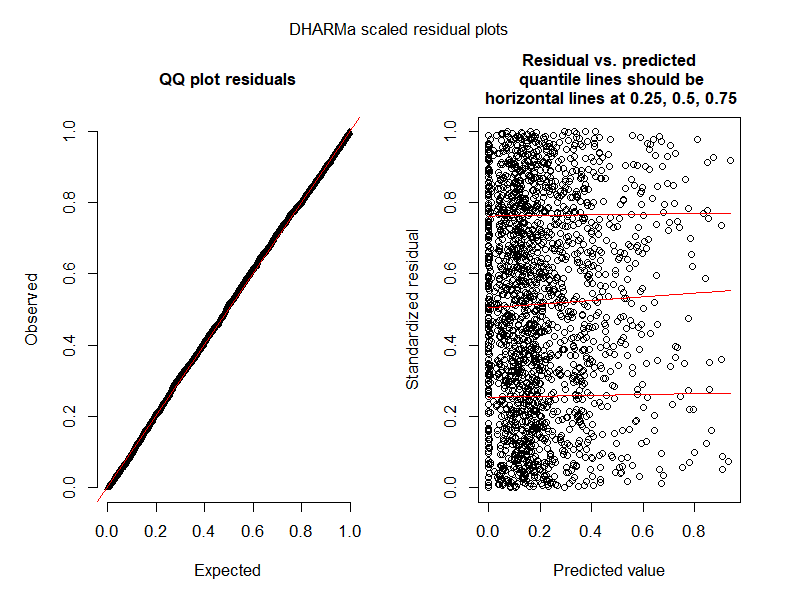

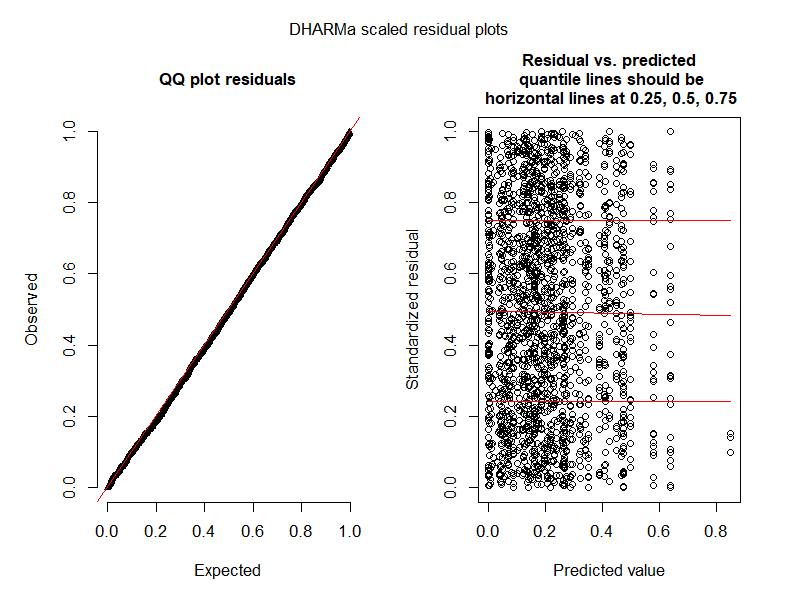

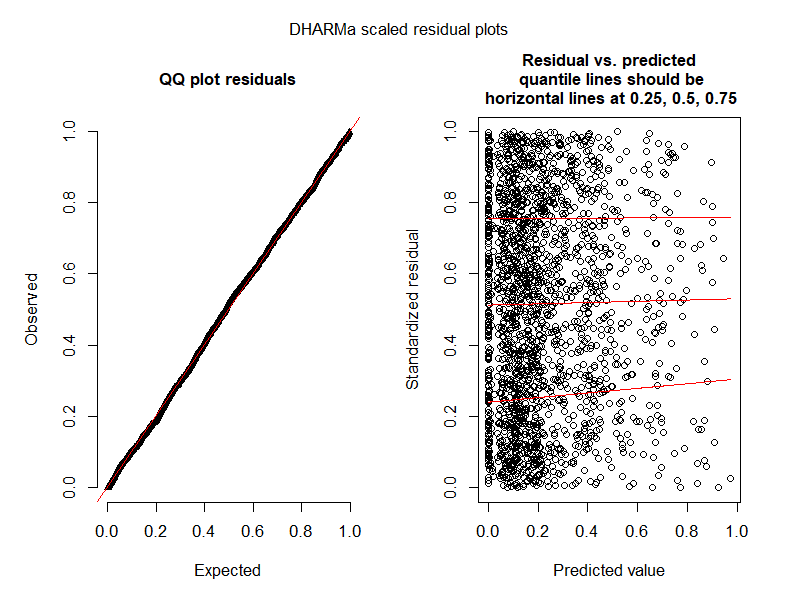

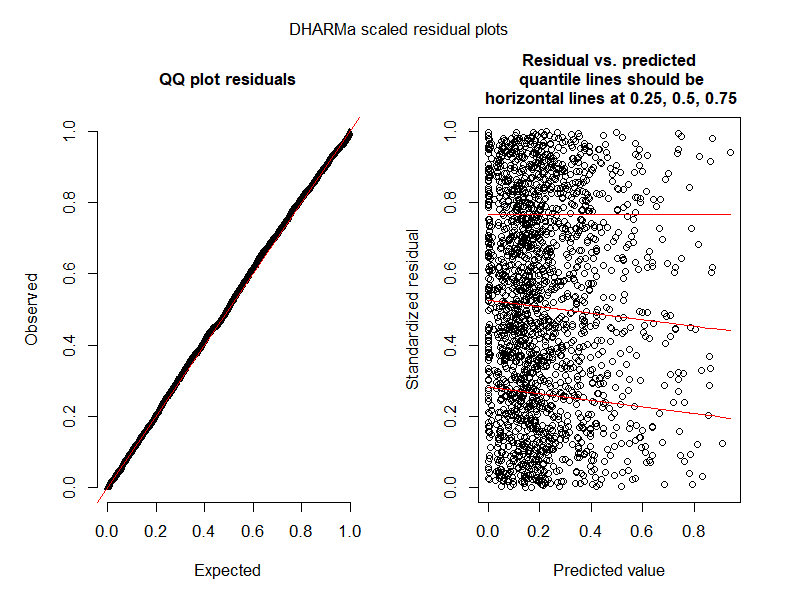

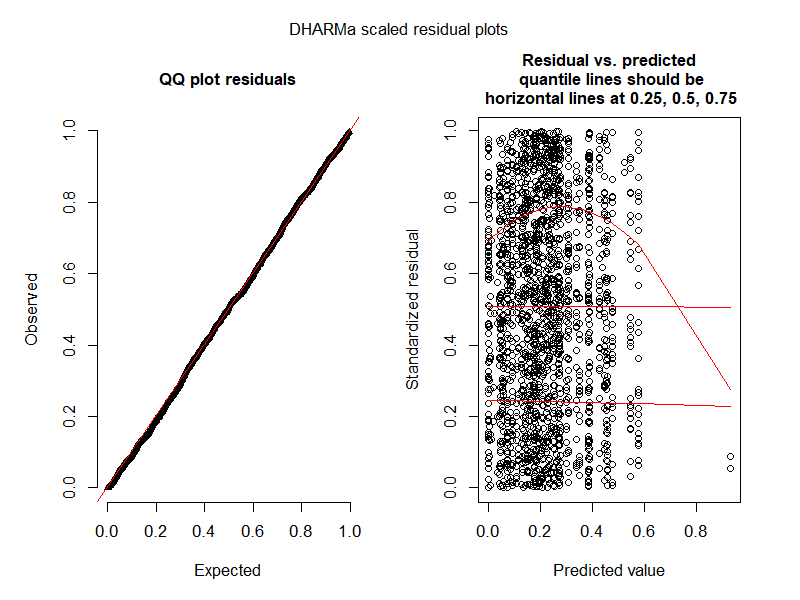

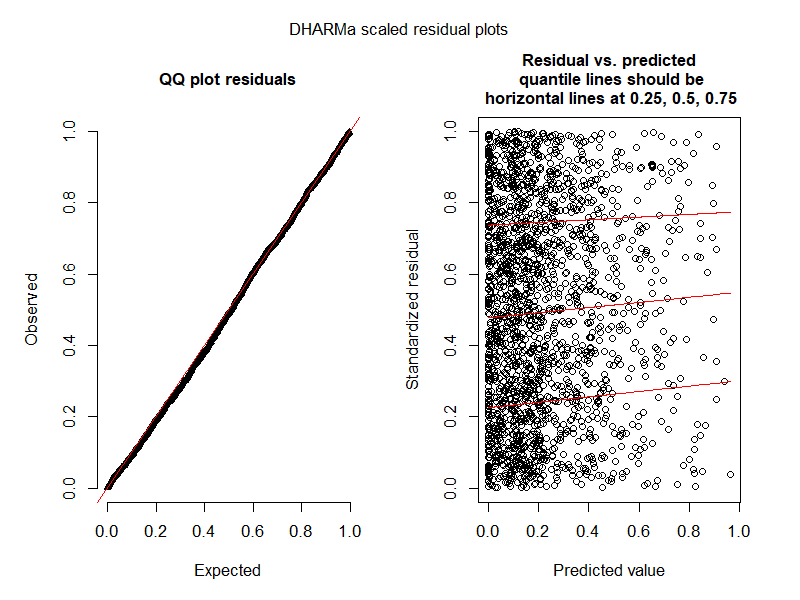

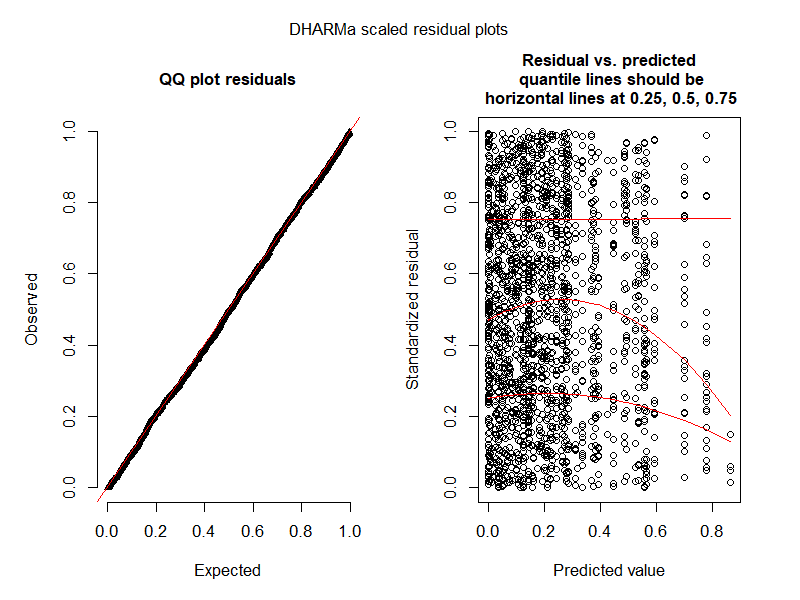

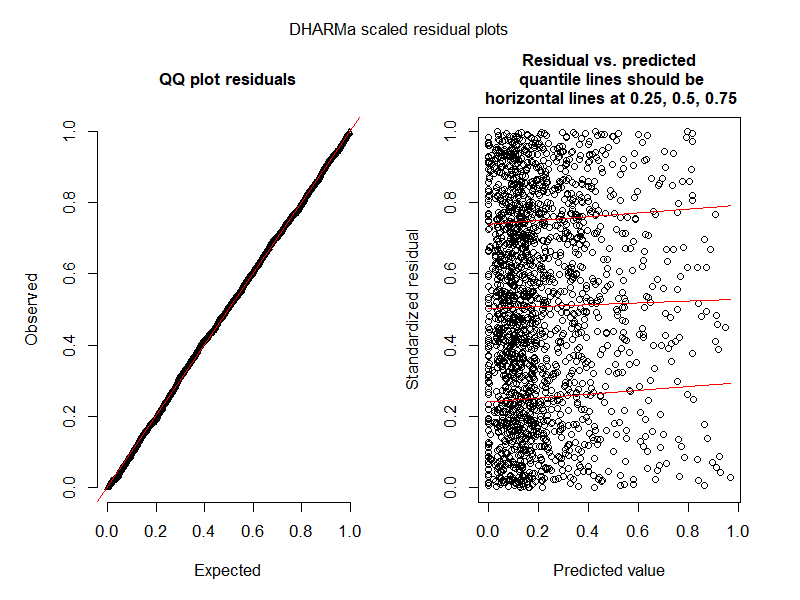

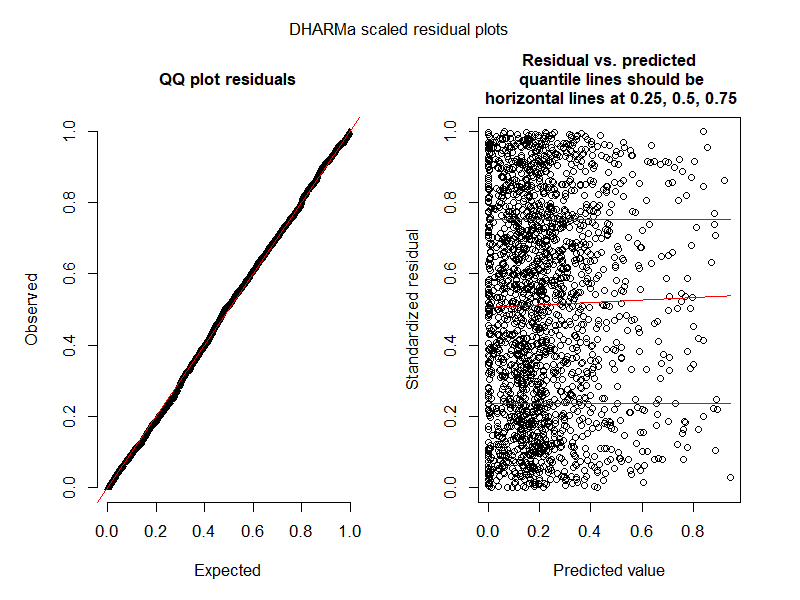

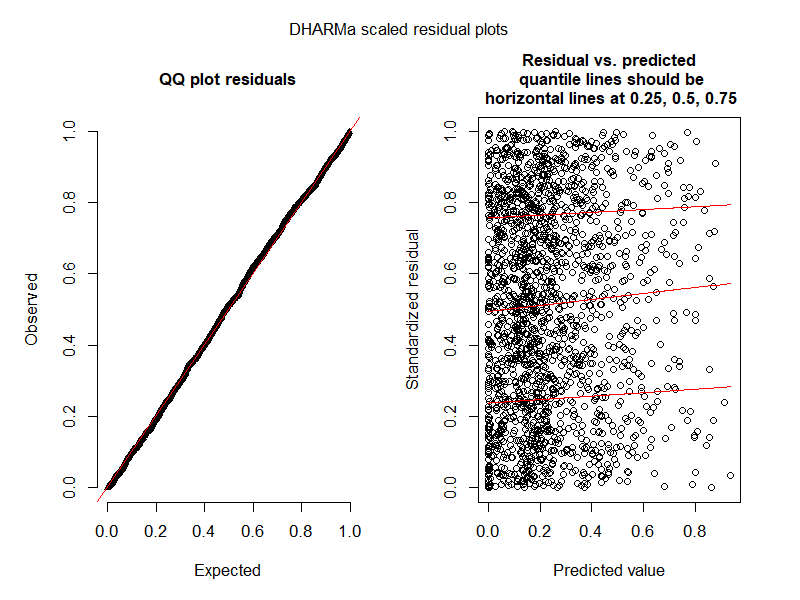

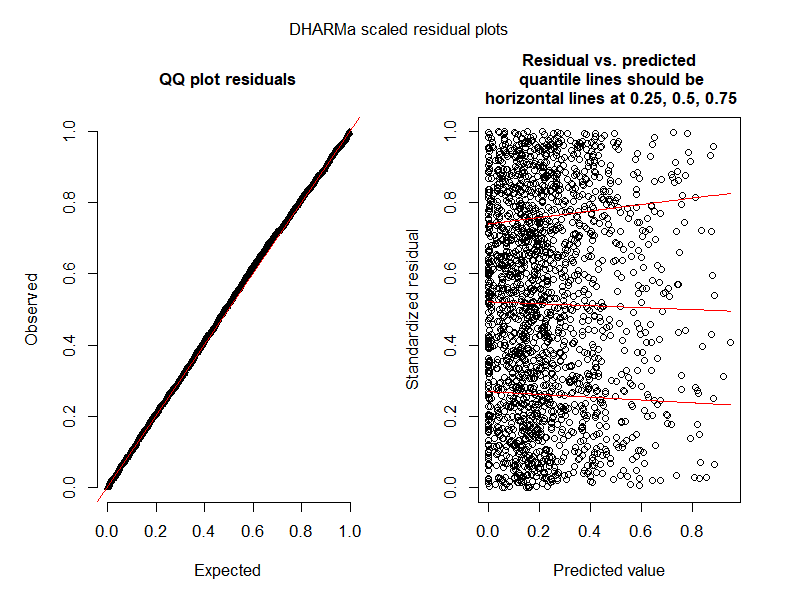

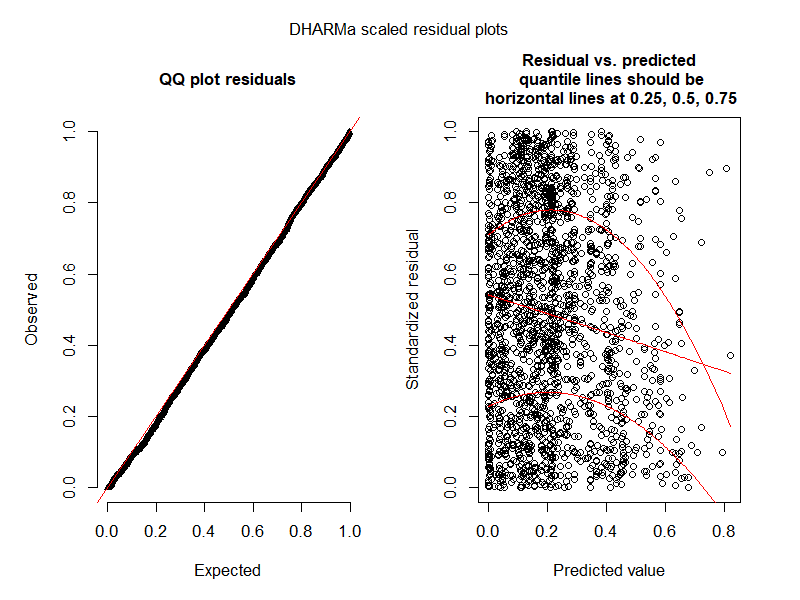

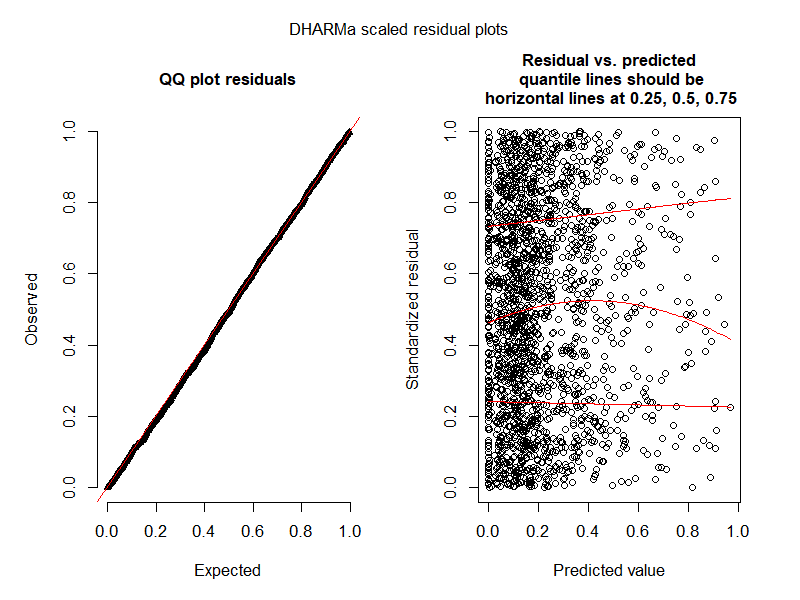

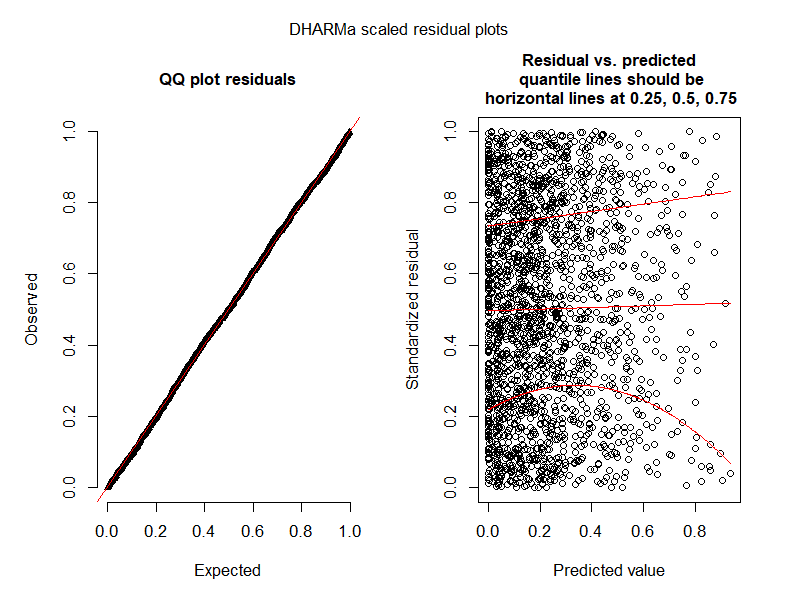

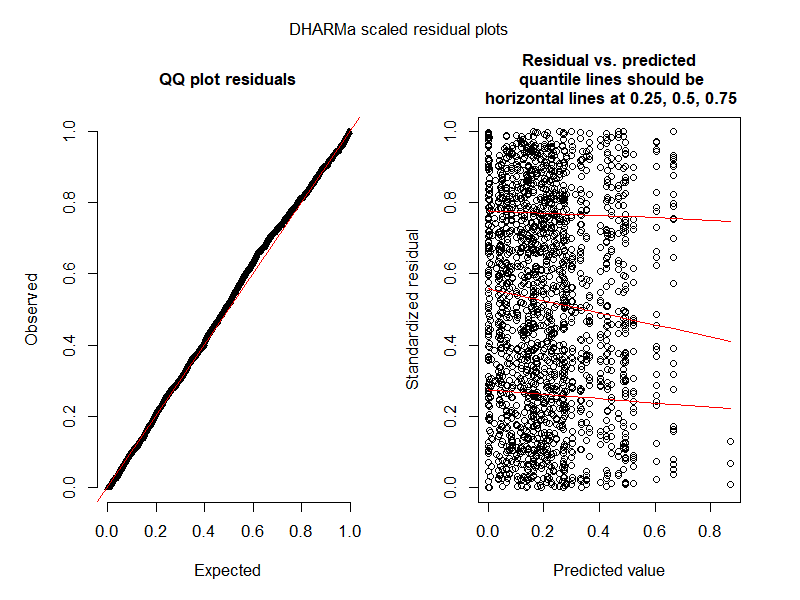

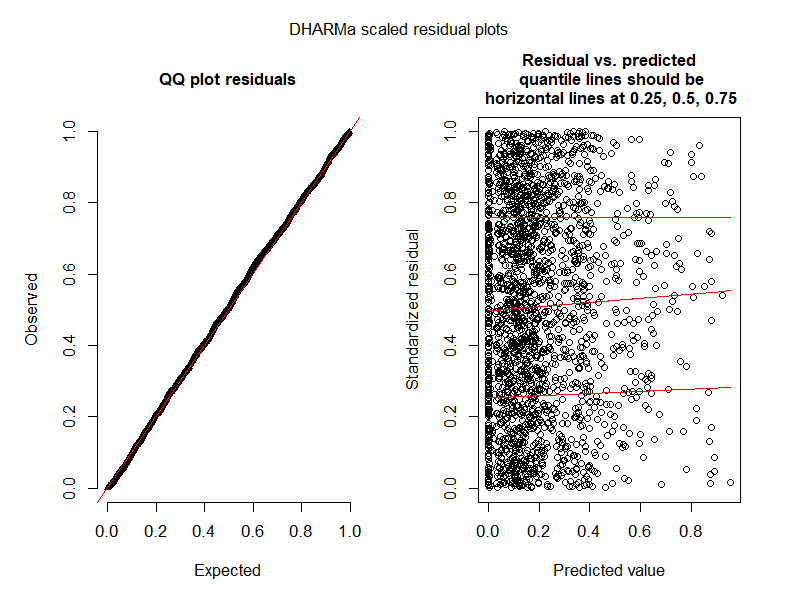

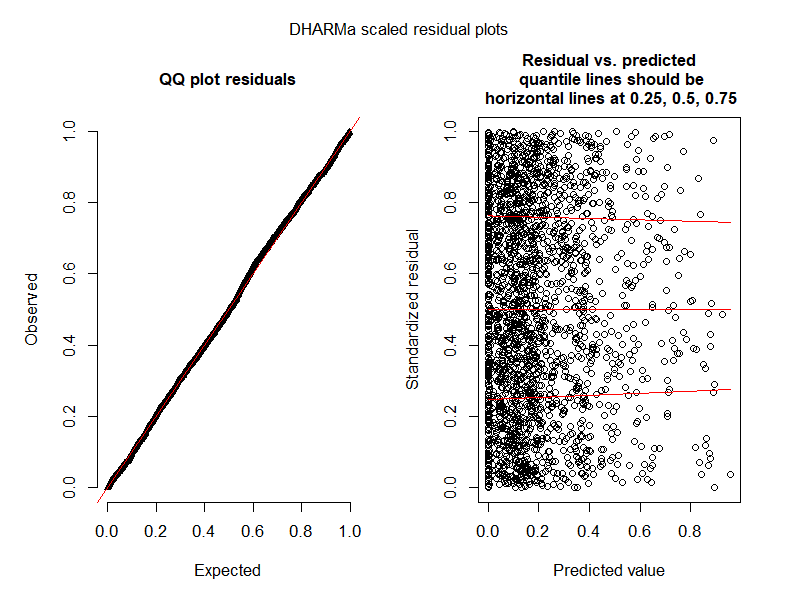

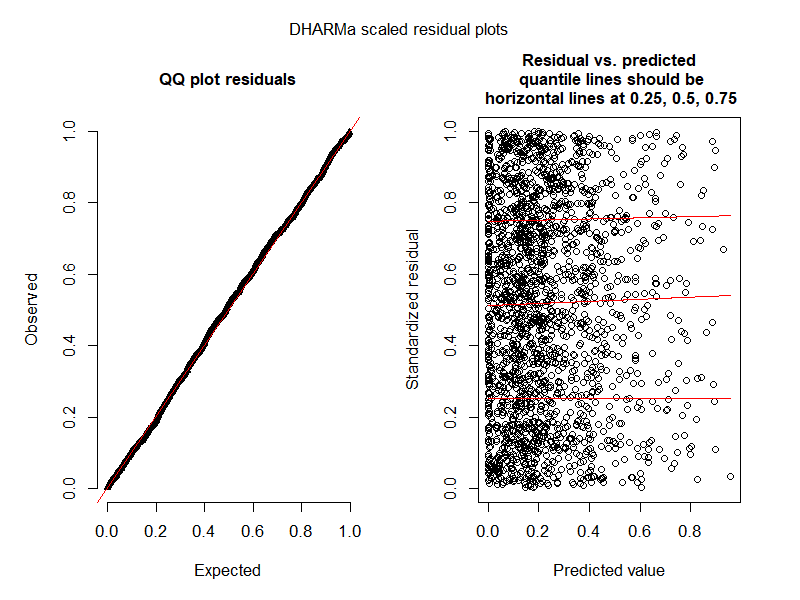

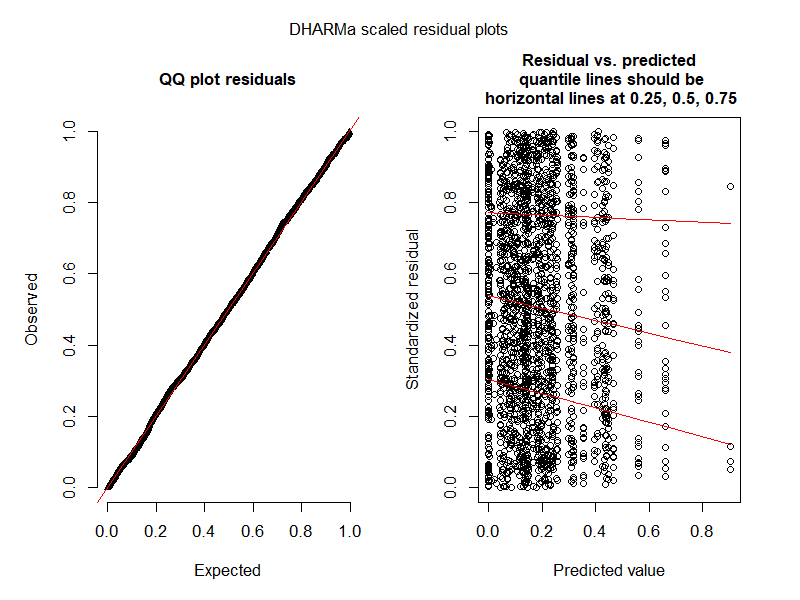

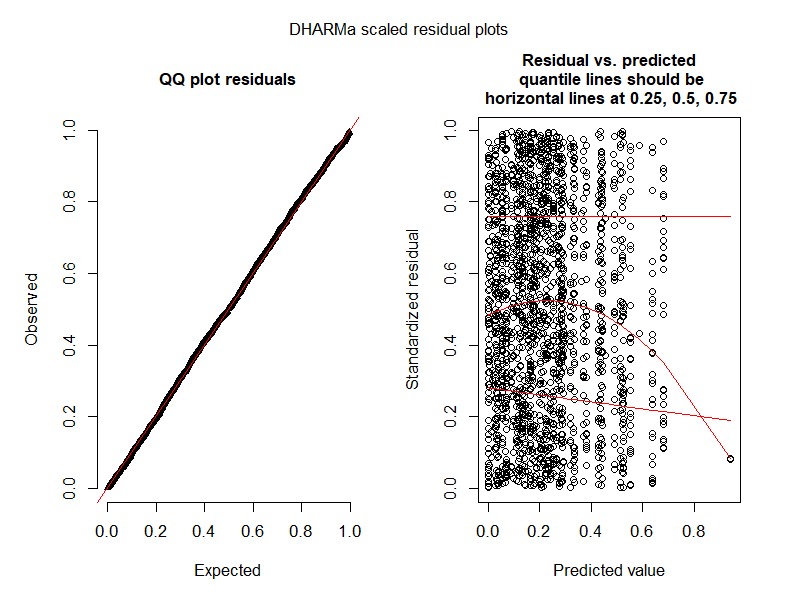

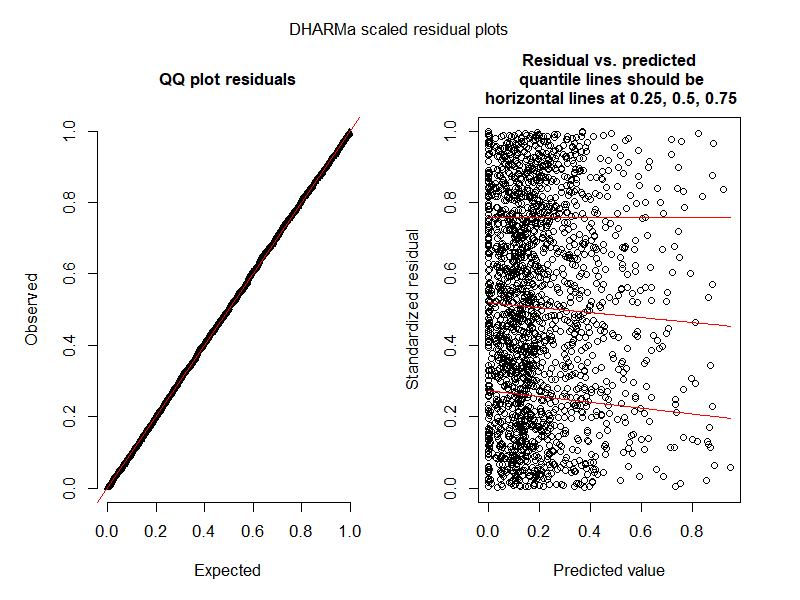

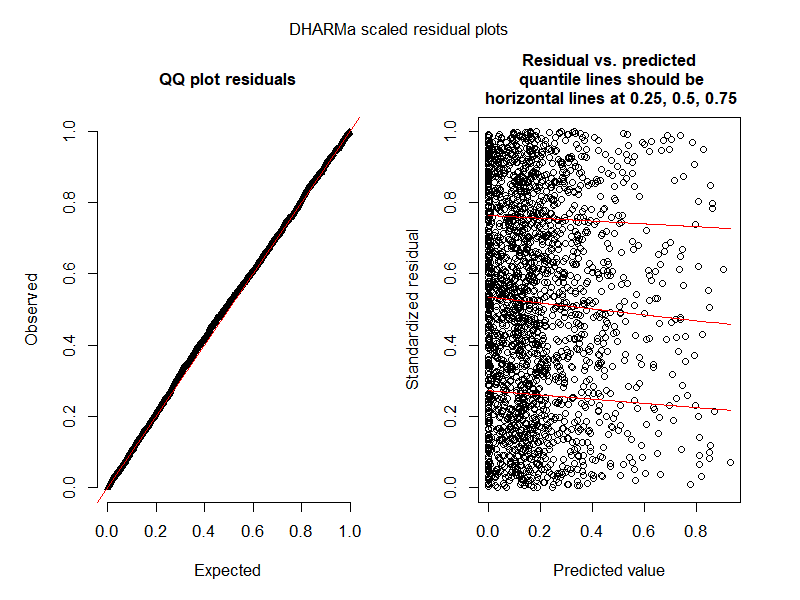

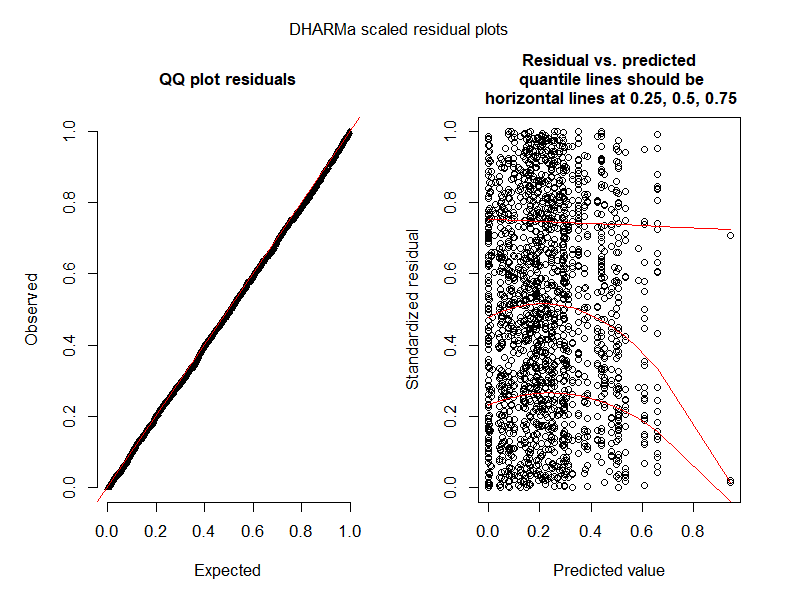

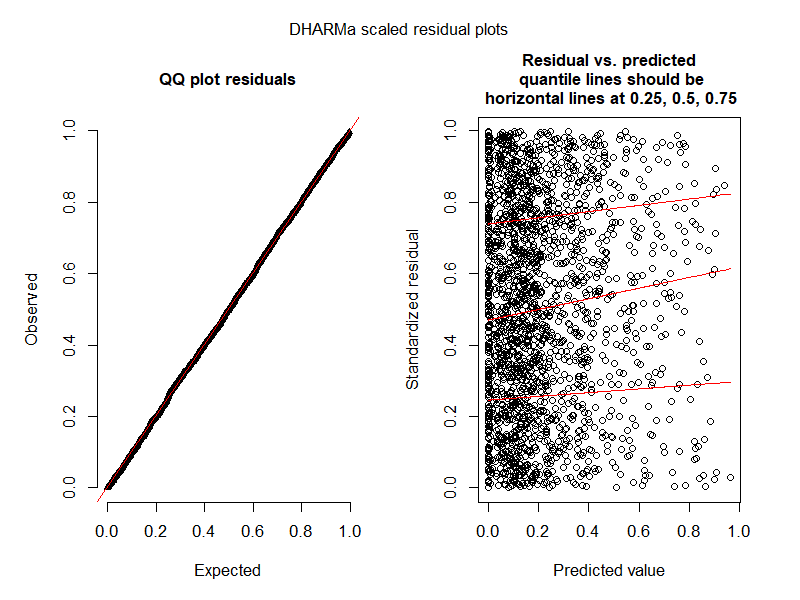


# Without dispersal limitation, with strong environmental filtering, intermediate immigration rates

# Without dispersal limitation, with strong environmental filtering, high immigration rates

# With dispersal limitation, without environmental filtering, low immigration rates

# With dispersal limitation, without environmental filtering, intermediate immigration rates

# With dispersal limitation, without environmental filtering, high immigration rates

# With dispersal limitation, with intermediate environmental filtering, low immigration rates

# With dispersal limitation, with intermediate environmental filtering, intermediate immigration rates

# With dispersal limitation, with intermediate environmental filtering, high immigration rates

# With dispersal limitation, with strong environmental filtering, low immigration rates

# With dispersal limitation, with strong environmental filtering, intermediate immigration rates

# With dispersal limitation, with strong environmental filtering, high immigration rates
